# Alcohol Exposure Induces Nucleolar Stress and Apoptosis in Mouse Neural Stem Cells and Late-Term Fetal Brain

**DOI:** 10.3390/cells13050440

**Published:** 2024-03-02

**Authors:** Yanping Huang, George R. Flentke, Olivia C. Rivera, Nipun Saini, Sandra M. Mooney, Susan M. Smith

**Affiliations:** 1 UNC Nutrition Research Institute, University of North Carolina at Chapel Hill, Kannapolis, NC 28081, USAnipun_saini@unc.edu (N.S.); sandra_mooney@unc.edu (S.M.M.); 2 Department of Nutrition, University of North Carolina at Chapel Hill, Kannapolis, NC 28081, USA

**Keywords:** apoptosis, fetal alcohol spectrum disorder, neural stem cells, nucleolar stress, MDM2, p53, proliferation, ribosome biogenesis, ribosomopathy

## Abstract

Prenatal alcohol exposure (PAE) is a leading cause of neurodevelopmental disability through its induction of neuronal growth dysfunction through incompletely understood mechanisms. Ribosome biogenesis regulates cell cycle progression through p53 and the nucleolar cell stress response. Whether those processes are targeted by alcohol is unknown. Pregnant C57BL/6J mice received 3 g alcohol/kg daily at E8.5–E17.5. Transcriptome sequencing was performed on the E17.5 fetal cortex. Additionally, primary neural stem cells (NSCs) were isolated from the E14.5 cerebral cortex and exposed to alcohol to evaluate nucleolar stress and p53/MDM2 signaling. Alcohol suppressed KEGG pathways involving ribosome biogenesis (rRNA synthesis/processing and ribosomal proteins) and genes that are mechanistic in ribosomopathies (*Polr1d*, *Rpl11*; *Rpl35*; *Nhp2*); this was accompanied by nucleolar dissolution and p53 stabilization. In primary NSCs, alcohol reduced rRNA synthesis, caused nucleolar loss, suppressed proliferation, stabilized nuclear p53, and caused apoptosis that was prevented by dominant-negative p53 and MDM2 overexpression. Alcohol’s actions were dose-dependent and rapid, and rRNA synthesis was suppressed between 30 and 60 min following alcohol exposure. The alcohol-mediated deficits in ribosomal protein expression were correlated with fetal brain weight reductions. This is the first report describing that pharmacologically relevant alcohol levels suppress ribosome biogenesis, induce nucleolar stress in neuronal populations, and involve the ribosomal/MDM2/p53 pathway to cause growth arrest and apoptosis. This represents a novel mechanism of alcohol-mediated neuronal damage.

## 1. Introduction

Prenatal alcohol exposure (PAE) causes a range of deficits affecting growth, craniofacial development, and domains of behavior and cognition under the diagnostic framework of fetal alcohol spectrum disorders (FASD) [1]. Between 1–5% of U.S. first graders meet the diagnostic criteria for FASD [2]. Of these, behavioral and cognitive deficits have the most adverse impact on quality of life [3,4]. Alcohol has complex effects on brain development that are shaped by the dose and timing of exposure and include disruptions in neuronal expansion, differentiation, survival, migration, synaptogenesis, and axon formation, with a particular impact observed in the cerebral cortex [5]. Neural stem cells (NSCs) appear to be a target of PAE, with reduced proliferation in this population [6,7,8].

One key regulator of proliferation is ribosome biogenesis (RBG). Replenishment of ribosome pools can occupy as much as 70–80% of a proliferating cell’s energy budget [9]. This high demand is further exemplified by the presence of multiple operons encoding rRNA across the genome and by the ability to directly visualize these sites, the nucleoli, and their activities of rRNA transcription, processing, and ribosome assembly [10]. Initiation of RBG is controlled, in part, by the cellular anabolic effector TORC1 through the ribosomal S6 kinase and is tightly coordinated with the cell cycle through the activity of p53 [11,12]. Cells actively monitor the status of RBG as a measure of cellular health [13], and impairments in rRNA synthesis, processing, or assembly can be visualized as the dissolution of nucleolar structures into the nucleoplasm using antibodies directed against proteins in the RBG complex, a condition known as “nucleolar stress” [14]. Under normal conditions, cells suppress the stability of p53 through the activity of the E3 ubiquitin ligase murine double-minute (MDM2), which targets p53 for degradation [15]. However, under nucleolar stress, select ribosomal proteins, including RPL5, RPL11, and RPL3, are no longer anchored within the nucleolar complex and diffuse into the nucleoplasm to directly interact with MDM2, thereby inhibiting its E3 ubiquitin ligase activity and stabilizing and activating p53. This stabilized p53 protein acts transcriptionally to suppress proliferation and, under conditions when the stressor is unresolved, can initiate cellular apoptosis. Genetic deficits in contributors to RBG such as *TCOF*, *RPL11*, *RPL15*, *RPL26*, and *RPL19* are collectively known as ribosomopathies and cause the p53-mediated depletion of select cell lineages, notably erythrocytes and the cranial neural crest cells that form the bone and cartilage of the face [16,17]. Other ribosomopathies can feature growth stunting and cognitive deficits, and the basis for the heterogeneity of these phenotypes is unknown [18].

Our previous transcriptome analysis of alcohol-exposed early neuroprogenitors identified the suppression of ribosomal protein expression as the most significantly repressed Kyoto Encyclopedia of Genes and Genomes (KEGG) gene cluster at six hours following alcohol challenge [19]. Suppression of this gene cluster also most strongly distinguished the inherent vulnerability of these populations to alcohol-induced apoptosis [20]. Reductions in ribosomal protein expression have also been noted in other models of alcohol exposure, including in the brain [21,22]. We and others have also reported that alcohol exposure activates p53 and p53-mediated signaling, and suppression of its activity prevents the alcohol-mediated apoptosis of cranial neural crest populations [23,24,25]. However, it is unknown whether this represents the induction of nucleolar stress and whether lineages outside of the cranial neural crest are vulnerable to this effect of alcohol. Here, we use an established mouse model of FASD that causes cognitive impairments and reduces brain mass without affecting body growth [26,27,28]; outcomes have similarities with the clinical diagnosis of alcohol-related neurodevelopmental disorder (ARND) [1]. We report that this relatively moderate prenatal alcohol exposure induces nucleolar stress within the developing fetal cortex and in primary NSCs derived from cortical neurospheres; in the primary NSCs, it also causes their p53-mediated apoptotic depletion.

## 2. Materials and Methods

### 2.1. Animals

C57BL/6J mice (Jackson Laboratories, Bar Harbor, ME, USA) were housed in a temperature-controlled room with a 12-h light/dark cycle at the AAALAC-accredited animal facilities of the North Carolina Research Campus. At 8–9 weeks old, females were mated overnight, and the day of vaginal plug was designated embryonic day E0.5.

### 2.2. Alcohol Exposure of Animals

At E0.5, dams were randomly assigned to the control or alcohol group. Alcohol exposed dams (ALC; N = 8) received 3 g of alcohol/kg of body weight (200 proof alcohol, USP grade; Koptec, King of Prussia, PA, USA) via intragastric gavage once daily from E8.5 through E17.5. This established alcohol exposure model causes blood alcohol levels of 211 ± 14 mg/dL at 30 min post-gavage and does not alter maternal and fetal growth or survival but does cause deficits in associative learning in the offspring [26,27,28]. Control dams (CON; N = 8) received intragastric gavage of maltodextrin (Envigo-Teklad, Madison, WI, USA) equivalent to 80% of the alcohol calories because the isocaloric dose causes hyperosmolar harm [28].

### 2.3. Whole Transcriptome Analysis of Fetal Cortex

On E17.5, four hours after the last gavage, the fetuses were harvested from each dam. The fetal brains were isolated, bisected into left and right halves, and flash frozen. For each RNA sample (N = 8 CON litters, N = 8 ALC litters), we pooled the left cortical halves from four littermates and extracted total RNA using TRIzol reagent (Invitrogen, Carlsbad, CA, USA). RNA concentration and purity were measured using the DeNovix DS-11 FX+ spectrophotometer (DeNovix Inc., Wilmington, DE, USA). RNA integrity was assessed using an Agilent 2100 BioAnalyzer (Agilent Technologies, Santa Clara, CA, USA). cDNA libraries were prepared by the UNC High Throughput Sequencing Facility using the KAPA Stranded mRNA-Seq Kit (Roche, Basel, Switzerland). Paired-end (50 bp) whole transcriptome sequencing was performed using the Illumina HiSeq 4000 Platform. Thus, each exposure group (ALC, CON) was comprised of 8 litters, and for each litter, we sequenced a cDNA library from a pool of four fetal brains.

Sequence quality was assessed using the program FASTQC (https://www.bioinformatics.babraham.ac.uk/projects/fastqc/) (accessed 16 June 2019), and reads were aligned to the mm10 reference genome using Bowtie2 [19,29]. Annotated reads were sorted using SAMtools (http://www.htslib.org/doc/samtools.html; accessed 16 June 2019), and featureCounts [30] was used to obtain the per-gene read counts. Gene length influences fold-change estimates that can lead to false-positive calls in gene-set enrichment analysis (GSEA), and this is not corrected by DESeq’s methods of normalization [31]. Therefore, we applied conditional quantile normalization (cqn) as previously described [31] before analyzing differential expression using DESeq2 1.24.0 [32]. We used the Bonferroni method to adjust for false discovery, and gene expression differences were considered significant at an adjusted *p*-value (Padj) ≤ 0.05. Differentially expressed genes were used for GSEA and KEGG pathway annotation via clusterProfiler in R 4.0.2 [33]. Pathways with Padj ≤ 0.05, again using the Bonferroni correction, were considered significant. P53-responsive genes in this dataset were identified by visual inspection in comparison to lists of known p53 targets detailed in KEGG and Ref. [34].

### 2.4. Immunofluorescence Staining and Imaging of Fetal Cortex

CON and ALC fetuses, generated as above, were collected at E14.5 and their brains were fixed in 4% paraformaldehyde and preserved in 30% sucrose/PBS for cryoprotection. Heat-mediated antigen retrieval was performed on coronal sections (20 µm) with citrate antigen retrieval buffer (10 mM sodium citrate, 0.05% Tween-20, pH 6.0) using the steamer method. Fibrillarin-positive nucleoli were detected using rabbit anti-mouse fibrillarin (#ab5821, 1:1500; Abcam, Cambridge, UK) and visualized using Alexa-488 (#A-11094; Invitrogen; Waltham MA, USA)-labeled secondary antibody. P53-positive nuclei were detected using anti-rabbit p53 (1:200, R135, gift of N. Krupenko). Nuclei were visualized using 4′,6-diamidino-2-phenylindole (DAPI) (1:2000; Invitrogen) and mounted with Fluoromount-G (SouthernBiotech, Birmingham, AL, USA). The absence of the primary antibody served as a negative control. Digital images of equivalent cortical regions were captured under uniform exposure. The number and area of fibrillarin+ puncta per DAPI+ nucleus were quantified within the ventricular zone using CellProfiler 4.2.1 [35] (www.cellprofiler.org; accessed 24 September 2021). In brief, the number and area of DAPI+ nuclei and fibrillarin+ nucleoli were defined within 66 × 66 µm regions using the CellProfiler thresholding feature; the punctate nucleoli were further defined using the Enhance feature to remove background and the MaskImage feature to remove the overlapping DAPI signal. The number, area, and intensity of fibrillarin+ nucleoli were calculated for individual nuclei, counting 37–53 nuclei per cortex. The number of p53+ nuclei was quantified using ImageJ (v.54.1) [36] and sampling three sections per cortex. One fetus per litter was sampled from 4 CON and 5 ALC litters.

### 2.5. Primary Neural Stem Cell (NSC) Cultures

Primary NSC cultures were prepared exactly as described in [8,37,38]. Dorsal neuroepithelia were dissected from naïve E14.5 mouse precursor neocortices, avoiding the hippocampus, striatum, and meningeal tissue, and single cell suspensions of these primary cortical NSCs were prepared using manual trituration under 0.5% trypsin and were maintained as nonadherent neurospheres on uncoated 25 mL flasks (Corning, Inc., Corning, NY, USA,) using DMEM/F12 (#11039-021; Gibco, Grand Island, NY, USA) containing 20 µg/mL epithelial growth factor (EGF, #AF-100-15; Peprotech, Cranbury, NJ, USA), 200 µg/mL basic fibroblast growth factor (bFGF, #PHG-0263; Gibco), 100-18b insulin-transferrin-selenium (ITS-X,100X) (#51500-056; Gibco), 5 µg/µL heparin (#15077-019; Sigma, Louis, MO, USA), 1 µg/mL leukemia inhibitory factor (LIF, #L-200; Alomone Labs, Jerusalem, Israel), and 50 µM progesterone (#P7556; Sigma) [8,37,38]. Fresh culture media was added every 2–3 days, and the cells were dissociated using StemPro Accutase (#A1110501; Gibco) every 4 days for passage. Monolayer cultures were prepared from the above neurospheres using mechanical dissociation with Accutase and plated at a density of 2 × 10^5^ cells/mL on coverslips coated with 100 µg/mL poly-L-ornithine (#P4957; Sigma) and fibronectin (#F1141; Sigma) and housed in 6-well plates with the above culture medium.

For the alcohol exposures of NSCs, monolayers at 70–80% confluence were either left untreated (CON) or were treated with 80 mM ethanol (ALC; 200 proof, USP grade as above) unless indicated otherwise and were harvested at experimentally determined times thereafter; we find that <10 mM ethanol remains in the media at 3 h post-addition [23]. Studies were performed using NSCs derived from the fetuses of at least three different dams, and each experimental replicate consisted of the pooled cortices from individual litters. For most studies, unless indicated otherwise, the data are the means of three independent experiments, each with at least three samples per group.

### 2.6. NSC Transfection Studies

Primary NSCs were transiently transfected at 80% confluence with plasmid overexpression vectors and Lipo-3000 (#L3000001; Invitrogen, Waltham, MA, USA) according to the manufacturer’s instructions. The plasmids were MDM2 PCDNA3 WT (#16233; Addgene, Watertown, MA, USA) and XE150 DN P53-PCS2P+ (#17033; Addgene). Cells were allowed to recover for 6 h and then were treated with 80 mM ethanol and analyzed at experimentally-determined times thereafter for apoptosis and p53 and MDM2 protein content, as described below.

### 2.7. Immunofluorescent Staining and Imaging of NSCs

Cells were fixed with 4% paraformaldehyde, permeabilized with 0.1% Triton X-100, and blocked using 1% bovine serum albumin and 1% heat-inactivated goat serum in phosphate-buffered saline (PBS). Primary antibodies were directed against fibrillarin (#ab5821; 1:500), nucleolin (#ab22758, 1:250), UBF (#ab61205, 1:250; all from Abcam) or p53 (1:200, R135, gift of N. Krupenko), followed by secondary antibodies conjugated to Alexa-488 (1:1000, Invitrogen); nuclei were visualized using DAPI. Digital images were made under uniform exposure, capturing three images per sample, and the number of nucleoli per nucleus and percentage of fibrillarin+, nucleolin+, or UBF+ nucleolar area per DAPI+ nucleus were calculated using ImageJ [36].

### 2.8. Quantification of NSC Proliferation

Monolayers of primary NSCs were exposed to 80 mM ethanol, and 12 h later, they were changed into fresh media containing 10 µM 5-ethynyl-2′-deoxyuridine (EdU; Invitrogen). The cells were fixed 1 h thereafter, permeabilized as above, and the EdU+ cells enumerated using Click chemistry with Alexa-488-azide (#A-11094; Invitrogen) diluted in 100 mM CuSO_4_/100 mM ascorbic acid in Tris-buffered saline. Nuclei were visualized using DAPI. The omission of Alexa-azide served as a negative control. Images were captured under uniform exposure, capturing three images per sample.

### 2.9. Assessment of NSC Apoptosis

Monolayer cultures of primary NSCs were exposed to 80 mM ethanol for experimentally determined times, fixed in 4% paraformaldehyde/PBS, permeabilized in 0.2% TritonX-100, and apoptosis was detected using terminal deoxynucleotidyl transferase dUTP nick end labeling (TUNEL) staining (DeadEnd Fluorometric TUNEL System; Promega, Madison, WI, USA). Nuclei were visualized using DAPI. Images were taken under uniform exposure, capturing three images per sample.

### 2.10. Western Blot Analysis

Primary NSCs were lysed in NonidetP-40 (NP-40) lysis buffer (50 mM Tris-HCl, pH 7.5, 150 mM NaCl, 0.5% NP-40, 50 mM NaF, 1 mM NaVO3, 1 mM dithiothreitol, and 1 mM phenylmethylsulfonyl fluoride). Total protein was separated on a 10% SDS-PAGE reducing gel, and resolved proteins were transferred onto a PVDF membrane using the Trans-Blot Turbo Transfer System (#1704150, Bio-Rad, Hercules, CA, USA). Proteins of interest were quantified using primary antibodies directed against MDM2 (1:1000, ab259265; Abcam), p53 (#ab26, 1:500; Abcam), and GAPDH (1:2000, G8795; Sigma), followed by goat anti-secondary antibodies coupled to rabbit (1:5000, #4010-05) and mouse (1:5000, #1030-05, both Southern Biotech). The signal was imaged by chemiluminescence using the Radiance Q detection system (Azure Biosystems, Dublin, CA, USA).

### 2.11. Quantitative PCR (qPCR)

RNAs from NSCs were exposed to 80 mM ethanol for 0 to 12 h, and total RNA was isolated with Trizol reagent (Invitrogen). Total RNA (1 µg) was reverse transcribed with 500 µg/mL random primer (C1181), 5X ImProm II reaction buffer (M289A), ImProm II (M314A), 25 mM MgCl2 (A351H), 10 mM dNTP (U1511), 20U RNAsin (N2511, all from Promega, Madison, WI), and RNAse-free water. qPCR was performed using SYBR Select Master Mix (ABI, #4472913) and the Real-Time PCR system (Bio-Rad CFX96). Primers directed against the *M. musculus* internal transcribed spacer-1 (ITS-1) of 47S pre-rRNA (forward: 5′-CCGGCTTGCCCGATTT-3′, reverse: 5′-GCCAGCAGGAACGAAACG-3′) and β2 microglobulin (forward: 5′-TTCACCCCCACTGAGACTGAT-3′; reverse: 5′-GTCTTGGGCTCGGCCATA-3′) [39] were from IDT (Coralville, IA, USA). We used the 2^−ΔΔCT^ method to analyze relative gene expression and normalized to β2 microglobulin used as a control.

### 2.12. Statistical Analysis

All data were checked for normality (Shapiro–Wilk test) and unequal variance (Spearman’s test for heteroscedasticity). Unpaired two-tailed *t*-tests were used when two groups were compared, and an ANOVA was used for more than two groups. Associations between gene expression and fetal brain weight were assessed using Pearson’s correlation. Analyses were performed using GraphPad Prism version 9.3.1 (San Diego, CA, USA) or R Studio running R version 4.0.2. Results are expressed as mean ± SD. The significance was set at *p* < 0.05.

## 3. Results

### 3.1. Alcohol Exposure Suppresses Pathways Associated with Ribosomes and Oxidative Phosphorylation in the E17.5 Fetal Brain

**Figure 1:**
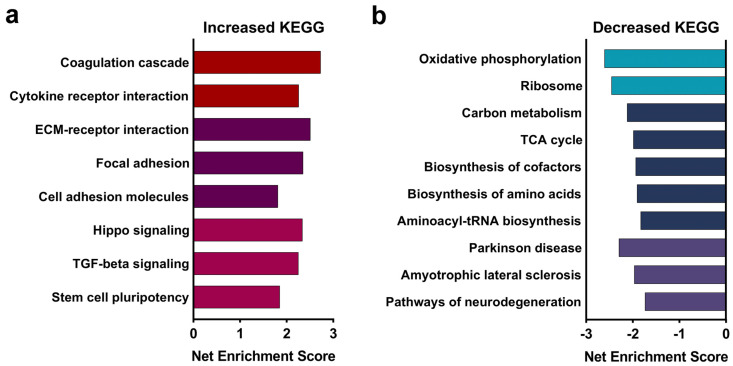
**Dysregulated Kyoto Encyclopedia of Genes and Genomes (KEGG) pathways representative of differentially expressed genes in the alcohol-exposed E17.5 brain.** Gene-Set Enrichment Analysis identified KEGG pathways that were dysregulated by prenatal alcohol exposure. (**a**) Gene clusters containing genes having increased expression in alcohol-exposed fetal brains relative to controls involved in inflammation, cell–cell interactions, and stem cell differentiation. (**b**) KEGG clusters having genes with decreased expression were largely represented by clusters involved in energy generation, ribosome biogenesis, and oxidative phosphorylation. N = 8 litters per group.

The whole transcriptome sequencing of the E17.5 fetal brain identified 13,104 transcripts that could be mapped to the *mm10* reference genome. Following adjustment for gene length bias (Figure S1), we identified 2848 genes that were differentially expressed (Padj < 0.05) in the ALC fetal brain relative to controls; of these, 49.6% (1414 genes) were down-regulated and 50.4% (1435 genes) were up-regulated (Table S1). KEGG analysis of the alcohol-responsive genes identified 27 gene clusters whose members had enriched abundance in response to ALC (Table S2). Many of these clusters held numerous redundant genes and could be reduced to gene clusters involved in inflammation, cell–cell interactions, and stem cell differentiation (Figure 1a). For inflammation and cellular stress, the greatest enrichment scores included cytokine–cytokine receptor interactions, advanced glycation endproducts (AGE-RAGE) signaling, and complement/coagulation cascades, and included multiple cytokines and their receptors (*Csf1*, *Csf1r*, *Cxcl12*, *Il17ra*, *Il6ra*, and *Inha*), genes related to the complement cascade (*A2m*, *C3*, *Cfb*, and *Cfh*), and blood clotting (*Fga*, *Fgb*, *Fgg*, and *Vwf*) (Table 1). Another set of ALC-enriched clusters involved cell–matrix interactions such as extracellular matrix (ECM)–receptor interactions, focal adhesion, protein digestion and absorption, and proteoglycans in cancer and included 22 different collagens, as well as fibronectin, integrins, and laminins. A third set of KEGG clusters focused on stem cell growth and included genes involved in Hippo signaling (*Amot*, *Lats2*, *Mob1b*, *Tead1*, *Wwtr1*, and *Yap1*), TGF-beta signaling (*Acvr1b*, *Acvr1c*, *Acvr2a*, *Bmp5*, *Bmp6*, *Bmp7*, *Bmpr2*, *Smad3*, *Smad6*, *Smad7*, *Tgfb3*, and *Tgfbr2*), and stem cell pluripotency (*Rest*, *Zfhx3*, and multiple components of Wnt and BMP/Activin signaling).

Similarly, 22 KEGG clusters had reduced enrichment in the ALC fetal brain (Table S3). Many of these involved diseases of neurodegeneration, including amyotrophic lateral sclerosis and Parkinson, Huntington, and prion diseases. A closer review of these gene lists revealed that they largely focused on two important processes that are tightly coordinated within the cell, energy generation and ribosome biogenesis (Figure 1b). With respect to energy generation, this included 16 genes that contribute to the TCA cycle (*Aco2*, *Fh1*, *Idh3a*, *Idh3b*, *Mdh1*, *Mdh2*, *Sdha*, *Sdhb*, *Sdhc*, *Sdhd*, and *Pdha1*) and 63 genes across all five complexes of oxidative phosphorylation, including NADH dehydrogenase (26 genes), succinate dehydrogenase (3 genes), ubiquinol-cytochrome C reductase (6 genes), cytochrome C oxidase (11 genes), and the ATP synthase (14 genes). Additional affected pathways included fatty acid oxidation (*Echs1* and *Hadha*), glycolysis (*Pfkl*, *Pgam1*, *Pgk1*, *Ldhb*, and *Tpi1*), and organic acid metabolism (*Hibch*, *Mcee*, *Pccb*, *Pcx*, *Sucla2*, and *Suclg1*). This was complemented by the suppression of gene clusters involving ribosome biogenesis, including genes encoding 25 mitochondrial and 49 nuclear ribosome proteins, 76 involved in rRNA processing and assembly, and 17 aminoacyl-tRNAs (Table S4). Also captured within the neurodegenerative KEGG clusters were 21 genes encoding proteosome components and 6 components of RNA polymerase II (*Polr2d*, *Polr2e*, *Polr2f*, *Polr2g*, *Polr2h*, and *Polr2i*). The median expression of cellular ribosomal proteins in ALC E17.5 fetal cortex was 92% (range 70–99%) of CON values and 91% (range 78–96%) for mitochondrial ribosomal proteins; fold changes for all RBG-related genes are presented in Table S4.

### 3.2. Alcohol Reduces Nucleoli Numbers in Fetal Cortex

**Figure 2:**
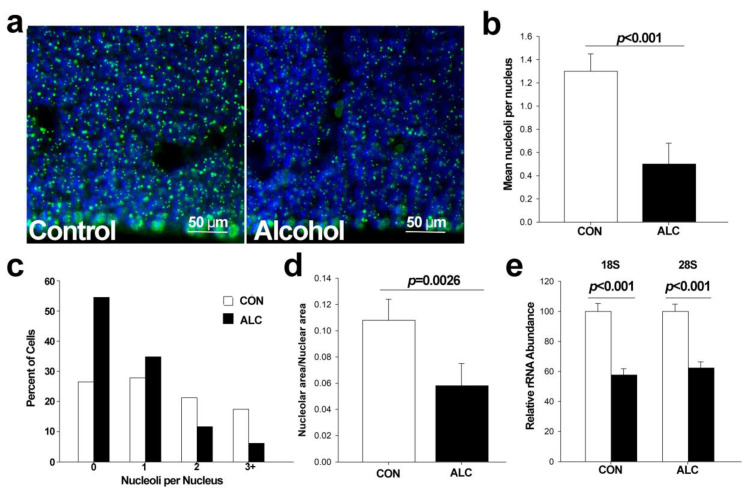
**Alcohol exposure reduces nucleoli numbers in the E14.5 fetal cortex.** (**a**) Immunostaining of fibrillarin (green) in CON (control) and ALC (alcohol-exposed) E14.5 fetal cortex. Nuclei were identified by DAPI (blue). The scale bar represents 50 µm. (**b**) Alcohol exposure (ALC) reduces the mean number of nucleoli in the E14.5 fetal cortex. (**c**) Alcohol exposure reduces the number of nuclei having 2–3 nuclei and increases the number having none. (**d**) Alcohol exposure reduces the mean nucleolar area per nucleus in the E14.5 fetal cortex. (**b**–**d**) represent values for four CON and five ALC brains. (**e**) The 18S and 28S rRNA content is reduced in the ALC fetal cortex (n = 8 per group). Values are mean ± SD. Statistical comparisons using an unpaired two-tailed *t*-test and an ANOVA for more than two groups.

The reduced abundance of genes involving rRNA synthesis, rRNA processing and assembly, ribosomal proteins, and tRNA synthases led us to consider whether alcohol was causing nucleolar stress, as previously suggested for alcohol-exposed early neuroprogenitors [19]. Under nucleolar stress, the components of ribosome biogenesis lose their anchorage and disperse into the nucleoplasm [14]. Because ribosome biogenesis is necessary to replenish the ribosomal content following cell division, we focused on the ventricular zone of the E14.5 cortex around the period of peak NSC proliferation [40]. We visualized nucleolar structures using immunostaining for the box C/D snoRNP 2′-O-methyltransferase fibrillarin, which contributes to pre-rRNA processing within the nucleolar dense fibrillar center [41]. Alcohol exposure was associated with a reduced fibrillarin signal within the nuclei of the cortex (Figure 2a), and this reflected a reduction in the mean number of fibrillarin+ nucleoli per nucleus (CON 1.30 ± 0.15, ALC 0.58 ± 0.18; *p* = 0.00035; Figure 2b). Although there appeared to be more nuclei containing no nucleoli and fewer having two or three nucleoli per nucleus (Figure 2c), those differences were not significant. Nonetheless, quantification confirmed the reduction in the fractional nuclear area that was fibrillarin+ (CON 0.108 ± 0.016; ALC 0.058 ± 0.017; *p* = 0.0026; Figure 2d). These losses were accompanied by a reduction in the cortical content of both 18S and 28S rRNA. When normalized to DNA content, the alcohol-exposed brains had 57.5% less 18S and 62.4% less 28S rRNA content, consistent with a nucleolar stress phenotype (Figure 2e). These changes in nucleolar structure and rRNA content were consistent with alcohol exposure causing nucleolar stress within cortical NSCs.

### 3.3. Alcohol Induces Nucleolar Stress in Cortical Neural Stem Cells (NSCs)

**Figure 3:**
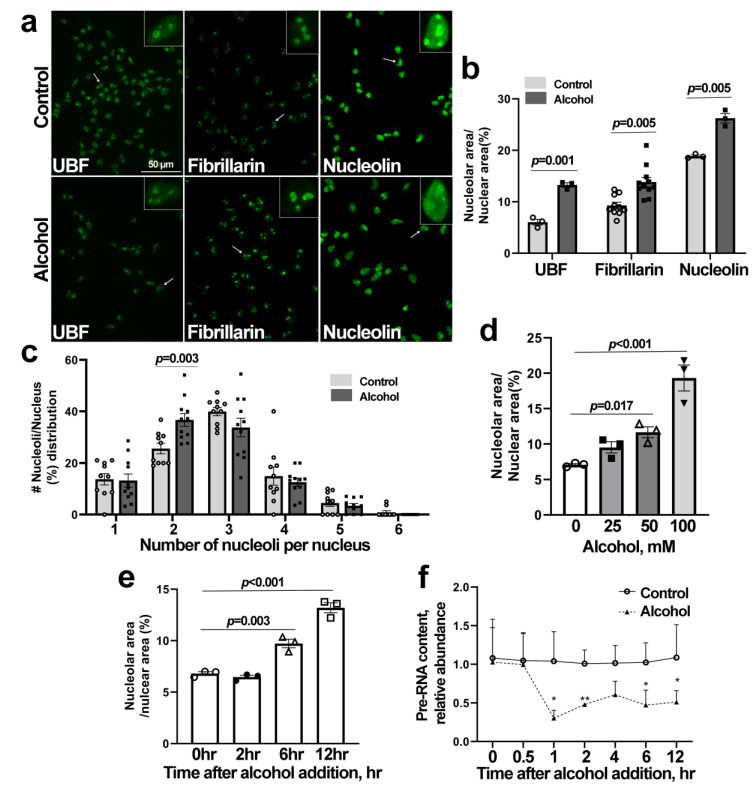
**Alcohol exposure induces nucleolus stress in primary NSCs derived from the E14.5 cortex.** (**a**) Immunostaining for UBF, fibrillarin, and nucleolin proteins (green) in primary NSCs at 6 h after 80 mM alcohol exposure. Inserts present representative nuclei that are enlarged to visualize nucleolar structure; arrows indicate the selected cells. The scale bar indicates 50 µm. (**b**) Alcohol exposure increases the mean nucleolar area occupied by UBF (n = 3 per group), fibrillarin (n = 10 per group), and nucleolin (n = 3 per group) in primary NSCs, suggesting nucleolar diffusion. (**c**) Alcohol alters the nucleoli per cell distribution in primary NSCs as assessed using fibrillarin, and more nuclei had just two nuclei (n = 10 per group). (**d**) Alcohol increases nucleolar area, assessed using UBF, in a dose-dependent manner at 6 h post-exposure (n = 3 per group). (**e**) Alcohol increases nucleolar area between 2 h and 6 h post-exposure, as assessed using UBF (n = 3 per group). (**f**) Alcohol reduces rRNA synthesis within 1 h of exposure, as shown by quantitation of the pre-rRNA splice sequence ITS-1. * *p* < 0.05, ** *p* < 0.01 (n = 3 per group). Data represent the mean ± SD; symbols in (**b**–**e**) indicate experimental replicates. Statistical comparisons using an unpaired two-tailed *t*-test and an ANOVA for more than two groups.

We investigated the mechanism underlying this alcohol-induced nucleolar stress using primary NSCs derived from the E14.5 cortex. At 6 h following alcohol exposure, the nucleoli of NSCs had an elongated appearance and were more disorganized and diffuse compared with those of control cells, as assessed using antibodies directed against three distinct events of RBG: the upstream binding factor (UBF), which contributes to rRNA transcription; fibrillarin, which contributes to pre-rRNA processing; and nucleolin, which contributes to ribosome assembly (Figure 3a and Figure S2). This diffuse structure was confirmed by quantification of nucleolar area per nucleus, which was increased in the alcohol-exposed NSCs as shown for UBF (CON 6.02 ± 0.57%, ALC 13.20 ± 0.49%, *p* = 0.005), fibrillarin (CON 9.28 ± 1.86%, ALC 13.82 ± 2.90%, *p* = 0.005), and nucleolin (CON 18.86 ± 0.23, ALC 26.23 ± 0.91, *p* = 0.005; Figure 3b). Although the mean number of nucleoli per nucleus did not differ (CON 2.92 ± 0.66, ALC 2.56 ± 0.24, *p* = 0.104), more alcohol-exposed cells had just two nucleoli (CON 25.60 ± 6.31%, ALC 36.68 ± 8.3%, *p* = 0.003; Figure 3c). This response to alcohol was dose-dependent, and by 6 h post-exposure, concentrations as low as 25 mM caused a rise in nucleolar area that was significant at 50 mM (Figure 3d). This increased nucleolar dissolution occurred between 2 h and 6 h of alcohol exposure and persisted for at least 12 h post-exposure (Figure 3e). Nucleolar dissolution can be preceded by a loss of rRNA synthesis, and these NSCs had a 70% decline in pre-rRNA content within 0.5 to 1 h following alcohol exposure, as quantified using qPCR against the ITS-1 sequence within 47S pre-rRNA (Figure 3f). Again, this decline persisted for at least 12 h following the alcohol exposure. Taken together, this phenotype was consistent with the presence of nucleolar stress in these alcohol-exposed NSCs.

### 3.4. Alcohol-Induced Nucleolar Stress in NSCs Is Accompanied by Stabilization of Nuclear p53 and Cell Cycle Arrest

**Figure 4:**
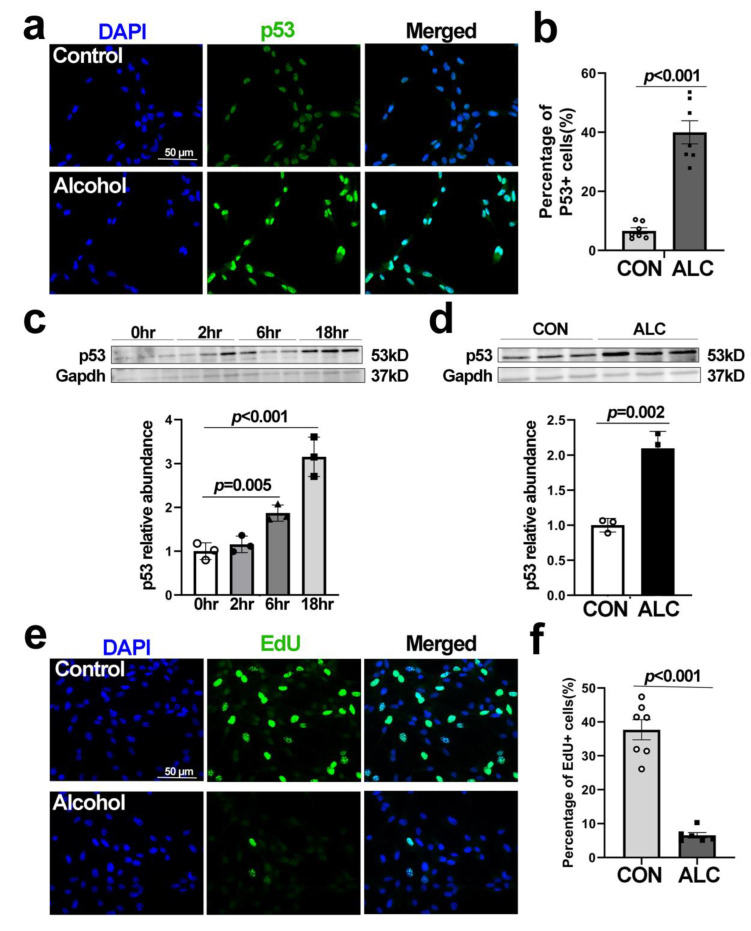
**Alcohol exposure stabilizes the p53 protein and induces cell cycle arrest in primary NSCs derived from the E14.5 cortex.** (**a**) Immunostaining of P53 (green) in primary NSCs at 12 h following alcohol exposure. Nuclei were identified by DAPI (blue). (**b**) Alcohol increases the percentage of P53-positive nuclei in primary NSCs (n = 7 per group). (**c**) Time course using western blot analysis confirms the elevated p53 protein content, normalized to Gapdh, in primary NSCs at 6 h and 18 h following alcohol exposure (n = 3 per group). (**d**) p53 protein content is also elevated at 12 h following alcohol exposure (n = 3 per group). (**e**) EdU incorporation (green) in primary NSCs, labeled for 1 h after 12 h of alcohol exposure. Nuclei were identified by DAPI (blue). (**f**) Alcohol reduced the percentage of EdU-positive cells in primary NSCs (CON n = 7, ALC n = 6). Data represent the mean ± SD, and symbols in (**b**–**d**,**f**) indicate experimental replicates. The scale bar in (**a**,**e**) represent 50 µm. Statistical comparisons using an unpaired two-tailed *t*-test and an ANOVA for more than two groups.

Defects in ribosome biogenesis and nucleolar stress can activate MDM2-p53-mediated cell cycle G1 phase arrest and cell stress responses. Immunostaining for p53 showed that alcohol led to the stabilization and nuclear accumulation of p53 in NSCs (Figure 4a), and at 12 h post-exposure, this represented a 7.31-fold enrichment in the percentage of p53+ NSC nuclei (CON 6.64 ± 1.02%, ALC 39.97 ± 3.91%, *p* < 0.001; Figure 4b). This represented a near-doubling of p53 content by 6 h after alcohol exposure (relative abundance CON 1.00 ± 0.19, ALC 1.87 ± 0.18, *p* = 0.005), and this elevation persisted through 12 h (CON 1.00 ± 0.09, ALC 2.10 ± 0.24, *p* = 0.002) and 18 h post-exposure (CON 1.00 ± 0.19, ALC 3.15 ± 0.45, *p* < 0.001; Figure 4c,d). This was accompanied by an 83% reduction in the percentage of proliferating NSCs (Figure 4e), as quantified by EdU incorporation (CON 37.68 ± 2.97%, ALC 6.54 ± 0.82%, *p* < 0.001; Figure 4f), indicating that alcohol also induced their growth arrest.

### 3.5. Alcohol Induces p53-Dependent Apoptosis in NSCs That Is Abrogated by MDM2

**Figure 5:**
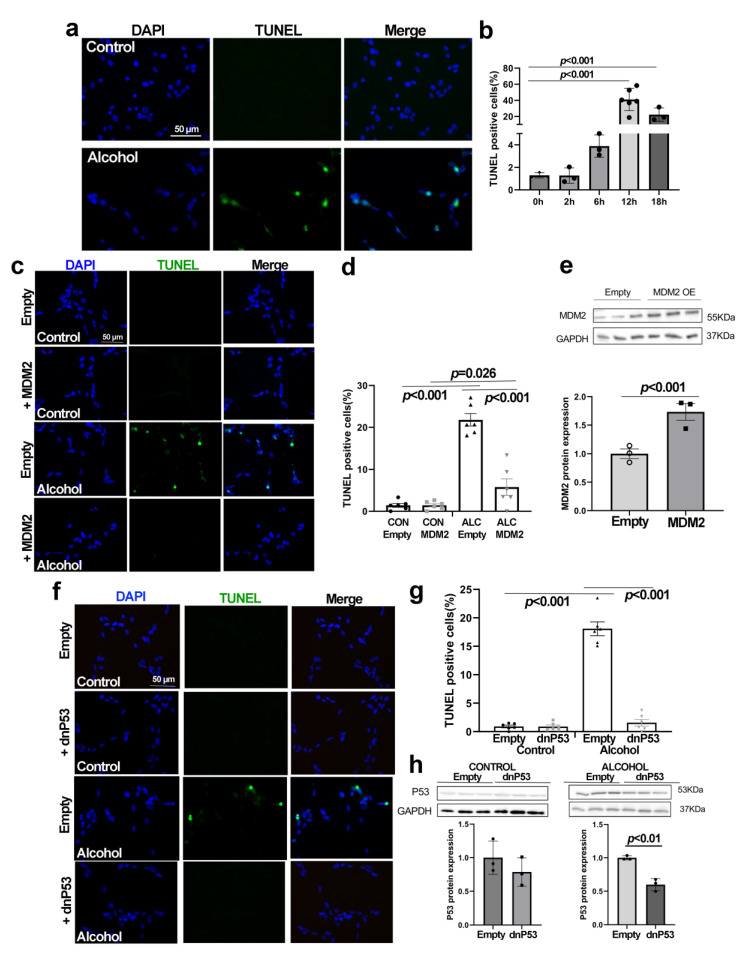
**Alcohol exposure induces P53-dependent apoptosis that is abrogated by overexpression of MDM2 or dominant-negative P53 in primary NSCs derived from the E14.5 cortex.** (**a**) The TUNEL assay detects apoptotic DNA fragmentation (green) in the nuclei of alcohol-exposed primary NSCs at 12 h after exposure. Nuclei were identified by DAPI. (**b**) Timecourse analysis reveals that the alcohol-mediated apoptosis of primary NSCs is increased at 12 h and 18 h after exposure (n = 3–6 per group). (**c**) The TUNEL assay detects apoptotic DNA fragmentation (green) in the nuclei of primary NSCs transfected with MDM2. Nuclei were identified by DAPI (blue). (**d**) Transfection with MDM2 prevents apoptosis in alcohol-exposed (ALC) primary NSCs but not in controls (CON), as detected using the TUNEL assay (n = 6 per group). (**e**) Western blot analysis confirms elevated MDM2 protein in transfected NSCs (n = 3 per group). (**f**) Apoptosis in primary NSCs following alcohol exposure and/or transfection with dominant-negative p53, as detected using TUNEL for DNA fragmentation (green). Nuclei were identified by DAPI. (**g**) Transfection with dominant-negative p53 prevents apoptosis in alcohol-exposed primary NSCs but not in controls, as detected using the TUNEL assay (n = 6 per group). (**h**) Western blot analysis for total p53 protein in primary NSCs in alcohol’s absence (left panel) or 12 h following its addition (right panel; n = 3 per group). Data represent the mean ± SD, and symbols in (**b**,**d**,**e**,**g**,**h**) represent experimental replicates. Statistical comparisons were made using an unpaired two-tailed *t*-test when two groups were compared and an ANOVA for more than two groups. The scale bar in (**a**,**c**,**f**) is 50 µm.

Under nucleolar stress, the stabilized p53 can initiate apoptosis in addition to cell cycle arrest. At 12 h after alcohol exposure, the percentage of apoptotic NSCs increased 33-fold as assessed through detection of nuclear DNA fragmentation (0 h, 1.29 ± 0.13%; 12 h, 42.86 ± 4.97%, *p* < 0.001), and this elevation persisted through at least 18 h post-exposure (Figure 5a,b). Transient transfection of NSCs with MDM2, which under nucleolar stress is displaced from p53 through interactions with select ribosomal proteins, did not affect cell survival in control NSCs but attenuated the alcohol-induced apoptosis (ALC 21.80 ± 1.50%; ALC + MDM2 5.78 ± 1.97%, *p* < 0.001; Figure 5c,d). Western blot analysis confirmed the increased MDM2 content in transfected cells (Figure 5e). We further affirmed the p53-dependence of this apoptosis as transient transfection with a dominant-negative p53 construct did not further affect apoptosis in control NSCs and suppressed their alcohol-induced apoptosis (ALC 18.09 ± 1.22%, ALC + dnP53 1.56 ± 0.59%, *p* < 0.001; Figure 5f,g). The level of total p53 decreased in the transfected alcohol-exposed NSCs (*p* = 0.01; Figure 5h) and trended lower in transfected controls, consistent with the ability of dnP53 to destabilize cellular p53 content [42].

**Figure 6:**
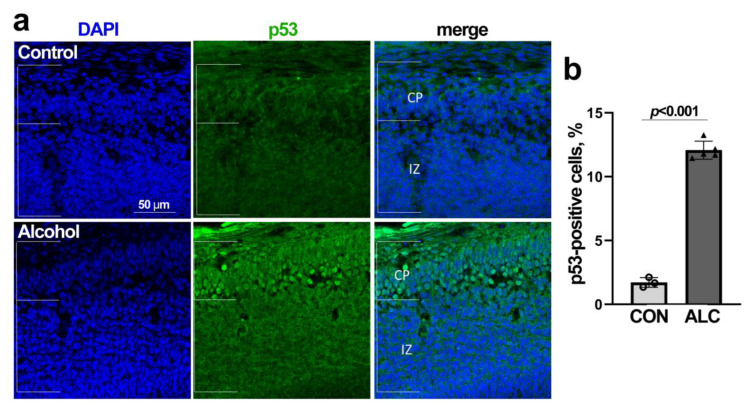
**Alcohol exposure stabilizes nuclear p53 in the E14.5 fetal cortex.** (**a**) Immunostaining for p53 (green) in control (CON) and alcohol-exposed (ALC) E14.5 fetal cortex. Nuclei were identified by DAPI (blue). The scale bar represents 50 µm. White bars indicate the boundaries for the cortical plate (CP) and interventricular zone (IZ). (**b**) The percentage of p53-positive cells is significantly higher in the ALC fetal cortex. Data represent the mean ± SD, and symbols represent experimental replicates with three CON and five ALC fetal brains. Statistical comparisons were made using an unpaired two-tailed *t*-test.

Given that nucleolar stress led to the stabilization of nuclear p53 in alcohol-exposed primary NSCs, we asked if the alcohol-induced nucleolar stress in the fetal cortex was accompanied by a similar activation of p53. Immunostaining of the E14.5 fetal cortex revealed a 7.0-fold increase in the percentage of p53-positive nuclei within the ALC cortex, and these were enriched within the cortical plate (CON 1.72 ± 0.38%, ALC 12.08 ± 0.70%, Figure 6a,b). This represented an increase in p53 activity, as affirmed by our prior whole transcriptome analysis. Although p53 signaling did not emerge from that KEGG analysis (Table 1), direct inspection of the alcohol-responsive gene list (Table S1) revealed the altered abundance of 53 genes known to be directly regulated by p53 (Table S5; 34). These included *Afp*, *Apc*, *Bax*, *Bcl2*, *Csf1*, *Gadd45gip1*, *Hic1*, *Mdm1*, *Mdm4*, *Mpzl2*, *Rgcc*, and *Tgm2*, with fold changes (ALC/CON) ranging from 83% lower to 651% higher. Thus, as with the primary NSCs, the alcohol-induced nucleolar stress in the fetal cortex is accompanied by the stabilization and activation of nuclear p53.

### 3.6. Alcohol-Induced Suppression of Ribosomal Proteins and Oxidative Phosphorylation Components Negatively Correlates with Fetal Brain Weight

**Figure 7:**
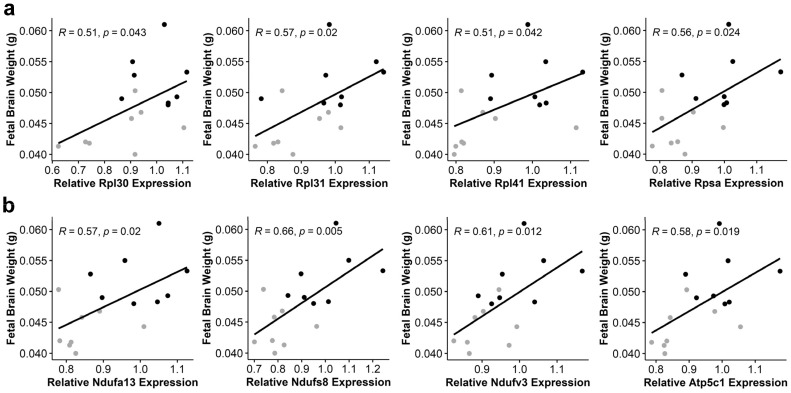
**Expression of ribosomal proteins and components of oxidative phosphorylation correlates with E17.5 fetal brain weight.** (**a**) The relative abundance of transcripts encoding the ribosomal proteins *Rpl30*, *Rpl31*, *Rpl41*, and *Rpsa* positively correlates with fetal brain wet weight at E17.5. (**b**) The relative abundance of transcripts encoding components of oxidative phosphorylation, including *Ndufa13*, *Nudfs8*, *Nduvf3* in Complex 1, and *ATP5c1* in the ATP synthase, positively correlates with fetal brain weight at E17.5. N = 8 litters per group; fetal brain weights are the mean of the four brains sampled per litter and are the same four brains that were pooled for the RNA quantification using RNA-seq. The data were analyzed using Pearson correlation. Grey dots, ALC; black dots, CON.

Suppression of ribosome biosynthesis and oxidative phosphorylation can initiate nucleolar stress, and these KEGG pathways were among the most reduced in the alcohol-exposed fetal brain. To gain insight into their potential contributions to the brain stunting that partly typifies prenatal alcohol exposure, we performed Pearson correlation analysis against the expression of components in these pathways against the wet weights of those E17.5 brains at collection. We identified significant positive associations between fetal brain weight and the expression of multiple ribosomal protein genes (*Rpl30*, *Rpl31*, *Rpl41*, and *Rpsa*) (Figure 7a). Positive correlations were also identified for multiple components of oxidative phosphorylation, including proteins within complex I (*Ndufa13*, *Ndufb2*, *Ndufs4*, *Ndufs8*, and *Ndufv3*), complex III (*Uqcrq*), complex V (*Atp5c1*, *Atp5k1*, and *Atp6v0e2*), and the generation of inorganic phosphate (*Ppa2*) (Figure 7b, Table 2). Although these correlations were independent of exposure status, they were consistent with the lower weights and expression levels in the alcohol-exposed brains.

## 4. Discussion

The most important finding from this study is that alcohol exposure induced nucleolar stress and activated nuclear p53 in both the intact fetal cortex and primary NSCs, and in the primary NSCs, this was accompanied by the activation of P53/MDM2-mediated apoptosis. Transcriptome analysis revealed that PAE markedly inhibited the expression of genes involved in RBG, including *Rpl30*, *Rpl31*, *Rpl41*, and *Rpsa*, and this suppression is a likely driver of the nucleolar stress. This nucleolar stress was exemplified by an increased nucleoli area per nucleus that was indicative of nucleolar dissolution and a reduction in nucleoli per nucleus that suggested fewer sites of RBG in response to alcohol. This was accompanied by an inhibition of cell proliferation and the initiation of apoptosis in NSCs that was dependent upon p53/MDM2 signaling. To our knowledge, this is the first demonstration that alcohol exposure causes nucleolar stress within any neuronal population. It also offers mechanistic insight into alcohol’s known ability to stabilize and activate p53 in neuronal cells [43]. These findings emphasize the potential of untargeted analysis to generate novel hypotheses regarding the mechanisms that mediate alcohol’s toxicity.

The underlying mechanism by which alcohol exposure induces nucleolar stress in primary NSCs—or in any model—is incompletely understood. Impairment of RBG is the primary mediator of nucleolar stress [14], and accumulating evidence suggests that alcohol can inhibit RBG. Our prior transcriptome analysis of early neuroprogenitors (late neurulation stage) revealed that the most extensive and highly significant gene cluster that distinguished sensitive and resistant *G. gallus* strains with respect to alcohol-induced apoptosis is the ribosomal protein gene cluster, such that expression of these genes was 30–50% lower in the vulnerable strain [20]. Pharmacologically relevant alcohol treatment of these same neuroprogenitors reduced the expression of nearly all nuclear and mitochondrial ribosomal proteins, as well as multiple genes contributing to RBG, including *TCOF* [23] (Flentke et al., in revision). Partial knockdown of ribosomal proteins known to induce nucleolar stress, including *zrpl11*, *zrpl5a*, and *zrps3a*, heightens the vulnerability of zebrafish embryos to craniofacial deficits and apoptosis in response to alcohol exposure [19], as does haploinsufficiency of *mdm2*, *nolc1*, or inhibition of RNA polymerase-I (Flentke et al., in revision). Nucleolar stress can be characterized by a loss of nucleoli as these transcriptional sites are silenced and also by an expanded nucleolar area that represents the dissolution of RBG components into the nucleoplasm, as observed after treatment with the classic inducer of nucleolar stress, the RNA polymerase-I inhibitor actinomycin D [44]. This is the first report of similar morphological changes in nucleolar structure in response to alcohol exposure, changes that are consistent with both nucleolar stress and the accompanying reduction in RBG-related genes.

Alcohol’s induction of nucleolar stress offers insight into its known ability to induce p53 in diverse neural lineages, including those derived from the cortex [45], somatosensory cortex [25], dorsal root ganglia [46], and immortalized NSCs [43,47], as well as cranial neural crest cells [23,24,48]. This is accompanied by the up-regulation of p53-dependent pathways [21,25]. The forced expression of p53 promotes the cessation of mitosis in NSCs and enhances their vulnerability to alcohol-induced apoptosis, whereas its knockdown protects against both [45]. Surprisingly little is known regarding the potential upstream mechanisms responsible for p53’s induction by alcohol, and prior work has implicated alcohol-driven phosphoinositide-mediated calcium transients [23] and p38 MAPK/p53 signaling via suppression of upstream miR-135a [49]. Our demonstration of suppressed ribosome biogenesis and the rapid onset of nucleolar stress in these NSCs offers novel mechanistic insight into their p53/MDM2-mediated cell cycle exit and apoptosis.

Reductions in brain mass and head circumference serve as indicators of behavioral dysfunction and can be characteristic features of PAE [1], and thus the brain weight reductions observed here are consistent with clinical findings and other rodent models of PAE [50,51,52]. These size reductions reflect, in part, a reduction in neuronal cellularity attributed to both increased neuronal death and a delayed progression through and increased exit from the cell cycle [8,53], processes consistent with the requirement for ribosome biogenesis in the proliferation and self-renewal of NSCs [17,54]. The nucleolar dissemination and ribosomal expression losses reported here are consistent with such mechanisms and with the cognitive impairments and brain reductions associated with several ribosomopathies, including Treacher Collins, X-linked ribosomopathy, primary microcephaly (MCPH), and some forms of Diamond Blackfan anemia [16,17,55]. Impairments in RBG are also early features of neurodegenerative disorders, including Alzheimer’s and Parkinson’s diseases [56,57]. Whereas the focus here is on neuronal proliferation and survival, it is important to note that ribosome biosynthesis is also critical for the formation and maintenance of dendritic growth, which has a high requirement for protein synthesis, and reductions in ribosomal proteins—including RPS6, RPS14, and RPL4, which are also reduced in our PAE model—lead to smaller dendritic trees and reduced neuronal connectivity [58], deficits also observed in preclinical models of PAE [59,60]. Although the suppression of ribosome biogenesis reported here likely recovers after the alcohol exposure ends, as seen in the cranial neural crest (Flentke et al., in revision), this may still contribute to lasting phenotypes such as microcephaly and cognitive impairment, as revealed in the positive correlations between ribosomal protein expression and fetal brain weight, as well as the cognitive deficits that characterize this PAE model [26,27]. Taken together, these findings suggest that nucleolar stress and ribosome dysbiogenesis are novel mechanistic contributors to the reduced brain weight and cognitive deficits associated with PAE.

It is unclear what drives this suppression of ribosome biogenesis and the invocation of nucleolar stress in the alcohol-exposed fetal cortex. One important clue emerges from our identification of 63 genes within the KEGG pathway cluster oxidative phosphorylation (Ox-Phos) as down-regulated in our transcriptome analysis of the E17.5 fetal brain. This is complemented by the parallel emergence of Pathways of Neurodegeneration, which also include numerous components of Ox-Phos. Alcohol is well known to reduce the activity of Ox-Phos in tissues including the brain [61] and in mouse NSCs [62], and we found this gene cluster to be similarly depressed in early neuroprogenitors [19]. Alcohol disrupts numerous mitochondrial properties, including mitogenesis, mitophagy, NADH regeneration, proton leakage, and the generation of superoxide radicals [63,64,65], all of which would reduce its capacity to generate ATP. Additionally, failures in rDNA transcription through small molecule or genetic means, independent of ribosome synthesis per se, can produce mitochondrial dysfunction via the activation of the DNA damage response, as shown for the neurodegenerative disorders Cockayne syndrome, spinocerebellar ataxia [17], and hypomyelinating leukodystrophy [66]. Regardless, fetal brain development is an energetically demanding process, consuming up to 60% of the total energy available to the body [67], and ribosome biogenesis itself places a high energetic demand [11], consuming 35–50% of nuclear transcription effort in proliferating eukaryotic cells, as well as meeting the high translational needs during axonogenesis and synaptic formation and remodeling [17]. We speculate the reductions in Ox-Phos activity could be an instigating factor that drives or accelerates nucleolar stress in these alcohol-exposed cells.

## 5. Conclusions

In summary, we show that alcohol exposures that cause deficits in cognition but not somatic growth induce nucleolar stress in primary NSCs through an impairment of ribosome biogenesis, and this activates P53/MDM2-mediated signaling to arrest NSC proliferation and induce their apoptosis. This nucleolar stress is also observed in the intact fetal cortex at pharmacologically relevant alcohol exposures and is accompanied by the suppression of multiple components of ribosome biogenesis. This nucleolar stress also occurs in cranial neural crest obtained from avian, rodent, and teleost embryos (Flentke et al., in revision), which indicates this likely represents a conserved response to alcohol exposure. These findings offer novel insights into the mechanisms by which alcohol causes growth arrest and apoptosis of neuronal progenitors through its induction of p53-mediated signaling. These findings suggest that PAE should be considered among the list of neurobehavioral and neurodegenerative syndromes that reflect the contributions of ribosomopathy. Further research is warranted to elucidate the precise molecular events underlying alcohol-induced nucleolar stress, as well as the specific downstream targets and regulatory mechanisms of this p53/MDM2-mediated apoptosis pathway. Identification of these will inform the potential development of neuroprotective interventions to mitigate PAE’s adverse impact on brain development.

## Figures and Tables

**Table 1:** KEGG pathways and their gene sets dysregulated by prenatal alcohol in the E17.5 fetal brain.

KEGG Pathway	KEGG ID	Enrichment Score	# Significant/ Total Genes ^1^	Padj	Gene Name
**Up-Regulated Genes**					
Cytokine–cytokine receptor interaction	mmu04060	2.26	15/35	0.0026	*Acvr1c*, *Bmp5*, *Bmp6*, *Bmp7*, *Bmpr2*, *Csf1*, *Csf1r*, *Cxcl12*, *Gdf11*, *Il17ra*, *Il6ra*, *Inha*, *Lepr*, *Tgfb3*, *Tgfbr2*
Complement and coagulation cascades	mmu04610	2.73	13/17	0.0026	*A2m*, *C3*, *Cd46*, *Cfb*, *Cfh*, *F13a1*, *Fga*, *Fgb*, *Fgg*, *Serpind1*, *Tfpi*, *Thbd*, *Vwf*
Hippo signaling pathway	mmu04390	2.34	28/53	0.0026	*Afp*, *Amot*, *Apc*, *Axin2*, *Bmp5*, *Fmp6*, *Bmp7*, *Bmpr2*, *Cdh1*, *Dlg1*, *Dvl3*, *Fzd1*, *Fzd3*, *Fzd5*, *Fzd7*, *Fzd8*, *Gsk3b*, *Lats2*, *Mob1b*, *Smad3*, *Tcf7l1*, *Tead1*, *Tgfb3*, *Tgfbr2*, *Trp73*, *Wnt2b*, *Wwtr1*, *Yap1*
Focal adhesion	mmu04510	2.35	37/48	0.0026	*Col1a1*, *Col1a2*, *Col2a1*, *Col4a1*, *Col4a2*, *Col4a5*, *Col4a6*, *Col6a1*, *Col6a2*, *Col6a3*, *Flnb*, *Flnc*, *Fn1*, *Igf1r*, *Itga1*, *Itga11*, *Itga2*, *Itga5*, *Itgb3*, *Kdr*, *Lama1*, *Lama2*, *Lamb1*, *Lamb2*, *Lamc1*, *Lamc3*, *Mylk*, *Pdgfrb*, *Pxn*, *Reln*, *Rock1*, *Thbs2*, *Thbs3*, *Tnr*, *Vav3*, *Vegfa*, *Vwf*
**Down-Regulated Genes**					
Oxidative phosphorylation	mmu00190	−2.61	63/69	0.0026	*Atp5a1*, *Atp5b*, *Atp5c1*, *Atp5f1*, *Atp5g1*, *Atp5g3*, *Atp5j2*, *Atp5k*, *Atp5o*, *Atp6v0d1*, *Atp6v0e2*, *Atp6v1b2*, *Atp6v1f*, *Atp6v1h*, *Cox10*, *Cox11*, *Cox17*, *Cox5a*, *Cox5b*, *Cox6a1*, *Cox6b1*, *Cox7a2*, *Cox7a2l*, *Cox7b*, *Cox8a*, *Cyc1*, *Ndufa10*, *Ndufa13*, *Ndufa2*, *Ndufa3*, *Ndufa6*, *Ndufa7*, *Ndufa8*, *Ndufa9*, *Ndufb10*, *Ndufb11*, *Ndufb2*, *Ndufb4*, *Ndufb5*, *Ndufb6*, *Ndufb7*, *Ndufb8*, *Ndufb9*, *Ndufc1*, *Ndufc2*, *Ndufs4*, *Ndufs6*, *Ndufs7*, *Ndufs8*, *Ndufv1*, *Ndufv2*, *Ndufv3*, *Ppa1*, *Ppa2*, *Sdhb*, *Sdhc*, *Sdhd*, *Uqcr10*, *Uqcr11*, *Uqcrc1*, *Uqcrc2*, *Uqcrfs1*, *Uqcrq*
Ribosome	mmu03010	−2.46	74/94	0.0026	*Mrpl10*, *Mrpl11*, *Mrpl12*, *Mrpl13*, *Mrpl14*, *Mrpl15*, *Mrpl18*, *Mrpl19*, *Mrpl2*, *Mrpl20*, *Mrpl22*, *Mrpl27*, *Mrpl28*, *Mrpl3*, *Mrpl33*, *Mrpl34*, *Mrpl9*, *Mrps10*, *Mrps11*, *Mrps12*, *Mrps17*, *Mrps18a*, *Mrps21*, *Mrps6*, *Mrps7*, *Rpl3*, *Rpl6*, *Rpl8*, *Rpl11*, *Rpl12*, *Rpl13*, *Rpl13a*, *Rpl14*, *Rpl15*, *Rpl18*, *Rpl22l1*, *Rpl27*, *Rpl27a*, *Rpl28*, *Rpl29*, *Rpl30*, *Rpl31*, *Rpl35*, *Rpl36*, *Rpl36a*, *Rpl37*, *Rpl38*, *Rpl39*, *Rpl41*, *Rplp0*, *Rplp1*, *Rps2*, *Rps3*, *Rps5*, *Rps6*, *Rps7*, *Rps8*, *Rps9*, *Rps10*, *Rps13*, *Rps15*, *Rps16*, *Rps17*, *Rps18*, *Rps20*, *Rps21*, *Rps23*, *Rps24*, *Rps27a*, *Rps28*, *Rps29*, *Rpsa*
Pathways of Neurodegeneration	mmu05022	−1.74	93/143	0.0026	*Actr10*, *Actr1a*, *Atf4*, *Atp5a1*, *Atp5b*, *Atp5c1*, *Atp5f1*, *Atp5g1*, *Atp5h*, *Atp5o*, *Bad*, *Cox4i1*, *Cox5a*, *Cox5b*, *Cox6a1*, *Cox6b1*, *Cox7a2*, *Cox7a2l*, *Cox7b*, *Cox8a*, *Cyc1*, *Cycs*, *Daxx*, *Dctn3*, *Gabarap*, *Gpx1*, *Hsd17b10*, *Ift57*, *Ndufa10*, *Ndufa3*, *Ndufa2*, *Ndufa3*, *Ndufa6*, *Ndufa7*, *Ndufa8*, *Ndufa9*, *Ndufc1*, *Ndufc2*, *Ndufs2*, *Ndufs4*, *Ndufs6*, *Ndufs7*, *Ndufs8*, *Ndufv1*, *Ndufv2*, *Ndufv3*, *Park7*, *Ppif*, *Psma6*, *Psma7*, *Psmb1*, *Psmb4*, *Psmb5*, *Psmb6*, *Psmb7*, *Psmc2*, *Psmc3*, *Psmc4*, *Psmc5*, *Psmd1*, *Psmd6*, *Psmd8*, *Psmd9*, *Rab8a*, *Rps27a*, *Sdhb*, *Sdhc*, *Sdhd*, *Septin5*, *Sigmar1*, *Sqstm1*, *Tomm40*, *Tom40l*, *Trap1*, *Tuba1c*, *Tubb4b*, *Ubb*, *Uqcr10*, *Uqcr11*, *Uqcrc1*, *Uqcrc2*, *Uqcrfs1*, *Uqcrq*, *Vdac3*, *Xbp1*

^1^ The number of genes represents the number significantly altered by alcohol with respect to the total number of genes in that KEGG pathway. The complete lists of KEGG pathways having altered representation in response to alcohol are presented in Tables S2 and S3.

**Table 2:** Gene expression of oxidative phosphorylation components correlates with fetal brain weight at E17.5 ^1^.

Gene	R	*p*-Value
*Atp5c1*	0.58	0.019
*Atp5K1*	0.56	0.023
*Atp6v0e2*	0.55	0.027
*Ndufa13*	0.57	0.020
*Ndufb2*	0.53	0.037
*Ndufb11*	0.53	0.037
*Ndufs4*	0.51	0.045
*Ndufs8*	0.66	0.005
*Ndufv3*	0.61	0.012
*Ppa2*	0.53	0.033
*Uqcrq*	0.54	0.032

^1^ N = 8 litters per group; fetal brain weights are the average of four fetuses per litter and correspond to the same four fetuses that were pooled for the RNA-Seq that generated these expression-level data. The data were analyzed by Pearson correlation.

## Data Availability

Data are either provided within the Supplementary Materials or can be solicited from the corresponding author by reasonable request.

## Supplementary Materials

The following supporting information can be downloaded at: https://www.mdpi.com/article/10.3390/cells13050440/s1. Figure S1: Conditional quantile normalization (cqn) corrects for gene-length bias in RNA-seq data from the alcohol-exposed and control fetal brains at E17.5. Figure S2: Nucleolar immunostaining of primary NSCs presented as separated color channels. Table S1: List of 2848 genes differentially expressed in the E17.5 alcohol-exposed fetal brain. Table S2: KEGG pathways and their differentially expressed gene components have increased expression in the alcohol-exposed E17.5 fetal brain compared with controls. Table S3: KEGG pathways and their differentially expressed gene components having reduced expression in the alcohol-exposed E17.5 fetal brain compared with controls. Table S4: KEGG pathways related to ribosome biogenesis that are dysregulated by PAE. Table S5: Alcohol-responsive genes in the E17.5 fetal brain that respond to p53.
