# Supplementary material for: Alcohol Exposure Induces Nucleolar Stress and Apoptosis in Mouse Neural Stem Cells and Late-Term Fetal Brain
Source: Cells. 2024 Mar 2;13(5):440. doi: 10.3390/cells13050440 (PMC10931382; doi:10.3390/cells13050440)
Supplement: Supplementary file 1 [file cells-13-00440-s001.zip › cells-2736275-supplementary.pdf]

Supplemental Information
Huang et al.
Nucleolar Stress in Alcohol-Exposed Mouse Neural Stem Cells and Late-Term Fetal Brain

Figure S1.
Figure S2.
Tables S1 – S5.

Figure S1. Conditional quantile normalization (cqn) corrects for gene-length bias in RNA-Seq data from mouse fetal brain. (a) RNA-Seq data from the E17.5 fetal brain analyzed using DE-Seq shows coupling between gene length and fold-change. **(b)** After applying cqn, fold-change is no longer skewed by gene length. The line is a linear regression. Data represent n=8 letters per group and four fetal brains pooled per litter.

Figure S2. Alcohol exposure induces nucleolar dissolution in primary neural stem cells (NSCs). Images are identical to Figure 3a but are expanded to separately visualize nuclei (DAPI, blue), the immunostain for nucleolar proteins UBF (a, green), fibrillarin (b, green), and nucleolin (c, green), and the merged channels. The merged channel images are presented in Figure 3a.

Supplemental Figure 1

1A

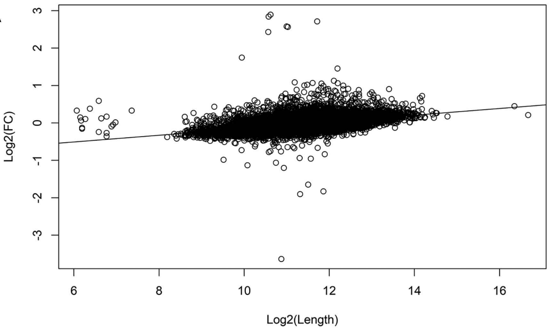

1B

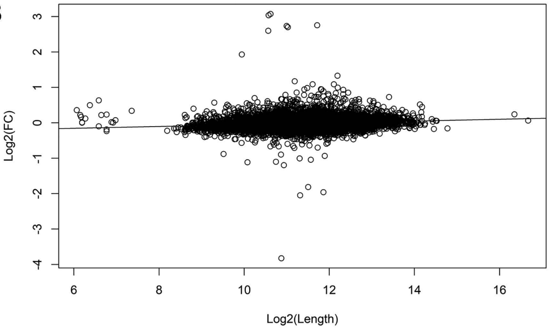

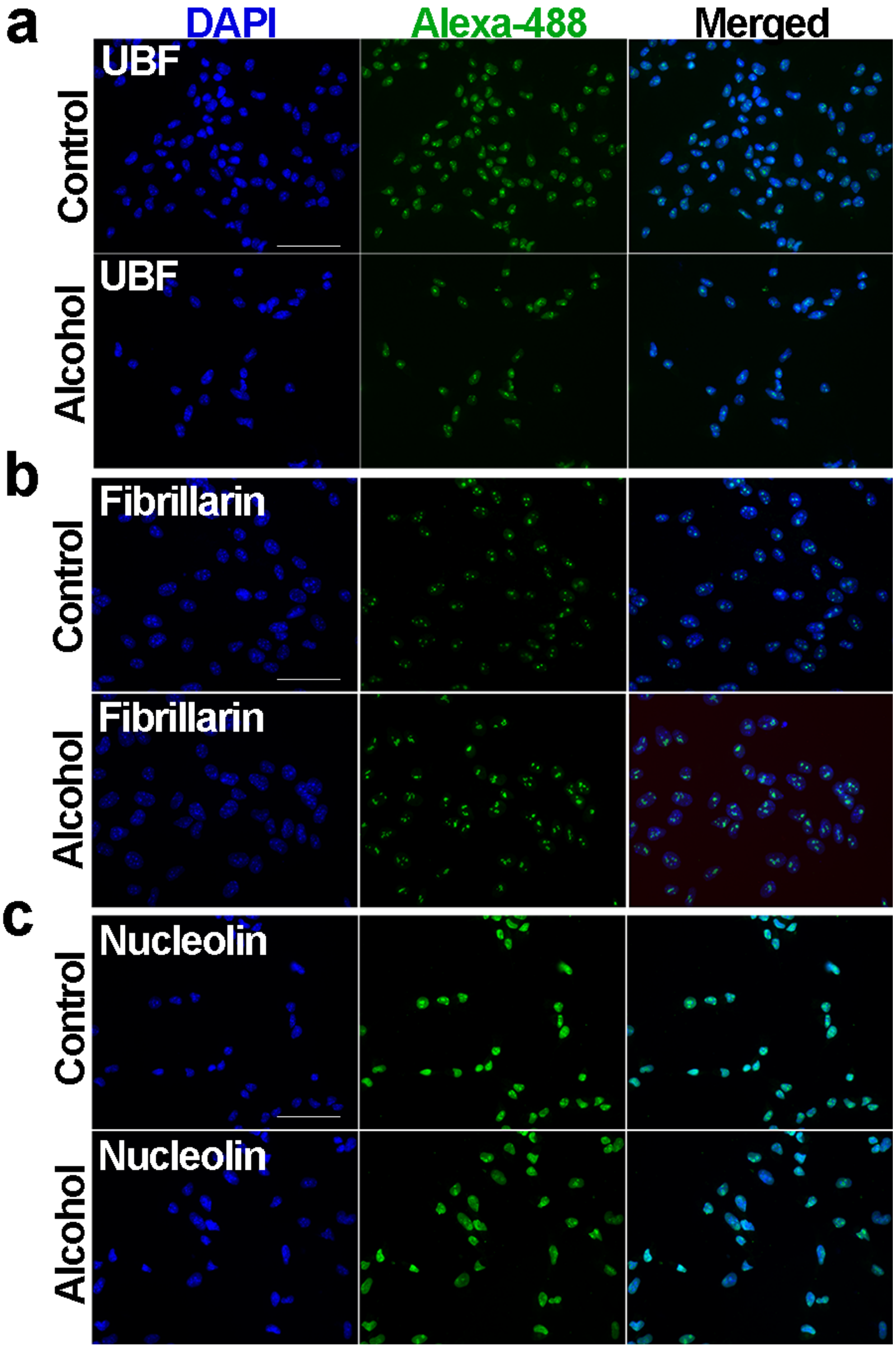

Figure S2. Alcohol exposure induces nucleolar dissolution in primary neural stem cells (NSCs). Images are identical to Figure 3a but are expanded to separately visualize nuclei (DAPI, blue), the immunostain for nucleolar proteins UBF (a, green), fibrillarin (b, green), and nucleolin (c, green), and the merged channels. The merged channel images are presented in Figure 3a.

Table S1 - List of 2848 genes differentially expressed in Alcohol-Exposed Fetal Brain.

Gene Name	Fold Change (Alc/Control)	*P* value	*P adj*
Sh3pxd2a	1.25	6.87E-14	5.43E-10
Ppp1r3b	1.58	8.28E-14	5.43E-10
Itih2	2.13	5.07E-13	2.21E-09
Wdr82	0.88	2.30E-12	7.55E-09
Grin2b	1.25	5.32E-12	1.40E-08
Fn1	1.47	9.05E-12	1.98E-08
Alb	6.65	1.38E-11	2.00E-08
Psmd8	0.89	1.24E-11	2.00E-08
Hsd17b10	0.88	1.20E-11	2.00E-08
Itih5	1.55	1.78E-11	2.18E-08
Hmgcs2	1.35	1.83E-11	2.18E-08
Fbn1	1.36	2.30E-11	2.51E-08
Med11	0.83	4.02E-11	4.05E-08
Msi2	1.16	4.65E-11	4.11E-08
Ndufa13	0.84	4.70E-11	4.11E-08
Ahnak	1.36	5.69E-11	4.66E-08
Nagk	0.85	1.28E-10	9.85E-08
Afp	6.51	1.84E-10	1.34E-07
Lrp1	1.08	2.09E-10	1.44E-07
Apoa1	3.81	2.28E-10	1.49E-07
Col4a1	1.29	3.07E-10	1.91E-07
Col4a2	1.26	3.29E-10	1.96E-07
Psmb1	0.91	3.78E-10	2.15E-07
Hsd17b12	0.86	4.22E-10	2.30E-07
Tubb3	0.95	6.21E-10	3.25E-07
Cyp1b1	1.75	6.83E-10	3.44E-07
Tmem192	0.80	7.39E-10	3.59E-07
Ahsg	6.07	7.80E-10	3.65E-07
Vwf	1.30	8.80E-10	3.98E-07
Hspg2	1.23	9.45E-10	4.13E-07
Atf4	0.80	1.46E-09	6.01E-07
Thsd4	1.42	1.47E-09	6.01E-07
Taok1	1.12	1.60E-09	6.36E-07
Cct5	0.88	1.72E-09	6.63E-07
Igsf3	1.10	1.98E-09	7.43E-07
Mthfd2	0.66	2.26E-09	8.17E-07
Trf	2.26	2.31E-09	8.17E-07
Rpl15	0.90	2.59E-09	8.70E-07
Bsn	1.03	2.54E-09	8.70E-07
Cox6a1	0.88	2.69E-09	8.83E-07
Lpp	1.20	2.77E-09	8.86E-07
Med30	0.87	3.00E-09	9.15E-07
Fga	6.75	3.00E-09	9.15E-07
Farsb	0.87	3.37E-09	9.69E-07
Zfp704	1.10	3.41E-09	9.69E-07
Park7	0.91	3.47E-09	9.69E-07

Hif3a	1.34	3.42E-09	9.69E-07
Nfasc	1.14	3.62E-09	9.87E-07
Cops7a	0.85	4.08E-09	1.09E-06
Prmt5	0.89	4.35E-09	1.14E-06
Anpep	1.65	5.46E-09	1.40E-06
Coa3	0.87	6.01E-09	1.48E-06
Dctn3	0.89	5.95E-09	1.48E-06
Usmg5	0.83	6.43E-09	1.56E-06
Ndufs2	0.89	7.61E-09	1.81E-06
Tbcb	0.85	8.02E-09	1.88E-06
Aimp2	0.82	8.33E-09	1.91E-06
Tkt	0.88	9.06E-09	2.05E-06
Cbl	1.16	9.30E-09	2.07E-06
Hlf	1.27	9.48E-09	2.07E-06
Reln	1.32	9.67E-09	2.08E-06
Mrpl11	0.83	1.06E-08	2.25E-06
Tnrc6b	1.12	1.22E-08	2.53E-06
Actg1	0.92	1.28E-08	2.63E-06
Thbs2	1.42	1.33E-08	2.67E-06
Lpar1	1.32	1.34E-08	2.67E-06
Kcnq3	1.25	1.44E-08	2.77E-06
Cops4	0.90	1.44E-08	2.77E-06
Atxn10	0.90	1.55E-08	2.94E-06
Flnb	1.12	1.84E-08	3.44E-06
Col6a2	1.68	1.88E-08	3.44E-06
Sumo3	0.89	1.89E-08	3.44E-06
Cops5	0.89	1.98E-08	3.50E-06
Igf2	1.53	1.97E-08	3.50E-06
Dapk3	0.87	2.37E-08	4.14E-06
Scara3	1.23	2.43E-08	4.18E-06
Lpgat1	1.03	2.64E-08	4.46E-06
Rorb	1.21	2.66E-08	4.46E-06
Spag7	0.92	2.73E-08	4.52E-06
Znhit1	0.86	2.99E-08	4.90E-06
Ndufa6	0.91	3.13E-08	5.06E-06
Fgg	8.22	3.20E-08	5.11E-06
Gpr4	1.35	3.42E-08	5.40E-06
Nudt19	0.85	3.53E-08	5.51E-06
Psph	0.79	4.52E-08	6.96E-06
Psmb6	0.91	5.08E-08	7.55E-06
Col5a1	1.53	5.06E-08	7.55E-06
Sars	0.89	5.12E-08	7.55E-06
Grcc10	0.90	5.13E-08	7.55E-06
BC031181	0.94	5.22E-08	7.60E-06
Wnk1	1.11	5.49E-08	7.91E-06
Eif3g	0.94	5.56E-08	7.92E-06
Col6a3	1.66	5.97E-08	8.41E-06
Mrps6	0.88	6.11E-08	8.51E-06
Mrpl15	0.80	6.64E-08	9.07E-06

Psma7	0.92	6.62E-08	9.07E-06
Cct3	0.87	7.03E-08	9.49E-06
Amotl1	1.12	7.10E-08	9.49E-06
Ap1m1	0.92	7.45E-08	9.86E-06
Fat3	1.13	7.66E-08	1.00E-05
Ppia	0.90	7.76E-08	1.01E-05
Ndufa10	0.90	7.93E-08	1.01E-05
Yars	0.83	7.97E-08	1.01E-05
Zbtb37	1.22	8.18E-08	1.03E-05
Aebp1	1.71	8.71E-08	1.09E-05
Kdr	1.17	8.93E-08	1.10E-05
Ndufa9	0.89	9.07E-08	1.11E-05
Ndufs8	0.80	9.23E-08	1.12E-05
Morn4	0.93	9.31E-08	1.12E-05
Tmem120a	0.87	1.00E-07	1.19E-05
Phb2	0.92	1.00E-07	1.19E-05
Tmem170b	1.07	1.05E-07	1.23E-05
Srm	0.89	1.07E-07	1.24E-05
Phc3	1.10	1.09E-07	1.25E-05
Foxd1	1.58	1.17E-07	1.32E-05
Prmt7	0.81	1.16E-07	1.32E-05
Dcp2	1.07	1.21E-07	1.36E-05
Lox	1.93	1.28E-07	1.42E-05
Hint1	0.89	1.29E-07	1.42E-05
Ddx25	0.89	1.32E-07	1.44E-05
Tnrc6c	1.07	1.34E-07	1.45E-05
Hipk1	1.07	1.43E-07	1.53E-05
Rpl41	0.87	1.56E-07	1.65E-05
Sipa1l1	1.13	1.56E-07	1.65E-05
Gm6787	0.86	1.69E-07	1.77E-05
Gjb2	1.81	1.71E-07	1.78E-05
Klhl7	1.18	1.73E-07	1.79E-05
Adam12	1.33	1.75E-07	1.79E-05
Kctd12	1.13	1.91E-07	1.93E-05
Ndufaf5	0.79	1.92E-07	1.93E-05
Gsk3b	1.06	2.01E-07	1.98E-05
Mrc1	1.58	2.00E-07	1.98E-05
Pafah1b3	0.90	2.03E-07	1.98E-05
E130201H02Rik	0.91	2.03E-07	1.98E-05
Hoxa5	0.07	2.07E-07	2.00E-05
Lamc1	1.18	2.08E-07	2.00E-05
Ccdc93	1.05	2.13E-07	2.02E-05
Lamc3	1.54	2.14E-07	2.02E-05
Col4a5	1.34	2.12E-07	2.02E-05
Olfml2a	1.59	2.20E-07	2.06E-05
Igf2r	1.16	2.24E-07	2.08E-05
Otub1	0.91	2.37E-07	2.17E-05
Foxc2	1.92	2.36E-07	2.17E-05
Abca9	1.56	2.46E-07	2.24E-05

Uros	0.84	2.50E-07	2.24E-05
Mrpl34	0.90	2.49E-07	2.24E-05
Mdga1	1.10	2.53E-07	2.26E-05
Mrc2	1.32	2.57E-07	2.27E-05
Rpl3	0.92	2.58E-07	2.27E-05
Chchd6	0.83	2.62E-07	2.28E-05
Dst	1.04	2.68E-07	2.32E-05
Sept5	0.93	2.75E-07	2.37E-05
Epas1	1.18	2.88E-07	2.46E-05
Fam168a	1.01	3.14E-07	2.67E-05
Rc3h1	1.06	3.21E-07	2.71E-05
Colec12	1.66	3.23E-07	2.71E-05
Drap1	0.91	3.36E-07	2.81E-05
Srgap3	1.11	3.49E-07	2.89E-05
Tsc22d2	1.07	3.65E-07	3.01E-05
Cops6	0.87	3.85E-07	3.15E-05
Prrc2b	1.03	4.12E-07	3.35E-05
Nfat5	1.29	4.14E-07	3.35E-05
Gxylt1	1.24	4.22E-07	3.38E-05
Fgb	8.43	4.23E-07	3.38E-05
Ino80d	1.10	4.52E-07	3.59E-05
Cacna1e	1.22	4.58E-07	3.61E-05
Col5a2	1.31	4.76E-07	3.73E-05
Serpinb1b	0.47	4.81E-07	3.75E-05
Atp5b	0.89	4.85E-07	3.76E-05
Col1a1	1.99	4.89E-07	3.77E-05
Ankrd44	1.11	5.02E-07	3.84E-05
Bmp6	1.44	5.08E-07	3.84E-05
Vapa	0.94	5.10E-07	3.84E-05
Psmc4	0.90	5.05E-07	3.84E-05
Ildr2	1.18	5.30E-07	3.97E-05
Tomm40l	0.82	5.33E-07	3.97E-05
Ankrd52	1.09	5.54E-07	4.10E-05
Pcnx	1.03	5.62E-07	4.12E-05
Agrn	1.07	5.63E-07	4.12E-05
Phb	0.87	5.71E-07	4.16E-05
Gja1	1.34	5.78E-07	4.18E-05
Ppme1	0.91	5.93E-07	4.27E-05
Dnajc8	0.94	6.13E-07	4.39E-05
Eef1e1	0.89	6.60E-07	4.67E-05
Lcor	1.22	6.65E-07	4.67E-05
Ttc39b	1.11	6.67E-07	4.67E-05
Mrps12	0.88	6.60E-07	4.67E-05
Nt5c3	0.88	6.82E-07	4.75E-05
Smyd5	0.84	6.85E-07	4.75E-05
Asns	0.78	7.04E-07	4.85E-05
Mrpl54	0.80	7.46E-07	5.09E-05
Ahsa1	0.93	7.50E-07	5.09E-05
Sdhb	0.88	7.43E-07	5.09E-05

Lamb2	1.20	7.83E-07	5.29E-05
Dennd5b	1.08	8.12E-07	5.45E-05
Celsr2	1.09	8.59E-07	5.74E-05
Slc13a4	1.74	9.19E-07	6.01E-05
Zfp568	1.10	9.17E-07	6.01E-05
Ndufb7	0.86	9.06E-07	6.01E-05
Glg1	1.14	9.22E-07	6.01E-05
Nsdhl	0.83	9.20E-07	6.01E-05
Dynll1	0.88	9.34E-07	6.04E-05
Fam136a	0.90	9.37E-07	6.04E-05
Coq7	0.84	9.41E-07	6.04E-05
Nav2	1.13	9.45E-07	6.04E-05
Gars	0.88	9.97E-07	6.34E-05
Flrt1	1.12	1.01E-06	6.38E-05
Igf1r	1.16	1.01E-06	6.38E-05
Dync1li2	1.05	1.02E-06	6.42E-05
Pzp	2.52	1.03E-06	6.44E-05
Ubn2	1.15	1.05E-06	6.54E-05
Eif3d	0.89	1.07E-06	6.59E-05
Rad54l2	1.07	1.07E-06	6.59E-05
1300002E11Rik	1.24	1.12E-06	6.88E-05
Elp3	0.91	1.16E-06	7.08E-05
Slc4a1	1.79	1.20E-06	7.20E-05
Mdh2	0.91	1.21E-06	7.20E-05
Cars	0.80	1.20E-06	7.20E-05
Cdh1	2.13	1.21E-06	7.20E-05
Csnk1g1	1.08	1.20E-06	7.20E-05
Nufip2	1.08	1.22E-06	7.26E-05
Gucy1a2	1.11	1.23E-06	7.27E-05
Fdft1	0.82	1.29E-06	7.58E-05
Lsm4	0.89	1.30E-06	7.62E-05
Serinc5	1.15	1.32E-06	7.69E-05
Vti1b	0.89	1.36E-06	7.88E-05
Chd2	1.15	1.39E-06	8.03E-05
Trio	1.04	1.42E-06	8.15E-05
Rbx1	0.88	1.42E-06	8.15E-05
Man2a1	1.20	1.44E-06	8.19E-05
Mvd	0.80	1.47E-06	8.32E-05
Cct6a	0.94	1.48E-06	8.33E-05
Ndufb9	0.88	1.52E-06	8.52E-05
Ccdc50	1.04	1.52E-06	8.52E-05
Bckdhb	0.78	1.54E-06	8.56E-05
Ssu72	0.86	1.62E-06	9.01E-05
Cdh5	1.40	1.68E-06	9.30E-05
Zfp36l1	1.19	1.71E-06	9.39E-05
Itpa	0.87	1.71E-06	9.39E-05
Bud31	0.93	1.73E-06	9.46E-05
Sqle	0.86	1.75E-06	9.50E-05
Fat4	1.15	1.77E-06	9.59E-05

Kars	0.89	1.81E-06	9.74E-05
Ppa1	0.91	1.81E-06	9.75E-05
Zfp871	1.12	1.84E-06	9.84E-05
Dars	0.89	1.91E-06	0.00010
Sdhc	0.91	1.92E-06	0.00010162
Insr	1.12	1.93E-06	0.000102041
Vasn	1.32	1.94E-06	0.000102339
Mmp2	1.27	1.96E-06	0.000102739
Tbx15	1.93	1.99E-06	0.000103766
Ywhaq	0.89	2.02E-06	0.000104946
Tulp4	1.06	2.06E-06	0.000106529
Islr	1.53	2.10E-06	0.000108395
Purb	1.08	2.24E-06	0.000115274
Adam22	1.08	2.34E-06	0.000119825
Chchd3	0.89	2.35E-06	0.000119825
Cct7	0.87	2.38E-06	0.000120889
Shank1	1.11	2.41E-06	0.00012215
Mxra8	1.31	2.44E-06	0.000123166
Hook3	1.17	2.49E-06	0.000124795
Atrnl1	1.07	2.50E-06	0.000124987
Chchd1	0.92	2.56E-06	0.000127584
Kctd13	0.94	2.59E-06	0.000128671
Col12a1	1.19	2.65E-06	0.000131259
Slc25a5	0.93	2.70E-06	0.000132775
Mapkbp1	1.08	2.71E-06	0.000132803
Myh9	1.14	2.73E-06	0.000133554
Hmcn1	1.23	2.75E-06	0.000134025
Aip	0.83	2.84E-06	0.000137972
Letm1	0.89	2.87E-06	0.000138661
Gsto1	0.92	2.90E-06	0.000139141
Kif1b	1.03	2.90E-06	0.000139141
Nhp2	0.86	2.98E-06	0.00014007
Tmem242	0.89	2.98E-06	0.00014007
Ptprf	1.02	2.96E-06	0.00014007
Bub3	0.91	2.96E-06	0.00014007
Asna1	0.92	2.98E-06	0.00014007
Timm8b	0.91	2.95E-06	0.00014007
Syde2	1.14	3.00E-06	0.00014
Col18a1	1.36	3.08E-06	0.00014
Srp14	0.90	3.06E-06	0.00014
Sec13	0.90	3.08E-06	0.00014
Rpl28	0.93	3.11E-06	0.00014
Ndufc2	0.90	3.12E-06	0.00014
Ppp1ca	0.93	3.17E-06	0.00014
Grpel1	0.82	3.15E-06	0.00014
Fyco1	1.16	3.14E-06	0.00014
Timm17b	0.87	3.16E-06	0.00014
Mrpl43	0.91	3.20E-06	0.00014
Timm10	0.87	3.21E-06	0.00014

Zkscan2	1.16	3.23E-06	0.00014
Ndufb11	0.89	3.27E-06	0.00015
Nid2	1.36	3.33E-06	0.00015
St3gal3	0.84	3.38E-06	0.000150104
Lepr	1.59	3.44E-06	0.00015
Ttbk2	1.04	3.47E-06	0.00015
Mrps7	0.86	3.64E-06	0.000159927
Atp5c1	0.88	3.66E-06	0.000160268
Slco2a1	1.26	3.75E-06	0.00016
Col15a1	1.27	3.78E-06	0.00016
Tmem11	0.88	3.93E-06	0.000169951
Cacna2d1	1.12	3.92E-06	0.000169951
Cacna1c	1.15	4.00E-06	0.00017
Neo1	1.08	4.01E-06	0.00017
Ostc	0.93	4.07E-06	0.00017
Ubb	0.93	4.14E-06	0.00018
Runx1t1	1.12	4.17E-06	0.00018
Prrx1	1.16	4.25E-06	0.000180056
Cfh	1.53	4.29E-06	0.00018
Mrpl12	0.88	4.28E-06	0.00018
Zbtb39	1.07	4.46E-06	0.00019
Uqcrc1	0.88	4.50E-06	0.00019
Pcdhb16	1.16	4.55E-06	0.00019
Zbtb34	1.10	4.54E-06	0.00019
Rpl18	0.86	4.53E-06	0.00019
Txn2	0.90	4.74E-06	0.00020
Mif	0.91	4.79E-06	0.00020
Ranbp3l	1.70	4.89E-06	0.00020
Ten1	0.86	4.93E-06	0.00020
Alkbh7	0.80	5.00E-06	0.00020
Mea1	0.87	5.02E-06	0.00020
Fgd6	1.12	5.15E-06	0.00021
Rab33a	0.92	5.14E-06	0.00021
Psmd6	0.92	5.32E-06	0.00021
Nphp3	1.15	5.34E-06	0.00021
Actl6b	0.94	5.40E-06	0.00022
Slc25a33	0.77	5.52E-06	0.000220349
Sh3pxd2b	1.04	5.57E-06	0.00022
Nav1	1.06	5.72E-06	0.00023
Atp5a1	0.91	5.89E-06	0.00023
Xpr1	1.10	6.00E-06	0.00024
Serpinf1	1.47	6.05E-06	0.00024
Psmc5	0.91	6.19E-06	0.00024
Thap7	0.87	6.17E-06	0.00024
Tcp1	0.89	6.18E-06	0.00024
Psmc3	0.91	6.15E-06	0.00024
Eif3k	0.93	6.19E-06	0.00024
Tead1	1.17	6.11E-06	0.00024
Col4a6	1.50	6.15E-06	0.00024

Bcl2	1.03	6.34E-06	0.00024
Ddr2	1.38	6.37E-06	0.00024
Ncor2	1.06	6.39E-06	0.00024
Eef1b2	0.83	6.42E-06	0.00024
Aff1	1.14	6.45E-06	0.00025
Acat2	0.89	6.47E-06	0.00025
Ptprb	1.11	6.59E-06	0.00025
D430041D05Rik	1.10	6.59E-06	0.00025
Mrpl33	0.89	6.63E-06	0.00025
Mafa	0.46	6.79E-06	0.00025
Ndufv2	0.90	6.78E-06	0.00025
Adsl	0.86	6.98E-06	0.000259816
Wdfy3	1.03	7.03E-06	0.00026
Slc25a1	0.88	7.10E-06	0.00026
Arpc3	0.97	7.11E-06	0.00026
Col1a2	1.91	7.15E-06	0.00026
Banf1	0.91	7.17E-06	0.00026
Fbn2	1.23	7.56E-06	0.00028
Nol7	0.91	7.73E-06	0.00028
Tomm40	0.89	7.72E-06	0.00028
Gpc3	1.39	7.70E-06	0.00028
Aen	0.83	7.89E-06	0.00029
Nsd1	1.04	8.04E-06	0.000290356
Spag9	1.05	8.26E-06	0.00030
Ndufs4	0.87	8.34E-06	0.00030
Sdk1	1.09	8.35E-06	0.00030
Tm2d2	0.92	8.38E-06	0.00030
Wdr18	0.86	8.73E-06	0.00031
Eif3h	0.92	8.76E-06	0.00031
Rpl11	0.91	8.88E-06	0.00031
Slc2a12	1.26	8.96E-06	0.00032
Ltbp4	1.14	8.95E-06	0.00032
Slc38a6	1.20	9.07E-06	0.00032
Foxc1	1.37	9.05E-06	0.00032
Acer2	1.39	9.09E-06	0.00032
Glyr1	0.93	9.21E-06	0.00032
Zc3hav1l	1.08	9.24E-06	0.00032
Gabarap	0.91	9.29E-06	0.00032
Atp5j	0.96	9.41E-06	0.00033
Ttll12	0.87	9.49E-06	0.00033
Cox6b1	0.92	9.50E-06	0.00033
Phldb2	1.20	9.61E-06	0.000329681
Ndufaf3	0.85	9.67E-06	0.00033
Tgm2	1.37	9.72E-06	0.00033
Uba52	0.94	9.88E-06	0.00034
Egflam	1.26	1.01E-05	0.00034
Map3k2	1.09	1.01E-05	0.00034
Psma6	0.92	1.02E-05	0.00034
Clpp	0.87	1.04E-05	0.00035

Tubb5	0.95	1.04E-05	0.00035
Dhps	0.85	1.05E-05	0.00035
Gnaq	1.07	1.06E-05	0.00035
Uqcrq	0.84	1.06E-05	0.00035
Mtx2	0.92	1.07E-05	0.00035
Itgav	1.04	1.07E-05	0.00036
Svil	1.23	1.09E-05	0.00036
Rpsa	0.86	1.09E-05	0.00036
Emilin1	1.44	1.11E-05	0.00036
S100a10	0.90	1.11E-05	0.00036
Trps1	1.05	1.14E-05	0.00037
Hivep3	1.08	1.14E-05	0.00037
Acap2	1.14	1.15E-05	0.00037
Tprgl	0.88	1.15E-05	0.00037
Ppp5c	0.89	1.15E-05	0.00037
Plekhn1	1.32	1.16E-05	0.00037
Lyrm2	0.92	1.16E-05	0.00037
Eif2b5	0.88	1.18E-05	0.00038
Dnaja1	0.88	1.19E-05	0.00038
Tanc2	1.08	1.21E-05	0.00039
Thsd7a	1.14	1.21E-05	0.00039
Puf60	0.88	1.23E-05	0.00039
Ndst1	1.05	1.23E-05	0.00039
Fmod	1.98	1.25E-05	0.00039
Ndufb8	0.86	1.24E-05	0.00039
Cfl1	0.97	1.28E-05	0.00040
Slc6a13	1.59	1.29E-05	0.00041
Tnks2	1.03	1.30E-05	0.00041
Psmb7	0.91	1.30E-05	0.00041
Srgap1	1.12	1.31E-05	0.00041
Snx33	1.18	1.32E-05	0.00041
Nme1	0.89	1.33E-05	0.00042
Eif2s2	0.91	1.34E-05	0.00042
Rer1	0.91	1.35E-05	0.00042
Fzd3	1.11	1.36E-05	0.00042
2810428I15Rik	0.87	1.38E-05	0.00042
Ssr4	0.88	1.37E-05	0.00042
Col6a1	1.59	1.39E-05	0.00043
Thbd	1.86	1.40E-05	0.00043
Herc1	1.06	1.40E-05	0.00043
Opa3	0.86	1.41E-05	0.00043
Chic1	1.09	1.41E-05	0.00043
Rps19bp1	0.90	1.42E-05	0.00043
Senp1	1.05	1.46E-05	0.00044
Drg1	0.94	1.46E-05	0.00044
Sacs	1.18	1.47E-05	0.00044
Dpy30	0.92	1.48E-05	0.00045
1810013L24Rik	1.09	1.50E-05	0.00045
Uqcrc2	0.90	1.49E-05	0.00045

2410004B18Rik	0.90	1.51E-05	0.00045
Rps15	0.91	1.53E-05	0.00046
Mrps28	0.81	1.59E-05	0.00047
Slc30a7	1.06	1.59E-05	0.00047
Krtcap2	0.92	1.62E-05	0.000479768
Crcp	0.89	1.63E-05	0.000479768
Ppil1	0.93	1.63E-05	0.000480039
Abca1	1.14	1.63E-05	0.000480039
Rpl29	0.95	1.64E-05	0.00048
Pfdn1	0.89	1.64E-05	0.00048
Cct4	0.89	1.65E-05	0.00048
Rgs2	0.85	1.68E-05	0.00049
Akt1s1	0.87	1.68E-05	0.00049
Luc7l2	1.12	1.69E-05	0.00049
Scube3	1.16	1.72E-05	0.00050
Hars	0.89	1.73E-05	0.00050
Helz	1.11	1.74E-05	0.00050
Dicer1	1.08	1.74E-05	0.00050
Cp	1.34	1.77E-05	0.00051
Ndufv1	0.90	1.81E-05	0.00052
Ssc5d	1.43	1.81E-05	0.00052
2310039H08Rik	0.82	1.81E-05	0.00052
Insig1	0.83	1.82E-05	0.00052
Capza2	0.91	1.86E-05	0.00053
Rplp1	0.91	1.89E-05	0.00054
Sorbs1	1.09	1.90E-05	0.00054
Ywhae	0.93	1.99E-05	0.00056
Efr3a	1.09	2.00E-05	0.00056
Zmat3	1.04	2.02E-05	0.00057
Ndufaf2	0.91	2.04E-05	0.00057
Ubr2	1.07	2.04E-05	0.00057
Ubald1	0.93	2.04E-05	0.00057
Slc44a5	1.13	2.08E-05	0.00058
Tpgs1	0.86	2.11E-05	0.00058
Ndufb10	0.92	2.11E-05	0.00058
Faxc	1.03	2.11E-05	0.00058
Cox4i1	0.89	2.15E-05	0.00059
Dcx	1.11	2.14E-05	0.00059
Zfp329	1.08	2.16E-05	0.00059
Rpl37	0.87	2.16E-05	0.00059
Fan1	1.16	2.19E-05	0.000599611
Angptl2	1.40	2.21E-05	0.00060
Psme1	0.93	2.22E-05	0.00060
Cinp	0.87	2.23E-05	0.00060
Nid1	1.23	2.23E-05	0.00060
Dock3	1.05	2.23E-05	0.00060
Glrx5	0.91	2.26E-05	0.000610266
Foxn3	1.21	2.31E-05	0.00062
Crip2	0.94	2.33E-05	0.00063

Etv4	0.73	2.36E-05	0.00063
Zbtb10	1.10	2.36E-05	0.00063
Kalrn	1.05	2.38E-05	0.00063
Eif3b	0.91	2.37E-05	0.00063
Arhgef12	1.03	2.39E-05	0.00064
2900026A02Rik	1.13	2.40E-05	0.00064
Bysl	0.83	2.43E-05	0.00064
Tomm6	0.91	2.45E-05	0.00065
Ltbp3	1.13	2.46E-05	0.00065
Slc25a38	0.85	2.47E-05	0.00065
Idh1	0.95	2.50E-05	0.00065
Sec11c	0.88	2.49E-05	0.00065
Aars	0.84	2.49E-05	0.00065
Micu1	0.89	2.51E-05	0.00066
Tuba1c	0.92	2.53E-05	0.00066
Slc13a3	1.36	2.54E-05	0.00066
Asap3	1.22	2.54E-05	0.00066
Aurkaip1	0.90	2.62E-05	0.00068
Cmtm4	1.02	2.62E-05	0.00068
Gpc6	1.08	2.67E-05	0.00069
Fgfr2	1.11	2.69E-05	0.00069
Itga11	1.49	2.70E-05	0.00070
Use1	0.89	2.76E-05	0.00071015
Lrp5	1.11	2.79E-05	0.00071
Aifm3	1.33	2.88E-05	0.00074
Grb10	1.11	2.94E-05	0.00075
Spcs2	0.90	2.94E-05	0.00075
Gadd45gip1	0.87	2.94E-05	0.00075
Fndc3b	1.12	3.00E-05	0.00076
Taldo1	0.91	3.03E-05	0.00077
Dcps	0.91	3.04E-05	0.00077
Cox7a2	0.92	3.10E-05	0.00078
Pfdn4	0.87	3.13E-05	0.00079
Erbb4	1.19	3.15E-05	0.00079
Rpl8	0.91	3.16E-05	0.00079
Psat1	0.88	3.16E-05	0.00079
Med27	0.91	3.16E-05	0.00079
Hipk2	1.18	3.19E-05	0.00080
Zfp428	0.93	3.20E-05	0.00080
Prmt1	0.88	3.20E-05	0.00080
Mrps18a	0.88	3.22E-05	0.00080
Mien1	0.88	3.24E-05	0.00080
Edn3	1.57	3.23E-05	0.00080
Macf1	1.04	3.24E-05	0.00080
Samd8	1.06	3.28E-05	0.00081
Scyl1	0.92	3.31E-05	0.00081
Aamp	0.90	3.35E-05	0.00082
Mrpl38	0.92	3.35E-05	0.00082
Mvk	0.79	3.33E-05	0.00082

Vps4a	0.94	3.34E-05	0.00082
Setd5	1.06	3.37E-05	0.000819672
Mir331	1.55	3.41E-05	0.00083
Tuba1a	0.95	3.44E-05	0.00083
Rpl13a	0.87	3.44E-05	0.00083
Apc	1.07	3.50E-05	0.00085
Col11a1	1.14	3.52E-05	0.00085
Luzp1	1.03	3.53E-05	0.00085
Trappc3	0.96	3.57E-05	0.00086
Htt	1.00	3.57E-05	0.00086
Arf1	0.94	3.61E-05	0.000860024
Elp5	0.91	3.61E-05	0.000860024
Stip1	0.90	3.61E-05	0.000860024
Cst3	0.84	3.62E-05	0.000860024
Vps35	0.91	3.60E-05	0.000860024
Cd109	1.31	3.64E-05	0.00086
Zfp275	1.03	3.66E-05	0.00087
Slc7a2	1.13	3.66E-05	0.00087
Mageh1	0.91	3.68E-05	0.00087
Hist3h2a	0.90	3.73E-05	0.00088
Bgn	1.33	3.75E-05	0.00088
Tgfbr3	1.48	3.79E-05	0.000890365
Ptpn14	1.19	3.83E-05	0.00090
Wdr74	0.90	3.85E-05	0.00090
Clta	0.95	3.90E-05	0.00091
Actb	0.94	3.90E-05	0.00091
Atxn1	1.10	3.91E-05	0.00091
Itga2	1.52	3.94E-05	0.00091
Mib1	1.13	3.94E-05	0.00091
Lrp4	1.08	3.97E-05	0.00092
Mid1ip1	0.92	3.97E-05	0.00092
Ppfibp1	1.14	4.08E-05	0.00094
Atp13a3	1.08	4.11E-05	0.00094
Ptbp3	1.11	4.11E-05	0.00094
Ksr2	1.16	4.10E-05	0.00094
Rabgap1l	1.09	4.16E-05	0.00095
0610010K14Rik	0.95	4.15E-05	0.00095
Lama1	1.14	4.15E-05	0.00095
Alkbh6	0.87	4.15E-05	0.00095
Adamts4	1.23	4.19E-05	0.00095
Rbfox2	1.08	4.19E-05	0.00095
St8sia1	1.09	4.20E-05	0.00095
Babam1	0.90	4.26E-05	0.00096
Gpn1	0.88	4.29E-05	0.000969533
Mrpl27	0.88	4.33E-05	0.000976504
Rcl1	0.83	4.34E-05	0.00098
Mrps31	0.86	4.36E-05	0.000980284
Flnc	1.28	4.41E-05	0.00099
Elavl3	1.05	4.47E-05	0.0010

Zdhhc21	1.06	4.50E-05	0.0010
Kank2	1.21	4.51E-05	0.0010
Ank3	1.10	4.54E-05	0.0010
Glod4	0.89	4.58E-05	0.0010
Col3a1	1.80	4.59E-05	0.0010
Snrpd2	0.91	4.74E-05	0.0011
Dnajc11	0.92	4.81E-05	0.0011
Fsd1l	1.12	4.85E-05	0.0011
Tuba1b	0.94	4.88E-05	0.0011
Strbp	0.98	4.90E-05	0.0011
Ptprd	1.18	4.90E-05	0.0011
Pgls	0.89	4.96E-05	0.0011
Lrp6	1.11	4.99E-05	0.0011
Tmsb10	0.92	5.05E-05	0.0011
Fbxl20	1.06	5.10E-05	0.0011
Ftl1	0.94	5.14E-05	0.0011
Nfic	1.05	5.17E-05	0.0011
Rbms2	1.09	5.18E-05	0.0011
Rnf38	1.14	5.16E-05	0.0011
Atp6v1g1	0.96	5.16E-05	0.0011
Gm10653	0.71	5.19E-05	0.0011
Arhgap31	1.07	5.24E-05	0.0011
Tmem126a	0.94	5.23E-05	0.0011
Aarsd1	0.89	5.26E-05	0.0011
Atp6v1d	0.94	5.28E-05	0.0011
Rrp7a	0.88	5.28E-05	0.0011
Rheb	0.91	5.30E-05	0.0011
Aacs	0.84	5.29E-05	0.0011
Dtnbp1	0.91	5.33E-05	0.0011
Tufm	0.88	5.36E-05	0.0011
Armcx4	1.07	5.38E-05	0.0011
Yeats4	0.95	5.45E-05	0.0012
Pdgfrb	1.13	5.46E-05	0.0012
Frem1	1.25	5.48E-05	0.0012
Rbm19	0.81	5.47E-05	0.0012
Rps24	0.95	5.49E-05	0.0012
Txnl4b	0.81	5.51E-05	0.0012
Wars	0.91	5.58E-05	0.0012
Rc3h2	1.09	5.58E-05	0.0012
Scai	1.15	5.59E-05	0.0012
Dctn5	0.94	5.60E-05	0.0012
Eef1a1	0.93	5.63E-05	0.0012
Hipk3	1.04	5.66E-05	0.0012
Rps7	0.89	5.74E-05	0.0012
Txndc12	0.95	5.74E-05	0.0012
Miat	1.31	5.73E-05	0.0012
Prelid1	0.93	5.80E-05	0.0012
Chka	1.16	5.86E-05	0.0012
Rbmx2	0.85	5.85E-05	0.0012

Rpl10	0.96	5.88E-05	0.0012
Mical3	1.05	5.91E-05	0.0012
Bola2	0.89	5.94E-05	0.0012
Smg1	1.07	5.97E-05	0.0012
Gpx1	0.91	5.98E-05	0.0012
Wdr26	1.05	6.02E-05	0.0012
Rrp1	0.92	6.02E-05	0.0012
Snrnp25	0.83	6.05E-05	0.0012
Ap4s1	0.90	6.08E-05	0.0012
Apopt1	0.89	6.06E-05	0.0012
Nol12	0.85	6.07E-05	0.0012
Edf1	0.93	6.08E-05	0.0012
Akr1a1	0.92	6.05E-05	0.0012
Scn8a	1.03	6.14E-05	0.0012
Pgap1	1.05	6.17E-05	0.0012
D10Jhu81e	0.89	6.17E-05	0.0012
Cox7a2l	0.92	6.18E-05	0.0012
Khdrbs2	0.90	6.25E-05	0.0013
Dhcr24	0.86	6.28E-05	0.0013
Fryl	1.11	6.29E-05	0.0013
Sec24a	1.08	6.33E-05	0.0013
Suclg1	0.90	6.34E-05	0.0013
Nedd8	0.95	6.37E-05	0.0013
Acsbg1	0.75	6.41E-05	0.0013
Ovgp1	1.33	6.48E-05	0.0013
Mrpl20	0.91	6.48E-05	0.0013
Rdh14	0.91	6.52E-05	0.0013
Idh3b	0.91	6.54E-05	0.0013
Elof1	0.93	6.54E-05	0.0013
Hn1l	0.91	6.56E-05	0.0013
Tbx18	1.60	6.72E-05	0.0013
Phlpp2	1.00	6.77E-05	0.0013
Eef1d	0.88	6.79E-05	0.0013
Mrpl52	0.89	6.83E-05	0.0013
Bicc1	1.40	6.96E-05	0.0014
Rps2	0.84	6.95E-05	0.0014
Col4a3bp	1.08	6.98E-05	0.0014
Kcng3	1.53	7.03E-05	0.0014
Fbxo18	0.91	7.13E-05	0.0014
Adamts12	1.49	7.16E-05	0.0014
Lcmt1	0.92	7.19E-05	0.0014
Sec24d	1.11	7.28E-05	0.0014
Chmp3	0.90	7.41E-05	0.0014
Bbx	1.10	7.45E-05	0.0014
Tiam1	1.03	7.44E-05	0.0014
Adcy6	1.12	7.50E-05	0.0014
Wrn	1.11	7.49E-05	0.0014
Cope	0.93	7.49E-05	0.0014
D8Ertd738e	0.93	7.51E-05	0.0014

Herpud1	0.87	7.49E-05	0.0014
Cxcl12	1.17	7.59E-05	0.0015
Dcc	1.14	7.66E-05	0.0015
1110051M20Rik	0.93	7.66E-05	0.0015
Mrpl40	0.91	7.69E-05	0.0015
C330021F23Rik	0.88	7.69E-05	0.0015
Srp68	0.91	7.71E-05	0.0015
Zfp369	1.26	7.72E-05	0.0015
Myg1	0.91	7.75E-05	0.0015
Dut	0.84	7.74E-05	0.0015
Hspa9	0.90	7.84E-05	0.0015
Prrc1	1.07	7.84E-05	0.0015
Ufc1	0.92	7.88E-05	0.0015
Mrps24	0.84	7.91E-05	0.0015
Dtna	1.16	7.91E-05	0.0015
Akap13	0.98	7.89E-05	0.0015
Myo5a	1.03	8.02E-05	0.0015
Rassf8	1.25	8.08E-05	0.0015
Ppp1r8	0.91	8.11E-05	0.0015
Pcyt2	0.89	8.13E-05	0.0015
Cct8	0.91	8.18E-05	0.0015
Rps21	0.87	8.19E-05	0.0015
Rpl36al	0.95	8.29E-05	0.0015
Slc35b1	0.94	8.39E-05	0.0016
Mycbp2	1.05	8.38E-05	0.0016
Zmiz1	1.05	8.46E-05	0.0016
Loxl2	1.22	8.59E-05	0.0016
Smarcd3	0.92	8.58E-05	0.0016
Fdps	0.86	8.61E-05	0.0016
Ssr2	0.95	8.63E-05	0.0016
Lrrc18	1.73	8.68E-05	0.0016
Antxr1	1.13	8.71E-05	0.0016
Zbtb26	1.09	8.87E-05	0.0016
Col27a1	1.22	8.87E-05	0.0016
Isca2	0.93	8.92E-05	0.0016
Man2a2	1.02	8.91E-05	0.0016
Chac1	0.63	9.10E-05	0.0017
Mgea5	1.12	9.15E-05	0.0017
Lrig2	1.06	9.17E-05	0.0017
Fam162a	0.90	9.25E-05	0.0017
Rnf165	1.08	9.35E-05	0.0017
Dcakd	0.96	9.40E-05	0.0017
Rsph3a	0.88	9.40E-05	0.0017
Trp73	1.26	9.41E-05	0.0017
Tbc1d4	1.10	9.48E-05	0.0017
Adamts9	1.17	9.56E-05	0.0017
Ptges2	0.84	9.63E-05	0.0017
P4ha2	1.28	9.69E-05	0.0017
Slc8a1	1.03	9.68E-05	0.0017

Plekhg4	1.40	9.68E-05	0.0017
Mcts2	0.89	9.80E-05	0.0017
Pnpla3	1.17	9.84E-05	0.0018
Tubb4b	0.88	9.83E-05	0.0018
Col23a1	1.18	9.87E-05	0.0018
Klf12	1.12	1.01E-04	0.0018
Fbxl17	1.01	1.02E-04	0.0018
Dnajc30	0.92	1.02E-04	0.0018
Cbr1	0.97	1.02E-04	0.0018
Alx4	1.62	1.02E-04	0.0018
Swsap1	0.86	1.03E-04	0.0018
Cnot6l	1.17	1.03E-04	0.0018
Rps5	0.88	1.03E-04	0.0018
Itsn1	0.98	1.05E-04	0.0018
Pram1	1.49	1.05E-04	0.0018
Tet1	1.00	1.05E-04	0.0018
Sptbn4	1.05	1.06E-04	0.0019
Lonp2	0.92	1.06E-04	0.0019
Podxl	1.13	1.07E-04	0.0019
Cnpy2	0.94	1.08E-04	0.0019
Bop1	0.84	1.08E-04	0.0019
Csrnp3	1.06	1.09E-04	0.0019
Sestd1	1.05	1.09E-04	0.0019
Smad3	1.13	1.09E-04	0.0019
Polr2m	0.90	1.09E-04	0.0019
Noc2l	0.89	1.09E-04	0.0019
Ogt	1.22	1.10E-04	0.0019
Rasal2	1.07	1.10E-04	0.0019
Cd163	1.48	1.10E-04	0.0019
Pfkfb2	1.06	1.11E-04	0.0019
Nov	1.24	1.12E-04	0.0019
Shank2	1.06	1.12E-04	0.0019
Cplx1	0.85	1.14E-04	0.0019
Slc25a4	0.93	1.14E-04	0.0020
Pycrl	0.87	1.15E-04	0.0020
Emc4	0.93	1.15E-04	0.0020
Synj2bp	1.02	1.16E-04	0.0020
Srp9	0.95	1.16E-04	0.0020
Ndufb5	0.91	1.17E-04	0.0020
Cers6	1.12	1.18E-04	0.0020
Ank2	1.12	1.18E-04	0.0020
Ddx54	0.86	1.19E-04	0.0020
March4	1.08	1.20E-04	0.0020
Cacnb4	1.06	1.20E-04	0.0020
Pou2f1	1.06	1.21E-04	0.0020
Slc41a1	1.13	1.22E-04	0.0021
Cox16	0.84	1.22E-04	0.0021
Mrpl45	0.86	1.24E-04	0.0021
Naa10	0.89	1.25E-04	0.0021

Fez1	0.96	1.25E-04	0.0021
Abi2	1.05	1.26E-04	0.0021
Syn3	1.07	1.27E-04	0.0021
Zfp366	1.30	1.27E-04	0.0021
Alg13	1.10	1.29E-04	0.0021
Rpl27a	0.93	1.29E-04	0.0022
Rabif	0.90	1.30E-04	0.0022
Rest	1.19	1.29E-04	0.0022
Nf1	1.02	1.32E-04	0.0022
Lmo2	0.88	1.31E-04	0.0022
Nop10	0.92	1.31E-04	0.0022
Rps3	0.85	1.32E-04	0.0022
Pcolce	1.46	1.32E-04	0.0022
Mpc2	0.88	1.33E-04	0.0022
Rfx3	1.13	1.35E-04	0.0022
Nit2	0.87	1.36E-04	0.0022
Eif3i	0.89	1.36E-04	0.0022
Sipa1l3	1.02	1.36E-04	0.0022
Cab39l	0.86	1.37E-04	0.0022
0610012G03Rik	0.89	1.37E-04	0.0022
Acot10	0.90	1.38E-04	0.0023
Ccdc124	0.93	1.38E-04	0.0023
1700020I14Rik	1.19	1.39E-04	0.0023
Ipo4	0.89	1.39E-04	0.0023
Prex2	1.05	1.40E-04	0.0023
Mff	0.93	1.41E-04	0.0023
Cox10	0.85	1.41E-04	0.0023
Dock6	1.13	1.41E-04	0.0023
Anapc16	0.93	1.41E-04	0.0023
Cd2ap	1.23	1.41E-04	0.0023
Sobp	1.07	1.42E-04	0.0023
Timm13	0.86	1.42E-04	0.0023
Nans	0.88	1.43E-04	0.0023
C1qbp	0.94	1.43E-04	0.0023
Dbnl	0.90	1.44E-04	0.0023
Amot	1.08	1.44E-04	0.0023
Lancl1	0.86	1.44E-04	0.0023
Atl3	1.11	1.45E-04	0.0023
Cops3	0.92	1.46E-04	0.0023
Idh3g	0.95	1.47E-04	0.0023
Lamtor2	0.95	1.47E-04	0.0023
Nelfe	0.91	1.48E-04	0.0024
Tusc2	0.90	1.48E-04	0.0024
Marveld1	1.18	1.49E-04	0.0024
Ndufb6	0.87	1.49E-04	0.0024
Kif21b	1.03	1.50E-04	0.0024
March6	1.05	1.51E-04	0.0024
Cep350	1.04	1.52E-04	0.0024
Hecw1	1.04	1.52E-04	0.0024

Mrpl46	0.91	1.54E-04	0.0024
Rere	1.08	1.55E-04	0.0024
Coq6	0.83	1.55E-04	0.0024
D630045J12Rik	1.07	1.56E-04	0.0024
Prickle2	1.04	1.56E-04	0.0024
Smurf2	1.06	1.57E-04	0.0025
Psmc2	0.92	1.59E-04	0.0025
Atp6v1e1	0.94	1.60E-04	0.0025
Ncam1	1.08	1.60E-04	0.0025
Mlf2	0.93	1.62E-04	0.0025
Kcnc2	1.13	1.62E-04	0.0025
Adam19	1.03	1.66E-04	0.0026
Tbc1d17	0.89	1.66E-04	0.0026
Muc6	1.40	1.66E-04	0.0026
Gcsh	0.87	1.66E-04	0.0026
Slc5a3	1.07	1.66E-04	0.0026
Kcnh7	1.20	1.67E-04	0.0026
Pag1	1.06	1.68E-04	0.0026
Fcho2	1.14	1.68E-04	0.0026
Ubxn7	1.02	1.69E-04	0.0026
Nsun7	1.46	1.69E-04	0.0026
Eif5a	0.91	1.70E-04	0.0026
Ngrn	0.90	1.73E-04	0.0027
Marcksl1	0.97	1.73E-04	0.0027
Pitpnm2	1.02	1.74E-04	0.0027
Tns1	1.05	1.75E-04	0.0027
Immt	0.89	1.75E-04	0.0027
Uaca	1.14	1.77E-04	0.0027
Ankrd11	1.05	1.77E-04	0.0027
Muc1	1.42	1.80E-04	0.0027
Ap2m1	0.90	1.80E-04	0.0027
Usp31	1.01	1.80E-04	0.0027
Golgb1	1.09	1.82E-04	0.0028
Nova2	1.09	1.83E-04	0.0028
Bag1	0.93	1.84E-04	0.0028
Polr2e	0.92	1.84E-04	0.0028
Nipsnap1	0.94	1.85E-04	0.0028
Rps10	0.94	1.86E-04	0.0028
Eef1g	0.91	1.86E-04	0.0028
Aldh1a2	1.56	1.87E-04	0.0028
Mrps25	0.78	1.87E-04	0.0028
Rps23	0.92	1.88E-04	0.0028
Crabp1	0.77	1.90E-04	0.0029
Sned1	1.65	1.91E-04	0.0029
Abl2	0.99	1.93E-04	0.0029
Myt1l	1.06	1.94E-04	0.0029
Gca	1.19	1.96E-04	0.0029
Syndig1	0.81	1.96E-04	0.0029
Mecp2	1.00	1.95E-04	0.0029

Ndufa8	0.93	1.97E-04	0.0029
Ddx59	0.85	1.98E-04	0.0029
Cd46	1.62	1.99E-04	0.0030
Prdx5	0.88	2.00E-04	0.0030
Col16a1	1.29	2.00E-04	0.0030
Sae1	0.93	1.99E-04	0.0030
Hspe1	0.92	2.00E-04	0.0030
Prkce	1.03	2.01E-04	0.0030
Itga1	1.09	2.02E-04	0.0030
Tnr	1.14	2.05E-04	0.0030
Stxbp4	1.06	2.05E-04	0.0030
Mbnl2	1.13	2.07E-04	0.0030
Qdpr	0.87	2.07E-04	0.0030
Dmpk	1.23	2.07E-04	0.0030
Agfg2	0.81	2.08E-04	0.0030
Tmem8b	1.11	2.12E-04	0.0031
Fh1	0.88	2.13E-04	0.0031
Sgta	0.93	2.13E-04	0.0031
Tbc1d9b	0.89	2.13E-04	0.0031
Snrpd1	0.94	2.13E-04	0.0031
Slc38a9	1.04	2.14E-04	0.0031
Dad1	0.86	2.18E-04	0.0032
Psmd10	0.89	2.18E-04	0.0032
Trabd2b	1.59	2.19E-04	0.0032
Acadsb	1.09	2.21E-04	0.0032
Vcpip1	1.09	2.22E-04	0.0032
Rin3	1.22	2.23E-04	0.0032
Vta1	0.92	2.26E-04	0.0033
Evl	0.91	2.28E-04	0.0033
Stab1	1.32	2.27E-04	0.0033
Ndufa3	0.91	2.28E-04	0.0033
Nenf	0.92	2.29E-04	0.0033
Rims2	1.09	2.29E-04	0.0033
Skap2	0.68	2.29E-04	0.0033
Cdc42bpg	1.14	2.29E-04	0.0033
Nfatc2	1.12	2.29E-04	0.0033
Bmp7	1.31	2.30E-04	0.0033
Fam58b	0.89	2.30E-04	0.0033
F13a1	1.37	2.31E-04	0.0033
Mfsd7b	1.07	2.32E-04	0.0033
BC004004	0.87	2.32E-04	0.0033
Sfmbt1	1.02	2.33E-04	0.0033
Psmb2	0.97	2.34E-04	0.0033
Bdh1	0.90	2.37E-04	0.0034
Arhgdia	0.88	2.38E-04	0.0034
Rps29	0.88	2.38E-04	0.0034
Eml5	1.17	2.38E-04	0.0034
Mtch1	0.90	2.41E-04	0.0034
Pik3r1	1.07	2.42E-04	0.0034

Cd248	1.24	2.42E-04	0.0034
Nudc	0.97	2.42E-04	0.0034
Rplp0	0.91	2.45E-04	0.0034
Kdm2a	1.05	2.45E-04	0.0034
Otud7b	1.05	2.45E-04	0.0034
Gpatch4	0.87	2.46E-04	0.0034
Phldb1	1.06	2.47E-04	0.0035
Naa30	1.07	2.48E-04	0.0035
9130401M01Rik	0.85	2.48E-04	0.0035
Ppp1r11	0.92	2.51E-04	0.0035
Spen	1.06	2.51E-04	0.0035
Epn1	0.90	2.51E-04	0.0035
Mrps27	0.84	2.53E-04	0.0035
Abcc9	1.04	2.53E-04	0.0035
Lsm3	0.94	2.55E-04	0.0035
Xrn1	1.14	2.55E-04	0.0035
Eef2	0.94	2.56E-04	0.0036
Fstl1	1.19	2.57E-04	0.0036
4930481A15Rik	0.84	2.59E-04	0.0036
Eif3l	0.95	2.60E-04	0.0036
Atp5j2	0.84	2.62E-04	0.0036
Aak1	0.96	2.64E-04	0.0036
Cd93	1.11	2.67E-04	0.0037
Frem2	1.08	2.67E-04	0.0037
Txnip	1.19	2.66E-04	0.0037
Chd9	1.04	2.67E-04	0.0037
Evi5	1.11	2.74E-04	0.0038
Erp29	0.95	2.75E-04	0.0038
Tmem183a	0.91	2.78E-04	0.0038
Cacna1d	1.11	2.79E-04	0.0038
Zbtb41	1.03	2.80E-04	0.0038
Myl6	0.91	2.81E-04	0.0038
Ptpro	1.06	2.82E-04	0.0038
Ppm1e	1.06	2.83E-04	0.0039
Eci1	0.85	2.84E-04	0.0039
Sp140	1.50	2.86E-04	0.0039
Cdc14b	1.18	2.86E-04	0.0039
Nbea	1.09	2.87E-04	0.0039
Tet3	1.06	2.88E-04	0.0039
Ube2v1	0.88	2.90E-04	0.0039
Mrps30	0.92	2.91E-04	0.0039
Gps1	0.89	2.93E-04	0.0040
Tmc7	1.11	2.93E-04	0.0040
Braf	1.06	2.94E-04	0.0040
Fam135b	1.14	2.95E-04	0.0040
Lnpep	1.14	2.96E-04	0.0040
Ccdc28b	0.93	2.97E-04	0.0040
Etnk1	1.10	2.98E-04	0.0040
Igdcc3	1.14	2.98E-04	0.0040

Celsr3	1.09	2.98E-04	0.0040
Got1	0.85	2.99E-04	0.0040
Efemp1	1.57	3.03E-04	0.0041
Vangl2	1.12	3.04E-04	0.0041
Mrgprf	1.73	3.07E-04	0.0041
Cwc15	0.94	3.08E-04	0.0041
Gjb6	1.52	3.08E-04	0.0041
Pcgf6	1.18	3.11E-04	0.0041
Aldoc	0.75	3.11E-04	0.0041
Cdh4	1.06	3.16E-04	0.0042
Zeb1	1.08	3.18E-04	0.0042
Eri3	0.90	3.18E-04	0.0042
Cyc1	0.86	3.20E-04	0.0042
1110004E09Rik	0.92	3.21E-04	0.0042
Lamtor4	0.89	3.23E-04	0.0043
Lama2	1.14	3.23E-04	0.0043
Map3k9	1.01	3.25E-04	0.0043
Acr	1.34	3.26E-04	0.0043
Ubl5	0.93	3.27E-04	0.0043
Zbtb7a	1.05	3.27E-04	0.0043
Pno1	0.89	3.28E-04	0.0043
Tenm2	1.13	3.28E-04	0.0043
Myo18a	1.00	3.28E-04	0.0043
Pxn	1.15	3.28E-04	0.0043
Postn	1.38	3.29E-04	0.0043
N6amt1	0.85	3.31E-04	0.0043
Tle2	1.15	3.32E-04	0.0043
Zzef1	1.01	3.33E-04	0.0043
Bach2	1.15	3.35E-04	0.0044
Atp5k	0.86	3.36E-04	0.0044
Pdzd7	1.17	3.37E-04	0.0044
Kcnq1ot1	1.18	3.40E-04	0.0044
Zdhhc20	1.11	3.41E-04	0.0044
Nrp2	1.07	3.45E-04	0.0045
Cby1	0.96	3.45E-04	0.0045
Ybx1	0.93	3.45E-04	0.0045
2210016L21Rik	0.93	3.45E-04	0.0045
Baz2a	1.05	3.46E-04	0.0045
Maf	1.18	3.47E-04	0.0045
Wnk4	1.23	3.51E-04	0.0045
Abhd12	0.88	3.55E-04	0.0046
Capzb	0.92	3.55E-04	0.0046
Tgfbr2	1.16	3.54E-04	0.0046
Gabrb2	1.09	3.59E-04	0.0046
Magi3	1.05	3.60E-04	0.0046
Mpdz	1.05	3.60E-04	0.0046
9330159F19Rik	1.11	3.64E-04	0.0046
Celf5	1.10	3.64E-04	0.0046
Zfp277	0.84	3.64E-04	0.0046

Lonp1	0.90	3.63E-04	0.0046
Hspbp1	0.86	3.63E-04	0.0046
Fam199x	1.05	3.62E-04	0.0046
Synpo	1.10	3.71E-04	0.0047
4732471J01Rik	1.23	3.73E-04	0.0047
Exog	0.88	3.72E-04	0.0047
Rpp40	0.70	3.73E-04	0.0047
Ppa2	0.88	3.74E-04	0.0047
Six5	1.22	3.74E-04	0.0047
Cpd	1.05	3.75E-04	0.0047
Plod1	1.15	3.75E-04	0.0047
Atg3	0.89	3.77E-04	0.0048
Cops8	0.93	3.79E-04	0.0048
Mettl22	0.88	3.79E-04	0.0048
Gja5	1.43	3.79E-04	0.0048
Cldn19	1.32	3.78E-04	0.0048
Minos1	0.88	3.78E-04	0.0048
Snx17	0.88	3.82E-04	0.0048
Gstm5	0.91	3.83E-04	0.0048
Dda1	0.91	3.84E-04	0.0048
Pank1	0.83	3.85E-04	0.0048
Bpgm	0.90	3.88E-04	0.0048
Rreb1	1.12	3.90E-04	0.0049
4833422C13Rik	1.21	3.89E-04	0.0049
Pkd1	0.98	3.90E-04	0.0049
Plagl2	1.12	3.89E-04	0.0049
Gbp9	1.40	3.91E-04	0.0049
Copz1	0.91	3.94E-04	0.0049
Mgst3	0.85	3.97E-04	0.0049
Grwd1	0.83	3.98E-04	0.0049
Osbpl6	1.03	4.00E-04	0.0050
Mars	0.88	4.00E-04	0.0050
Txn1	0.92	4.00E-04	0.0050
Golga4	1.11	4.01E-04	0.0050
Atp5f1	0.92	4.03E-04	0.0050
Anapc5	0.92	4.03E-04	0.0050
Tmem143	0.83	4.03E-04	0.0050
Qk	1.03	4.06E-04	0.0050
Heph	1.17	4.06E-04	0.0050
Atp6v1f	0.90	4.06E-04	0.0050
Tjp1	1.10	4.07E-04	0.0050
Arid1b	0.98	4.11E-04	0.0050
Dmwd	0.92	4.11E-04	0.0050
Aff4	1.05	4.15E-04	0.0051
Pdp1	0.91	4.15E-04	0.0051
Cetn2	0.95	4.15E-04	0.0051
Dync1h1	1.04	4.17E-04	0.0051
Tbc1d2b	1.02	4.17E-04	0.0051
Atp5h	0.92	4.19E-04	0.0051

Hspa8	0.90	4.23E-04	0.0051
Lamb1	1.15	4.24E-04	0.0052
Chrnb1	1.40	4.28E-04	0.0052
Fth1	0.93	4.30E-04	0.0052
Zfp36l2	1.12	4.31E-04	0.0052
Ndufa7	0.87	4.32E-04	0.0052
Ssna1	0.91	4.32E-04	0.0052
Hddc2	0.91	4.35E-04	0.0053
Bola1	0.84	4.35E-04	0.0053
Apbb2	1.04	4.39E-04	0.0053
Kat5	0.92	4.41E-04	0.0053
Ppp2r1a	0.91	4.42E-04	0.0053
D130040H23Rik	1.26	4.42E-04	0.0053
Dclk1	0.98	4.43E-04	0.0053
Pik3r3	1.06	4.44E-04	0.0053
Mpnd	0.89	4.46E-04	0.0054
Slc24a5	1.20	4.46E-04	0.0054
Cald1	1.14	4.49E-04	0.0054
Rab5c	0.94	4.50E-04	0.0054
Abcc1	1.03	4.50E-04	0.0054
A2m	1.23	4.51E-04	0.0054
Zfp654	1.10	4.54E-04	0.0054
Sar1b	0.93	4.57E-04	0.0054
Slc25a11	0.88	4.56E-04	0.0054
Idi1	0.83	4.57E-04	0.0054
Gabpb2	1.11	4.57E-04	0.0054
Nr2c2	1.01	4.56E-04	0.0054
Fat1	1.08	4.55E-04	0.0054
Plagl1	1.20	4.59E-04	0.0054
Adam9	1.09	4.59E-04	0.0054
1110032A03Rik	0.87	4.59E-04	0.0054
Fkbp8	0.89	4.61E-04	0.0055
Brd4	1.06	4.62E-04	0.0055
Ston1	1.24	4.64E-04	0.0055
Mpv17l2	0.87	4.65E-04	0.0055
Pygo1	1.16	4.66E-04	0.0055
Prmt2	0.88	4.67E-04	0.0055
A330076H08Rik	1.42	4.71E-04	0.0055
Xkr4	1.17	4.72E-04	0.0055
Aes	0.92	4.72E-04	0.0055
Trim67	1.02	4.71E-04	0.0055
Fzd7	1.13	4.73E-04	0.0055
Trrap	1.04	4.74E-04	0.0055
Chl1	1.11	4.73E-04	0.0055
Dip2b	1.04	4.76E-04	0.0056
Fnip2	1.01	4.76E-04	0.0056
Arid2	1.09	4.77E-04	0.0056
Uvssa	1.15	4.78E-04	0.0056
Orc3	1.04	4.78E-04	0.0056

Rsf1	1.04	4.79E-04	0.0056
Dhtkd1	1.19	4.81E-04	0.0056
Acvr2a	1.05	4.83E-04	0.0056
Tspan18	1.15	4.83E-04	0.0056
Ap2s1	0.92	4.84E-04	0.0056
Megf8	1.06	4.85E-04	0.0056
Mdh1	0.88	4.86E-04	0.0056
Arhgap29	1.23	4.86E-04	0.0056
Rpl4	0.95	4.87E-04	0.0056
Mtss1	1.07	4.90E-04	0.0057
Sppl2a	1.06	4.92E-04	0.0057
Hibch	0.83	4.93E-04	0.0057
Nudt15	0.71	4.94E-04	0.0057
S1pr3	1.08	4.96E-04	0.0057
Strip1	0.89	4.96E-04	0.0057
Nudt2	0.90	4.96E-04	0.0057
Eefsec	0.87	4.96E-04	0.0057
Arl15	1.08	5.04E-04	0.0058
Aco2	0.91	5.03E-04	0.0058
Srgap2	1.02	5.06E-04	0.0058
Psme4	1.13	5.08E-04	0.0058
Tmem97	0.84	5.11E-04	0.0058
Tmtc1	1.09	5.11E-04	0.0058
Dgki	1.10	5.15E-04	0.0059
Ash1l	1.09	5.20E-04	0.0059
Higd2a	0.95	5.23E-04	0.0060
Slc43a2	1.08	5.23E-04	0.0060
Rbm15	1.16	5.24E-04	0.0060
Fnbp4	1.13	5.26E-04	0.0060
Stmn3	0.91	5.28E-04	0.0060
Psmd5	0.92	5.30E-04	0.0060
1110008F13Rik	0.95	5.30E-04	0.0060
Dcaf17	1.03	5.36E-04	0.0061
Clasp1	1.03	5.39E-04	0.0061
Arih1	1.03	5.39E-04	0.0061
Ptp4a3	0.85	5.41E-04	0.0061
Med13	1.10	5.41E-04	0.0061
Xbp1	0.88	5.44E-04	0.0061
Mkrn2	0.89	5.45E-04	0.0061
Psmb10	0.81	5.45E-04	0.0061
Cpsf4l	1.38	5.47E-04	0.0062
Ydjc	0.86	5.53E-04	0.0062
Ccdc38	1.37	5.55E-04	0.0062
Pomp	0.97	5.55E-04	0.0062
Ccser2	1.02	5.62E-04	0.0063
Vps13c	1.04	5.62E-04	0.0063
Rai1	1.03	5.66E-04	0.0063
Fkbp10	1.24	5.68E-04	0.0063
Sidt1	1.24	5.67E-04	0.0063

Vav3	1.11	5.67E-04	0.0063
Mrps17	0.95	5.69E-04	0.0063
Commd1	0.90	5.70E-04	0.0064
Rab8a	0.93	5.71E-04	0.0064
Rpl36a	0.89	5.74E-04	0.0064
Cpeb4	1.14	5.78E-04	0.0064
Ptgfrn	1.04	5.78E-04	0.0064
Dnaja2	0.93	5.78E-04	0.0064
Daxx	0.89	5.88E-04	0.0065
Marc2	0.85	5.92E-04	0.0065
2010107E04Rik	0.91	5.92E-04	0.0065
Ranbp10	1.06	5.92E-04	0.0065
Cep164	1.10	5.92E-04	0.0065
Pdha1	0.89	5.94E-04	0.0066
Actr1a	0.92	5.96E-04	0.0066
Rnf6	1.11	5.98E-04	0.0066
Xpo1	1.10	5.99E-04	0.0066
Chchd4	0.93	5.99E-04	0.0066
Plrg1	0.91	6.00E-04	0.0066
Metrn	0.82	6.01E-04	0.0066
Arl2	0.89	6.03E-04	0.0066
Mdp1	0.91	6.05E-04	0.0066
Piezo1	1.08	6.10E-04	0.0067
Ap1s1	0.90	6.10E-04	0.0067
Apaf1	1.03	6.12E-04	0.0067
Ntmt1	0.92	6.16E-04	0.0067
Heg1	1.17	6.18E-04	0.0067
Rpl6	0.93	6.19E-04	0.0068
Ankrd12	1.09	6.25E-04	0.0068
Ran	0.89	6.24E-04	0.0068
Skor1	0.52	6.26E-04	0.0068
Aagab	0.89	6.26E-04	0.0068
Anapc11	0.82	6.27E-04	0.0068
Cog4	0.87	6.27E-04	0.0068
Pbx1	1.08	6.31E-04	0.0068
Frmd5	1.15	6.32E-04	0.0068
Gm8773	0.83	6.35E-04	0.0069
Ssh2	1.02	6.37E-04	0.0069
Cflar	1.04	6.39E-04	0.0069
Cyp4f17	1.24	6.44E-04	0.0070
Triobp	1.04	6.46E-04	0.0070
Cyp2d22	1.35	6.46E-04	0.0070
Mrps18b	0.89	6.50E-04	0.0070
2310009A05Rik	0.86	6.50E-04	0.0070
Plod2	1.13	6.50E-04	0.0070
Net1	1.12	6.52E-04	0.0070
Polr2f	0.91	6.54E-04	0.0070
Timmdc1	0.89	6.54E-04	0.0070
Setx	1.06	6.54E-04	0.0070

Zfp697	1.06	6.54E-04	0.0070
Lsm6	0.86	6.53E-04	0.0070
Slc22a6	1.76	6.56E-04	0.0070
Uvrag	0.89	6.58E-04	0.0070
Tma7	0.93	6.58E-04	0.0070
Slc4a8	1.04	6.60E-04	0.0070
Pop5	0.90	6.62E-04	0.0071
Slc6a8	1.10	6.66E-04	0.0071
Ndufs7	0.90	6.68E-04	0.0071
Cacna1h	1.05	6.72E-04	0.0071
Nbeal1	1.11	6.73E-04	0.0072
Stox2	1.01	6.76E-04	0.0072
Eif2ak2	1.20	6.78E-04	0.0072
1110004F10Rik	0.97	6.78E-04	0.0072
Ggh	0.87	6.79E-04	0.0072
Rbm47	1.30	6.80E-04	0.0072
Slc35g1	1.25	6.81E-04	0.0072
Hmbox1	1.15	6.84E-04	0.0072
Dctpp1	0.88	6.85E-04	0.0072
Ttc27	0.83	6.87E-04	0.0072
Zfp827	1.05	6.87E-04	0.0072
Zfp445	1.09	6.87E-04	0.0072
Reck	1.17	6.94E-04	0.0073
Klf4	1.14	6.95E-04	0.0073
Lap3	0.87	6.99E-04	0.0073
Rhot1	0.89	7.02E-04	0.0074
Slc26a2	1.14	7.08E-04	0.0074
Cped1	1.46	7.08E-04	0.0074
Nudt14	0.87	7.09E-04	0.0074
Tcf7l1	1.13	7.09E-04	0.0074
Fis1	0.90	7.11E-04	0.0074
Atp6v1h	0.93	7.13E-04	0.0074
Scand1	0.91	7.14E-04	0.0074
Gm10548	0.71	7.17E-04	0.0075
Rab30	1.26	7.22E-04	0.0075
Baz2b	1.10	7.24E-04	0.0075
Foxl2os	1.51	7.24E-04	0.0075
Tenm3	1.09	7.28E-04	0.0076
Atp5g1	0.87	7.33E-04	0.0076
Lats1	1.04	7.34E-04	0.0076
Med19	0.91	7.34E-04	0.0076
Thoc6	0.85	7.35E-04	0.0076
Bace1	1.05	7.39E-04	0.0077
Pitpnc1	1.04	7.46E-04	0.0077
Fry	1.05	7.50E-04	0.0078
Klhdc9	0.90	7.53E-04	0.0078
Slc16a12	1.25	7.52E-04	0.0078
Ldhb	0.94	7.53E-04	0.0078
Nap1l4	0.90	7.53E-04	0.0078

Myo9a	1.02	7.51E-04	0.0078
Rab35	0.96	7.56E-04	0.0078
Senp5	1.01	7.57E-04	0.0078
Cdc37	0.90	7.58E-04	0.0078
Arid1a	1.05	7.62E-04	0.0078
6330403K07Rik	0.94	7.62E-04	0.0078
Ero1l	1.12	7.64E-04	0.0078
Slc22a15	1.02	7.64E-04	0.0078
Ddx28	0.89	7.64E-04	0.0078
Cep97	1.10	7.67E-04	0.0078
Mcee	0.87	7.67E-04	0.0078
Bsg	0.91	7.69E-04	0.0078
Lyve1	1.45	7.69E-04	0.0078
Mrps11	0.92	7.71E-04	0.0079
Brf2	0.90	7.70E-04	0.0079
Rccd1	1.13	7.72E-04	0.0079
Amacr	0.78	7.75E-04	0.0079
Gm5148	0.92	7.78E-04	0.0079
Bbip1	0.92	7.80E-04	0.0079
Onecut2	1.05	7.81E-04	0.0079
Ppie	0.86	7.81E-04	0.0079
Gpx4	0.88	7.82E-04	0.0079
Sssca1	0.91	7.83E-04	0.0079
Dyrk3	0.89	7.86E-04	0.0080
Adamts18	1.11	7.87E-04	0.0080
Wdr48	0.91	7.88E-04	0.0080
Gtf3c6	0.95	7.89E-04	0.0080
Chmp6	0.89	7.90E-04	0.0080
Zmynd19	0.95	7.91E-04	0.0080
Snrnp27	0.96	7.92E-04	0.0080
Lrrc59	0.94	7.96E-04	0.0080
Rgs9	1.11	7.94E-04	0.0080
Itgb1bp1	0.87	7.97E-04	0.0080
Actc1	0.77	7.97E-04	0.0080
Fkbp1a	0.97	7.97E-04	0.0080
Ppm1g	0.93	7.97E-04	0.0080
Ttc9b	0.88	7.96E-04	0.0080
N4bp1	1.03	7.99E-04	0.0080
Ubr3	1.05	8.03E-04	0.0080
Tmem132b	1.01	8.06E-04	0.0081
2610301B20Rik	0.92	8.09E-04	0.0081
Btbd2	0.89	8.11E-04	0.0081
Nrcam	1.07	8.12E-04	0.0081
Mrpl19	0.83	8.13E-04	0.0081
Marveld3	1.36	8.13E-04	0.0081
Gid8	0.93	8.14E-04	0.0081
Mtmr12	1.09	8.15E-04	0.0081
Hic1	1.23	8.19E-04	0.0081
Tcp11l1	1.12	8.20E-04	0.0081

Acvr1c	1.22	8.21E-04	0.0081
Snapc2	0.89	8.23E-04	0.0082
Sec22a	0.84	8.24E-04	0.0082
Farsa	0.91	8.25E-04	0.0082
Lrp8	1.06	8.26E-04	0.0082
Cisd1	0.91	8.32E-04	0.0082
Zfp622	0.88	8.34E-04	0.0082
Arl5b	1.11	8.45E-04	0.0083
Tmem160	0.84	8.44E-04	0.0083
Cgnl1	1.11	8.46E-04	0.0083
Kcnu1	0.76	8.53E-04	0.0084
Clasp2	1.05	8.59E-04	0.0085
4933439C10Rik	1.27	8.60E-04	0.0085
Osr1	1.54	8.61E-04	0.0085
Hivep2	1.07	8.64E-04	0.0085
Rwdd2b	0.86	8.64E-04	0.0085
D930016D06Rik	1.16	8.63E-04	0.0085
Trappc4	0.90	8.63E-04	0.0085
Cnnm4	1.07	8.66E-04	0.0085
Pde5a	1.12	8.65E-04	0.0085
Gcat	0.71	8.67E-04	0.0085
Gpr180	0.85	8.69E-04	0.0085
Tagln3	0.95	8.69E-04	0.0085
Wnk3	1.02	8.68E-04	0.0085
Aebp2	1.01	8.70E-04	0.0085
Btbd3	1.08	8.72E-04	0.0085
Dsp	1.26	8.75E-04	0.0085
Nipsnap3b	0.92	8.79E-04	0.0085
Rps16	0.90	8.81E-04	0.0086
Psmd1	0.93	8.82E-04	0.0086
Mpzl2	1.66	8.86E-04	0.0086
Dhcr7	0.88	8.87E-04	0.0086
Tango2	0.88	8.90E-04	0.0086
Rps20	0.94	8.93E-04	0.0086
Klhl34	1.22	8.97E-04	0.0087
Nudt16l1	0.92	8.98E-04	0.0087
Lrrc73	0.85	9.07E-04	0.0088
Cfdp1	0.95	9.09E-04	0.0088
Cubn	1.13	9.15E-04	0.0088
Abcb8	0.83	9.18E-04	0.0088
Camsap2	1.03	9.21E-04	0.0089
Akap11	1.03	9.21E-04	0.0089
Mdm4	1.20	9.22E-04	0.0089
Ntpcr	0.86	9.24E-04	0.0089
Wrb	0.89	9.27E-04	0.0089
Afap1	1.04	9.28E-04	0.0089
Ptpn13	1.06	9.28E-04	0.0089
Eid1	0.93	9.30E-04	0.0089
Schip1	0.80	9.30E-04	0.0089

Auts2	1.12	9.30E-04	0.0089
Snf8	0.93	9.31E-04	0.0089
Klhdc2	0.96	9.32E-04	0.0089
Kirrel	1.07	9.38E-04	0.0090
Ankrd17	1.02	9.40E-04	0.0090
Mbnl3	1.24	9.42E-04	0.0090
Atp1a2	1.15	9.47E-04	0.0090
Anapc13	0.92	9.53E-04	0.0091
Rps17	0.92	9.63E-04	0.0092
Stk25	0.89	9.67E-04	0.0092
Snupn	0.90	9.67E-04	0.0092
Setd7	1.12	9.69E-04	0.0092
Mbd6	1.18	9.71E-04	0.0092
Lama4	1.07	9.74E-04	0.0092
Ubxn6	0.93	9.80E-04	0.0093
Atp7a	1.10	9.87E-04	0.0093
Sucla2	0.89	9.89E-04	0.0094
Mrpl37	0.91	9.92E-04	0.0094
Rnf150	1.00	9.92E-04	0.0094
Itpripl2	1.13	9.99E-04	0.0094
Hyal1	1.32	9.99E-04	0.0094
H2-Ke6	0.93	1.01E-03	0.0095
Cox5a	0.91	1.01E-03	0.0095
Kcnk2	1.05	1.01E-03	0.0095
Slc9a5	1.07	1.01E-03	0.0095
0610009B22Rik	0.94	1.02E-03	0.0096
Tmtc3	1.09	1.02E-03	0.0096
Tmem222	0.89	1.02E-03	0.0096
Lrp11	0.90	1.03E-03	0.0096
Gls	1.09	1.03E-03	0.0096
Mob4	0.92	1.03E-03	0.0096
AW209491	0.89	1.03E-03	0.0096
Olfml1	1.36	1.03E-03	0.0096
Haus7	0.91	1.03E-03	0.0096
Tbc1d8	1.06	1.03E-03	0.0096
Zfp174	1.03	1.03E-03	0.0096
Cdk12	1.07	1.04E-03	0.0096
Sdf2l1	0.84	1.04E-03	0.0097
Lrrc7	1.13	1.04E-03	0.0097
Mrps34	0.96	1.04E-03	0.0097
Oscp1	0.88	1.05E-03	0.0097
Nedd4l	1.05	1.05E-03	0.0097
Sra1	0.94	1.05E-03	0.0097
Gtf2a1	1.07	1.05E-03	0.0098
Gdpd5	1.13	1.05E-03	0.0098
Zfp142	1.02	1.05E-03	0.0098
Apod	1.34	1.06E-03	0.0098
Vps51	0.87	1.06E-03	0.0098
Tnrc6a	1.05	1.06E-03	0.0098

Pbxip1	1.12	1.06E-03	0.0098
Tmem245	1.06	1.06E-03	0.0098
Maml3	1.06	1.06E-03	0.0098
Taf10	0.90	1.06E-03	0.0098
Pkig	0.93	1.06E-03	0.0098
Cript	0.96	1.07E-03	0.0098
Notch2	1.11	1.07E-03	0.0098
Bmpr2	1.17	1.07E-03	0.0098
Igdcc4	1.02	1.07E-03	0.0098
Pgs1	0.84	1.08E-03	0.0099
Klhdc7a	1.37	1.08E-03	0.0099
Hddc3	0.88	1.08E-03	0.0099
Dpp4	1.28	1.08E-03	0.0099
Elac1	1.10	1.08E-03	0.0099
4933417G07Rik	0.89	1.08E-03	0.0099
Ddx39b	0.94	1.08E-03	0.0099
BC030336	1.05	1.08E-03	0.0099
Lsm1	0.84	1.09E-03	0.0099
Slco5a1	1.14	1.09E-03	0.0099
Tfcp2l1	1.18	1.09E-03	0.0099
Itpr3	1.22	1.09E-03	0.010
Mrpl10	0.91	1.10E-03	0.010
Chd7	1.06	1.10E-03	0.010
Tbc1d24	1.03	1.10E-03	0.010
Thyn1	0.91	1.10E-03	0.010
Echs1	0.88	1.11E-03	0.010
Myh10	1.05	1.12E-03	0.010
Prr14l	1.04	1.12E-03	0.010
Dync2h1	1.03	1.12E-03	0.010
Rpl31	0.89	1.13E-03	0.010
Elk4	1.18	1.14E-03	0.010
Uap1	0.89	1.14E-03	0.010
Thg1l	0.87	1.14E-03	0.010
Med9	0.87	1.13E-03	0.010
Get4	0.87	1.14E-03	0.010
Zfp449	1.11	1.14E-03	0.010
AI504432	1.12	1.14E-03	0.010
Csnk1g2	0.93	1.15E-03	0.010
Rimbp3	1.09	1.15E-03	0.010
Tnik	1.05	1.15E-03	0.010
Ccdc8	1.14	1.16E-03	0.010
Rpl19	0.94	1.16E-03	0.010
Nars	0.89	1.16E-03	0.010
H2afj	0.90	1.16E-03	0.010
Npr2	1.17	1.16E-03	0.010
C3	1.53	1.17E-03	0.010
Sfmbt2	1.13	1.17E-03	0.010
Prps1	0.89	1.17E-03	0.010
Rpl36	0.91	1.17E-03	0.010

Mapk4	1.08	1.17E-03	0.010
Wdr34	0.89	1.17E-03	0.010
Tmem87b	1.07	1.17E-03	0.010
Atp5o	0.93	1.17E-03	0.010
Zfhx2	1.08	1.18E-03	0.010
Tomm22	0.96	1.18E-03	0.011
Prrc2c	1.11	1.19E-03	0.011
Zfp111	1.06	1.19E-03	0.011
Zfp558	1.10	1.18E-03	0.011
Tm2d3	0.92	1.19E-03	0.011
Chrd	1.18	1.19E-03	0.011
Mrpl3	0.95	1.19E-03	0.011
Sbsn	1.26	1.20E-03	0.011
Mical2	1.10	1.20E-03	0.011
Gatsl2	1.10	1.20E-03	0.011
Ttc1	0.96	1.21E-03	0.011
Emc10	0.89	1.21E-03	0.011
Ecsit	0.91	1.21E-03	0.011
Gipc1	0.93	1.21E-03	0.011
Ndrg1	1.14	1.22E-03	0.011
Egln2	0.90	1.22E-03	0.011
Yif1b	0.86	1.22E-03	0.011
Rad23a	0.92	1.23E-03	0.011
Cbx7	1.17	1.23E-03	0.011
Spop	1.08	1.24E-03	0.011
Tomm7	0.89	1.24E-03	0.011
Gm5617	0.77	1.24E-03	0.011
Wipf3	1.12	1.24E-03	0.011
Sorbs3	1.14	1.24E-03	0.011
Synm	1.02	1.25E-03	0.011
Reep2	0.92	1.25E-03	0.011
Svep1	1.27	1.25E-03	0.011
Fbln5	1.29	1.25E-03	0.011
Faf1	0.88	1.25E-03	0.011
Atpif1	0.94	1.25E-03	0.011
Zfp592	1.00	1.25E-03	0.011
Dhrs13	0.89	1.26E-03	0.011
Pak1ip1	0.91	1.25E-03	0.011
Itgbl1	1.23	1.26E-03	0.011
Phf21a	1.11	1.26E-03	0.011
H19	1.36	1.26E-03	0.011
Prrg4	1.43	1.26E-03	0.011
AU021092	0.84	1.27E-03	0.011
Bag3	1.20	1.27E-03	0.011
Manf	0.91	1.27E-03	0.011
Traf3ip2	1.15	1.27E-03	0.011
BC051226	0.90	1.27E-03	0.011
Hagh	0.89	1.28E-03	0.011
S100a16	0.87	1.28E-03	0.011

Nudt9	0.94	1.30E-03	0.011
Chd6	1.05	1.30E-03	0.011
Clip1	1.08	1.32E-03	0.011
Ank1	0.99	1.32E-03	0.011
Zmym5	1.02	1.32E-03	0.011
Trappc2l	0.93	1.32E-03	0.011
Sema3f	1.14	1.32E-03	0.011
Pld4	1.30	1.33E-03	0.011
2410015M20Rik	0.90	1.33E-03	0.011
9130011E15Rik	0.88	1.34E-03	0.011
Zfp629	1.03	1.34E-03	0.011
Znrf1	1.03	1.34E-03	0.011
Unc5b	1.08	1.35E-03	0.012
Eif2b4	0.92	1.35E-03	0.012
Trove2	1.08	1.35E-03	0.012
Lsm7	0.90	1.35E-03	0.012
Ptk7	1.11	1.35E-03	0.012
Ilk	0.95	1.36E-03	0.012
Med29	0.85	1.37E-03	0.012
Pspc1	0.92	1.37E-03	0.012
Rev3l	1.04	1.38E-03	0.012
Cdipt	0.88	1.38E-03	0.012
Vps45	0.90	1.38E-03	0.012
Snx3	0.94	1.38E-03	0.012
Reps2	1.08	1.39E-03	0.012
Cdc42	0.92	1.39E-03	0.012
Zfp169	1.09	1.40E-03	0.012
Mcam	1.19	1.41E-03	0.012
Arl2bp	0.93	1.41E-03	0.012
Nip7	0.87	1.41E-03	0.012
Rps8	0.94	1.41E-03	0.012
Arf2	0.90	1.42E-03	0.012
Dvl3	1.09	1.42E-03	0.012
Nfkbib	0.87	1.42E-03	0.012
Atp11a	1.04	1.42E-03	0.012
Bptf	1.04	1.42E-03	0.012
Pim3	0.83	1.42E-03	0.012
Dennd4c	1.07	1.42E-03	0.012
Kcnn3	1.06	1.43E-03	0.012
Psmd9	0.87	1.43E-03	0.012
Bccip	0.95	1.44E-03	0.012
Fbll1	0.88	1.44E-03	0.012
Fbln2	1.10	1.44E-03	0.012
Dbi	0.96	1.45E-03	0.012
Pgrmc2	0.94	1.45E-03	0.012
Zmym4	1.02	1.46E-03	0.012
Tmem178b	1.17	1.46E-03	0.012
2210016F16Rik	0.87	1.46E-03	0.012
Ifrd1	0.92	1.46E-03	0.012

Atrn	1.02	1.47E-03	0.012
Pdcd5	0.91	1.47E-03	0.012
Sdhd	0.89	1.47E-03	0.012
Agpat5	0.91	1.47E-03	0.012
Papd5	1.08	1.47E-03	0.012
Slc6a20a	1.59	1.48E-03	0.012
Heca	1.11	1.48E-03	0.012
Mrps23	0.95	1.48E-03	0.012
Accs	1.29	1.48E-03	0.012
Arhgap32	1.00	1.49E-03	0.012
Lamp2	1.07	1.49E-03	0.012
Pcdhga7	1.08	1.50E-03	0.012
Tsc1	0.99	1.50E-03	0.012
Osbpl3	1.08	1.50E-03	0.012
Pex19	0.90	1.51E-03	0.012
Ralgapa1	1.06	1.51E-03	0.012
Ahrr	1.22	1.51E-03	0.012
Dab2	1.26	1.51E-03	0.012
Scg5	0.96	1.51E-03	0.012
Ptpmt1	0.92	1.51E-03	0.013
Zkscan8	1.13	1.51E-03	0.013
Trim2	1.00	1.52E-03	0.013
Med21	0.93	1.52E-03	0.013
Lamtor5	0.96	1.53E-03	0.013
Mier1	1.07	1.53E-03	0.013
Tnip1	0.83	1.53E-03	0.013
Daam1	1.07	1.53E-03	0.013
Poldip2	0.93	1.54E-03	0.013
Fopnl	0.94	1.54E-03	0.013
Ptprt	1.07	1.54E-03	0.013
Lcorl	1.01	1.54E-03	0.013
Ggct	0.82	1.54E-03	0.013
Rpl39	0.88	1.54E-03	0.013
Mga	1.03	1.55E-03	0.013
Rps11	0.97	1.55E-03	0.013
Vdac3	0.91	1.55E-03	0.013
Pcmtd2	1.12	1.55E-03	0.013
Ncan	1.03	1.56E-03	0.013
Tars2	0.85	1.56E-03	0.013
Vac14	0.90	1.56E-03	0.013
Dap3	0.87	1.58E-03	0.013
Timm50	0.89	1.59E-03	0.013
Fam126b	1.02	1.59E-03	0.013
Cdk13	1.08	1.60E-03	0.013
Ccdc141	1.06	1.60E-03	0.013
Uqcrh	0.95	1.61E-03	0.013
Mrpl18	0.94	1.61E-03	0.013
Zfhx4	1.11	1.61E-03	0.013
Rab1b	0.89	1.62E-03	0.013

Apbb1	0.87	1.63E-03	0.013
Usp9x	1.03	1.63E-03	0.013
Ctbp1	0.90	1.63E-03	0.013
Mbd3	0.91	1.63E-03	0.013
Rnd2	0.94	1.64E-03	0.013
Fau	0.92	1.64E-03	0.013
Atp5d	0.94	1.64E-03	0.013
Nrbp1	0.93	1.64E-03	0.013
N4bp2	1.10	1.64E-03	0.013
Upf3a	0.93	1.64E-03	0.013
Asnsd1	0.90	1.65E-03	0.013
Ncor1	1.01	1.65E-03	0.013
Mdga2	1.04	1.65E-03	0.013
Plekhh1	1.06	1.65E-03	0.013
Myo10	1.00	1.65E-03	0.013
Tirap	1.11	1.66E-03	0.013
Rnf19b	0.88	1.66E-03	0.013
Pard6a	0.88	1.67E-03	0.013
Tmsb4x	0.96	1.67E-03	0.013
Alyref2	0.95	1.67E-03	0.013
Exosc5	0.85	1.68E-03	0.013
Arl8a	0.97	1.68E-03	0.013
Acvr1b	1.08	1.68E-03	0.013
Ids	1.08	1.68E-03	0.013
Ehd1	0.92	1.69E-03	0.014
Psmd7	0.95	1.69E-03	0.014
Kcne1l	0.90	1.70E-03	0.014
Trmt112	0.93	1.70E-03	0.014
Zc3h7a	1.15	1.70E-03	0.014
Zc3h12b	1.17	1.70E-03	0.014
Nktr	1.13	1.71E-03	0.014
Appl1	1.04	1.71E-03	0.014
Zfp609	1.06	1.71E-03	0.014
Snap23	1.15	1.72E-03	0.014
Pikfyve	1.03	1.72E-03	0.014
Ptrhd1	0.89	1.73E-03	0.014
Rab4b	0.95	1.73E-03	0.014
Mst1r	1.27	1.72E-03	0.014
Gdf11	1.15	1.73E-03	0.014
Uqcrfs1	0.91	1.73E-03	0.014
Atp6v0e2	0.86	1.73E-03	0.014
Pin1	0.83	1.74E-03	0.014
Tmem167b	0.93	1.75E-03	0.014
Jmjd1c	1.12	1.75E-03	0.014
Gm960	1.31	1.76E-03	0.014
Zfat	0.79	1.76E-03	0.014
Ryr3	1.02	1.76E-03	0.014
Eif1	0.97	1.77E-03	0.014
Trank1	1.10	1.77E-03	0.014

Yipf6	1.05	1.77E-03	0.014
Jarid2	1.06	1.77E-03	0.014
Dennd4a	1.02	1.78E-03	0.014
Tubb2b	0.96	1.78E-03	0.014
Znhit2	0.87	1.79E-03	0.014
Ndufc1	0.88	1.79E-03	0.014
Arl6ip1	0.92	1.79E-03	0.014
Sorl1	1.09	1.79E-03	0.014
BC037034	0.91	1.79E-03	0.014
Podn	1.30	1.80E-03	0.014
Akap9	1.10	1.80E-03	0.014
Setd2	1.06	1.80E-03	0.014
Hic2	1.05	1.81E-03	0.014
Gm12060	0.77	1.81E-03	0.014
Alg10b	1.07	1.81E-03	0.014
Ak1	0.90	1.81E-03	0.014
Etfb	0.89	1.81E-03	0.014
Rtbdn	1.24	1.81E-03	0.014
Pxdn	1.06	1.82E-03	0.014
Stx16	1.08	1.82E-03	0.014
Ngdn	0.91	1.82E-03	0.014
Plxna1	1.05	1.82E-03	0.014
Apc2	1.03	1.83E-03	0.014
Rnf169	1.06	1.83E-03	0.014
Mmp16	1.11	1.84E-03	0.014
Slc35f1	1.07	1.84E-03	0.014
Acot7	0.84	1.84E-03	0.014
Tnks	1.05	1.84E-03	0.014
Srsf5	1.20	1.85E-03	0.014
Cnr1	1.11	1.85E-03	0.014
Txnl4a	0.86	1.86E-03	0.014
Eif2b1	0.92	1.86E-03	0.014
Stag3	1.20	1.86E-03	0.014
Arl6ip4	0.93	1.86E-03	0.014
Acp1	0.89	1.86E-03	0.014
Nrip1	1.18	1.86E-03	0.014
Hcfc1r1	0.93	1.86E-03	0.014
Ndufb2	0.86	1.86E-03	0.014
Maged1	0.96	1.87E-03	0.014
Plekhg1	1.09	1.88E-03	0.014
Nav3	1.02	1.88E-03	0.014
Cep250	1.06	1.88E-03	0.014
Fcgrt	1.29	1.88E-03	0.014
Igsf6	1.44	1.88E-03	0.014
Ptprs	1.05	1.88E-03	0.014
Uchl4	0.94	1.88E-03	0.014
Stxbp5l	1.07	1.89E-03	0.014
Slc22a8	1.15	1.89E-03	0.014
Rpl13	0.94	1.89E-03	0.014

Zbtb16	1.08	1.89E-03	0.014
Psmd4	0.99	1.89E-03	0.014
Usp49	1.10	1.91E-03	0.015
Ano6	1.04	1.91E-03	0.015
Ubr5	1.05	1.92E-03	0.015
Pml	1.07	1.92E-03	0.015
Lancl3	1.16	1.93E-03	0.015
Slc7a8	1.12	1.93E-03	0.015
Rps18	0.92	1.93E-03	0.015
Mrps22	0.93	1.93E-03	0.015
Psmb5	0.90	1.94E-03	0.015
Chrac1	0.87	1.94E-03	0.015
Spon2	1.42	1.94E-03	0.015
Mrpl41	0.86	1.95E-03	0.015
Aspscr1	0.92	1.96E-03	0.015
Hsbp1	0.93	1.98E-03	0.015
Gtf2h5	0.99	1.99E-03	0.015
1700096K18Rik	0.85	1.99E-03	0.015
Kcnb1	1.03	2.00E-03	0.015
C77370	1.07	2.01E-03	0.015
Pold2	0.87	2.03E-03	0.015
Ptar1	1.16	2.03E-03	0.015
Ikzf4	1.13	2.04E-03	0.015
Cldn1	1.24	2.04E-03	0.015
Slco2b1	1.07	2.03E-03	0.015
Ppp2r3a	1.03	2.04E-03	0.015
Gria3	1.09	2.04E-03	0.015
Vps52	0.92	2.05E-03	0.015
Ubac1	0.86	2.05E-03	0.015
Mob1b	1.15	2.05E-03	0.015
Trip12	1.03	2.06E-03	0.015
Grik3	1.05	2.06E-03	0.015
Slc52a2	0.86	2.07E-03	0.016
Sema4g	1.05	2.07E-03	0.016
Med10	0.98	2.08E-03	0.016
Atp6v1b2	0.87	2.08E-03	0.016
Sh3bgrl3	0.93	2.09E-03	0.016
Kptn	0.89	2.09E-03	0.016
Klhdc4	0.89	2.09E-03	0.016
Slirp	0.89	2.10E-03	0.016
Csnk2b	0.96	2.11E-03	0.016
Gpr156	1.08	2.11E-03	0.016
Cox7b	0.93	2.11E-03	0.016
Qsox1	1.12	2.12E-03	0.016
Vps13b	0.99	2.12E-03	0.016
Ebna1bp2	0.89	2.12E-03	0.016
Sec61g	0.82	2.12E-03	0.016
Chmp7	0.89	2.12E-03	0.016
Rab3b	0.83	2.12E-03	0.016

Mob3a	0.93	2.13E-03	0.016
Mgat5	1.10	2.13E-03	0.016
Sigmar1	0.89	2.13E-03	0.016
Col25a1	1.15	2.14E-03	0.016
Fam207a	0.90	2.14E-03	0.016
Trim46	0.91	2.14E-03	0.016
Ldlrad4	1.15	2.14E-03	0.016
Yap1	1.11	2.15E-03	0.016
Mrps21	0.93	2.15E-03	0.016
Dock7	1.11	2.15E-03	0.016
Rpl35	0.95	2.16E-03	0.016
Fam228b	1.26	2.16E-03	0.016
8030462N17Rik	1.11	2.17E-03	0.016
Actr3b	0.89	2.17E-03	0.016
Ddx1	0.92	2.18E-03	0.016
Map3k13	1.04	2.18E-03	0.016
Ergic3	0.97	2.18E-03	0.016
2900076A07Rik	1.32	2.18E-03	0.016
Ralgapa2	1.05	2.19E-03	0.016
Necap1	0.90	2.18E-03	0.016
Psmd13	0.94	2.19E-03	0.016
Polr2h	0.91	2.19E-03	0.016
Bmyc	0.95	2.19E-03	0.016
Fmn2	1.02	2.20E-03	0.016
Camsap1	0.98	2.20E-03	0.016
Rp9	0.96	2.21E-03	0.016
Gamt	0.83	2.22E-03	0.016
Sox8	0.88	2.22E-03	0.016
Rpl7	0.97	2.22E-03	0.016
Bcorl1	1.07	2.23E-03	0.016
Nucks1	1.03	2.23E-03	0.016
Arid5b	1.12	2.24E-03	0.016
Cast	1.34	2.24E-03	0.016
Ndufa2	0.89	2.24E-03	0.016
Scfd1	0.91	2.25E-03	0.016
Atp6v0d1	0.93	2.25E-03	0.016
Ralgps2	1.01	2.26E-03	0.016
Dcdc2a	1.21	2.26E-03	0.016
Dgcr6	0.92	2.26E-03	0.016
Chmp4b	0.94	2.26E-03	0.016
Foxj3	1.05	2.26E-03	0.016
Pdzd11	0.95	2.26E-03	0.016
Actr10	0.91	2.27E-03	0.016
Jagn1	0.92	2.27E-03	0.016
Med28	0.80	2.27E-03	0.016
Larp4	1.09	2.27E-03	0.016
Ptpn4	1.14	2.28E-03	0.017
Smarcb1	0.95	2.29E-03	0.017
Crebbp	1.08	2.29E-03	0.017

Clstn2	1.13	2.29E-03	0.017
Gria2	1.14	2.30E-03	0.017
Kcnc1	1.03	2.31E-03	0.017
Inha	1.21	2.31E-03	0.017
Prkcq	0.78	2.31E-03	0.017
Tpi1	0.84	2.31E-03	0.017
Pgam1	0.90	2.33E-03	0.017
Syt5	0.94	2.33E-03	0.017
Chrna7	1.13	2.33E-03	0.017
Phyhd1	1.25	2.33E-03	0.017
Atp8a2	1.08	2.34E-03	0.017
Tac1	0.92	2.34E-03	0.017
Lamtor1	0.94	2.35E-03	0.017
Mrpl22	0.90	2.35E-03	0.017
Cox5b	0.92	2.35E-03	0.017
Polr2i	0.94	2.35E-03	0.017
Mtx3	1.06	2.36E-03	0.017
Psmc1	0.95	2.36E-03	0.017
Klhdc10	1.04	2.37E-03	0.017
C1qtnf1	1.12	2.37E-03	0.017
Tmem150a	1.22	2.39E-03	0.017
Imp3	0.94	2.39E-03	0.017
Arhgef1	1.18	2.40E-03	0.017
Fkbp4	0.90	2.40E-03	0.017
Cdc16	0.93	2.40E-03	0.017
Fkbp1b	0.90	2.41E-03	0.017
Smim12	0.91	2.41E-03	0.017
Pwwp2a	1.05	2.42E-03	0.017
Lsm2	0.92	2.42E-03	0.017
Eif4g3	1.06	2.43E-03	0.017
Dscr3	0.89	2.43E-03	0.017
Fabp7	0.96	2.44E-03	0.017
Gosr2	0.91	2.44E-03	0.017
Usp45	1.11	2.44E-03	0.017
Ptpru	1.06	2.44E-03	0.017
Polr2d	0.95	2.45E-03	0.017
Gng10	0.97	2.45E-03	0.017
C530008M17Rik	1.03	2.48E-03	0.018
Syt7	1.03	2.48E-03	0.018
Sstr4	1.28	2.49E-03	0.018
Trim33	1.03	2.49E-03	0.018
Zfhx3	1.09	2.50E-03	0.018
Inpp5e	1.05	2.51E-03	0.018
Kit	1.05	2.51E-03	0.018
Abcc5	1.11	2.51E-03	0.018
Spopl	1.13	2.53E-03	0.018
Ankrd24	1.16	2.54E-03	0.018
Birc6	1.02	2.55E-03	0.018
Pccb	0.88	2.55E-03	0.018

Nynrin	1.03	2.56E-03	0.018
Isca1	0.93	2.57E-03	0.018
Tmem256	0.90	2.57E-03	0.018
Pabpc4l	1.18	2.58E-03	0.018
Tmem64	1.10	2.59E-03	0.018
Acyp1	0.91	2.59E-03	0.018
Ctnnd1	1.08	2.59E-03	0.018
Snrnp40	0.90	2.59E-03	0.018
Gramd1b	1.10	2.59E-03	0.018
Pfdn2	0.98	2.60E-03	0.018
Erh	0.97	2.61E-03	0.018
Cenpm	0.80	2.61E-03	0.018
Pik3c2b	1.07	2.62E-03	0.018
Dkk2	1.23	2.62E-03	0.018
Osbpl8	1.08	2.62E-03	0.018
Slc23a2	1.03	2.62E-03	0.018
Ccdc91	0.88	2.63E-03	0.018
Mtcp1	1.15	2.63E-03	0.018
B3galnt2	1.18	2.63E-03	0.018
Xkr6	1.15	2.65E-03	0.018
Ubxn1	0.97	2.65E-03	0.019
Hspd1	0.95	2.66E-03	0.019
Slc1a2	1.04	2.66E-03	0.019
Fxn	0.87	2.67E-03	0.019
Chd1	1.09	2.68E-03	0.019
9330159M07Rik	1.33	2.69E-03	0.019
Taf9	0.92	2.70E-03	0.019
Dynlrb1	0.96	2.70E-03	0.019
Hsd17b7	0.83	2.71E-03	0.019
Sec63	1.07	2.71E-03	0.019
Npc1	1.07	2.71E-03	0.019
Ubr4	0.98	2.71E-03	0.019
Apitd1	0.83	2.72E-03	0.019
Dock5	1.14	2.72E-03	0.019
Megf10	1.03	2.73E-03	0.019
Mtfr1l	0.93	2.73E-03	0.019
Lrrc32	1.15	2.73E-03	0.019
Aff2	1.12	2.73E-03	0.019
Slc35d1	1.13	2.73E-03	0.019
Psmg1	0.92	2.73E-03	0.019
Sdhaf1	0.95	2.73E-03	0.019
Hspa12a	1.03	2.74E-03	0.019
Mars2	1.14	2.75E-03	0.019
Ap5m1	1.13	2.75E-03	0.019
Irak1bp1	0.92	2.75E-03	0.019
Frs2	1.05	2.76E-03	0.019
Asap2	1.06	2.76E-03	0.019
Psenen	0.93	2.76E-03	0.019
Npdc1	0.96	2.76E-03	0.019

Sos1	1.04	2.77E-03	0.019
Setbp1	1.08	2.77E-03	0.019
Rictor	1.07	2.77E-03	0.019
Pofut2	0.92	2.78E-03	0.019
Ror2	1.25	2.78E-03	0.019
Ung	0.79	2.78E-03	0.019
Scn3a	1.05	2.79E-03	0.019
Exoc7	0.92	2.80E-03	0.019
Csf1	1.14	2.80E-03	0.019
Aimp1	0.95	2.81E-03	0.019
Tet2	1.08	2.82E-03	0.019
Cers4	1.15	2.82E-03	0.019
Prcc	0.93	2.83E-03	0.019
Rock1	1.16	2.83E-03	0.019
Ubl7	0.94	2.83E-03	0.019
Slc16a9	1.26	2.84E-03	0.019
Arglu1	1.23	2.84E-03	0.019
Nt5c	0.97	2.84E-03	0.019
Fmn1	1.13	2.85E-03	0.019
Ppp1r9a	1.02	2.85E-03	0.019
Cfb	1.15	2.87E-03	0.019
Ece1	1.07	2.87E-03	0.019
Psmd12	0.95	2.87E-03	0.020
Zfp516	1.10	2.88E-03	0.020
Il17rd	1.05	2.88E-03	0.020
Gm7102	0.79	2.88E-03	0.020
Ptdss1	0.89	2.89E-03	0.020
Edaradd	1.01	2.90E-03	0.020
Klf2	1.21	2.91E-03	0.020
Phax	0.92	2.92E-03	0.020
Slc29a3	1.10	2.92E-03	0.020
Tgfbi	1.29	2.93E-03	0.020
Slc25a3	0.91	2.93E-03	0.020
Zfp652	1.16	2.95E-03	0.020
2810013P06Rik	0.90	2.95E-03	0.020
Tmod2	1.01	2.96E-03	0.020
Crispld1	1.25	2.96E-03	0.020
Mrpl2	0.94	2.96E-03	0.020
Notch1	1.03	2.96E-03	0.020
Cops2	0.94	2.96E-03	0.020
Ncbp2	0.95	2.97E-03	0.020
Dnmt3b	1.16	2.97E-03	0.020
Ppp4r1l-ps	1.09	2.98E-03	0.020
Steap3	1.22	2.98E-03	0.020
Dlg1	1.06	2.98E-03	0.020
Jmjd8	0.92	2.99E-03	0.020
Sec16a	1.02	2.99E-03	0.020
Fam213b	0.85	2.99E-03	0.020
Ola1	0.92	2.99E-03	0.020

Glis3	1.21	3.00E-03	0.020
Usp34	1.01	3.01E-03	0.020
Guk1	0.88	3.01E-03	0.020
Snap47	0.90	3.02E-03	0.020
Ppm1l	1.08	3.03E-03	0.020
Eif4ebp1	0.67	3.03E-03	0.020
Fam160b1	1.06	3.03E-03	0.020
Rfc2	0.93	3.03E-03	0.020
Rps9	0.90	3.04E-03	0.020
Sdk2	1.07	3.05E-03	0.020
Ppp2cb	0.96	3.05E-03	0.020
Dlst	0.94	3.06E-03	0.020
Cbx5	0.99	3.06E-03	0.020
Rab3a	0.92	3.06E-03	0.020
Ctu2	0.83	3.06E-03	0.020
Rps27a	0.91	3.07E-03	0.020
Ylpm1	1.09	3.07E-03	0.020
Agtrap	1.15	3.07E-03	0.020
Scarf2	1.26	3.08E-03	0.020
Wfikkn2	1.21	3.10E-03	0.021
Unc13a	0.99	3.10E-03	0.021
Kif5c	1.04	3.10E-03	0.021
Stxbp2	1.20	3.11E-03	0.021
Kcp	1.19	3.11E-03	0.021
Rufy2	1.06	3.12E-03	0.021
Prkaa2	1.03	3.14E-03	0.021
Rgs7bp	1.05	3.15E-03	0.021
Slc2a1	1.15	3.15E-03	0.021
Ercc8	0.88	3.15E-03	0.021
Gab1	1.08	3.15E-03	0.021
Wnt2b	1.15	3.15E-03	0.021
R3hdm1	1.08	3.16E-03	0.021
Rpl30	0.86	3.16E-03	0.021
Fabp5	0.98	3.17E-03	0.021
Pfn1	0.99	3.17E-03	0.021
E230016M11Rik	1.41	3.18E-03	0.021
Mccc2	0.89	3.18E-03	0.021
Camk1d	1.01	3.19E-03	0.021
Ercc1	0.90	3.20E-03	0.021
Zfp382	1.13	3.20E-03	0.021
Yipf5	0.93	3.20E-03	0.021
Dyrk1a	1.01	3.22E-03	0.021
Unc5c	1.06	3.23E-03	0.021
Dctn6	0.99	3.24E-03	0.021
Gapvd1	1.02	3.25E-03	0.021
Fam96b	0.98	3.25E-03	0.021
Umps	0.88	3.26E-03	0.021
Huwe1	1.00	3.26E-03	0.021
Oxct1	0.85	3.26E-03	0.021

Dclk2	1.08	3.27E-03	0.021
Mir682	0.93	3.27E-03	0.021
Zfp597	1.02	3.28E-03	0.021
Dgke	1.06	3.28E-03	0.021
Ep300	1.07	3.28E-03	0.021
Vps29	0.98	3.28E-03	0.021
Nfatc4	1.16	3.29E-03	0.021
Pear1	1.22	3.30E-03	0.021
Pigf	0.89	3.31E-03	0.022
Fam160a2	0.97	3.31E-03	0.022
Dmxl2	1.03	3.32E-03	0.022
Noc4l	0.86	3.33E-03	0.022
Atad2b	1.07	3.34E-03	0.022
Ankrd54	0.88	3.34E-03	0.022
Nif3l1	0.93	3.36E-03	0.022
Pmm1	0.93	3.36E-03	0.022
Ormdl1	0.92	3.37E-03	0.022
Adi1	0.88	3.37E-03	0.022
Klhl20	1.10	3.39E-03	0.022
AI837181	0.94	3.39E-03	0.022
Vps37a	1.04	3.39E-03	0.022
Mpst	0.92	3.40E-03	0.022
Mrpl13	0.95	3.42E-03	0.022
Yars2	0.88	3.42E-03	0.022
Hadha	0.91	3.42E-03	0.022
Hist3h2ba	0.92	3.43E-03	0.022
Gpatch8	1.07	3.43E-03	0.022
Stc2	0.75	3.43E-03	0.022
Zfp358	0.94	3.43E-03	0.022
Cpsf6	1.10	3.44E-03	0.022
Rcan1	0.88	3.46E-03	0.022
Slc12a6	1.02	3.46E-03	0.022
Tanc1	1.06	3.46E-03	0.022
Mt3	0.91	3.47E-03	0.022
A330023F24Rik	1.10	3.48E-03	0.022
Sfi1	1.15	3.48E-03	0.022
Tmem67	1.12	3.47E-03	0.022
Bmp5	1.33	3.47E-03	0.022
Map3k1	1.01	3.49E-03	0.022
Rpl38	0.88	3.49E-03	0.022
Ankrd61	1.53	3.49E-03	0.022
Pkd2	1.05	3.50E-03	0.022
Nat14	0.89	3.51E-03	0.022
Wwtr1	1.08	3.52E-03	0.022
Prr12	1.02	3.52E-03	0.023
Samm50	0.94	3.53E-03	0.023
Kcnj6	1.17	3.53E-03	0.023
Pef1	0.90	3.53E-03	0.023
Dpy19l4	1.14	3.53E-03	0.023

Med12l	1.03	3.54E-03	0.023
Creld2	0.90	3.54E-03	0.023
Pigs	0.89	3.55E-03	0.023
Zfyve19	0.90	3.55E-03	0.023
Dnajb11	0.90	3.56E-03	0.023
Coro1a	0.90	3.56E-03	0.023
Bex2	0.96	3.56E-03	0.023
Gtf3a	0.94	3.57E-03	0.023
Arl1	0.95	3.59E-03	0.023
Ptcd2	0.93	3.59E-03	0.023
Ddx17	1.08	3.59E-03	0.023
Nfia	1.13	3.59E-03	0.023
Mapk1ip1l	1.06	3.60E-03	0.023
Zyg11b	0.98	3.60E-03	0.023
Rabac1	0.89	3.61E-03	0.023
Scp2	0.91	3.61E-03	0.023
Map1b	1.04	3.61E-03	0.023
Kcnq2	1.01	3.63E-03	0.023
Pdap1	0.92	3.63E-03	0.023
Mpp7	1.07	3.64E-03	0.023
Rhof	0.81	3.64E-03	0.023
Abcb1a	1.07	3.66E-03	0.023
Ciita	1.30	3.67E-03	0.023
Akap8l	1.13	3.67E-03	0.023
Vegfa	1.10	3.68E-03	0.023
Zfp532	1.06	3.67E-03	0.023
Ncoa2	1.00	3.68E-03	0.023
Eya3	1.06	3.69E-03	0.023
Surf1	0.93	3.71E-03	0.023
Wdr86	1.28	3.71E-03	0.023
Il17ra	1.12	3.71E-03	0.023
2900011O08Rik	0.88	3.71E-03	0.023
Secisbp2l	1.00	3.72E-03	0.023
Rfx7	1.05	3.72E-03	0.023
Tgs1	1.09	3.73E-03	0.023
Cuedc2	0.91	3.73E-03	0.023
Bcl10	0.92	3.73E-03	0.023
Col2a1	1.18	3.75E-03	0.023
Fzd1	1.13	3.78E-03	0.024
Dcn	1.40	3.78E-03	0.024
Aplnr	1.30	3.79E-03	0.024
Dgkh	1.16	3.79E-03	0.024
Gde1	0.91	3.79E-03	0.024
Mylk	1.11	3.81E-03	0.024
Stoml2	0.93	3.81E-03	0.024
Loxl1	1.21	3.83E-03	0.024
Plekha6	1.08	3.84E-03	0.024
Olig1	0.79	3.84E-03	0.024
Dclre1c	1.10	3.85E-03	0.024

Noa1	0.91	3.86E-03	0.024
Gm14164	1.40	3.86E-03	0.024
Cmss1	0.88	3.86E-03	0.024
Strn	1.07	3.87E-03	0.024
Notch3	1.05	3.90E-03	0.024
Psma1	0.97	3.91E-03	0.024
Sp1	1.06	3.92E-03	0.024
Rrbp1	1.08	3.92E-03	0.024
Atxn1l	1.01	3.92E-03	0.024
Zcchc17	0.94	3.92E-03	0.024
Cox20	1.32	3.93E-03	0.024
Vldlr	1.02	3.93E-03	0.024
St8sia2	1.08	3.93E-03	0.024
Plekhg3	1.18	3.95E-03	0.024
Eml6	1.08	3.96E-03	0.024
Tmem70	0.93	3.97E-03	0.025
Ndufs6	0.92	3.97E-03	0.025
Adamts6	1.15	3.99E-03	0.025
Slc35c2	0.88	3.98E-03	0.025
Ermap	1.31	3.98E-03	0.025
Slc6a6	1.03	3.98E-03	0.025
Cyth2	0.91	3.98E-03	0.025
Klf13	1.05	3.99E-03	0.025
Smpd1	0.88	3.99E-03	0.025
Rpl14	0.95	3.98E-03	0.025
Sqstm1	0.90	3.99E-03	0.025
Plekha5	1.06	4.01E-03	0.025
Atxn7	1.05	4.02E-03	0.025
Pdcd6ip	1.03	4.02E-03	0.025
Tab3	1.03	4.02E-03	0.025
Usp39	0.91	4.03E-03	0.025
Msrb3	1.16	4.04E-03	0.025
Spcs1	0.95	4.05E-03	0.025
Napa	0.93	4.05E-03	0.025
Hdhd2	0.87	4.07E-03	0.025
Fkbp14	1.12	4.07E-03	0.025
Uspl1	0.90	4.07E-03	0.025
Snx18	1.04	4.08E-03	0.025
Yif1a	0.87	4.09E-03	0.025
Srrm4	1.09	4.09E-03	0.025
Klf3	1.14	4.11E-03	0.025
Chd4	1.06	4.12E-03	0.025
Bnip1	0.93	4.13E-03	0.025
Pclo	1.02	4.14E-03	0.025
Mrpl14	0.95	4.14E-03	0.025
Enah	1.06	4.16E-03	0.025
Slc30a10	1.04	4.15E-03	0.025
Rapgef6	1.09	4.16E-03	0.025
Amer2	1.12	4.15E-03	0.025

Serpind1	1.45	4.16E-03	0.025
Lrch3	1.05	4.15E-03	0.025
Acat3	0.84	4.16E-03	0.025
Mapkap1	0.93	4.16E-03	0.025
Mbnl1	1.04	4.16E-03	0.025
Cldn11	1.59	4.18E-03	0.025
Cpne1	1.09	4.18E-03	0.025
Snrpe	0.92	4.18E-03	0.025
Thoc3	0.90	4.18E-03	0.025
Dkk3	1.12	4.19E-03	0.025
Ndufv3	0.91	4.19E-03	0.025
1700028B04Rik	0.76	4.20E-03	0.025
Kcnt2	1.13	4.22E-03	0.026
Phf14	1.03	4.22E-03	0.026
Clock	1.05	4.23E-03	0.026
Cck	0.84	4.23E-03	0.026
Egr1	1.13	4.25E-03	0.026
Txnl1	0.92	4.26E-03	0.026
Cox8a	0.93	4.26E-03	0.026
Polr2g	0.94	4.26E-03	0.026
Phospho2	0.93	4.31E-03	0.026
Rpusd1	0.92	4.31E-03	0.026
Emc6	0.94	4.32E-03	0.026
Gmds	0.90	4.32E-03	0.026
Csf1r	1.11	4.33E-03	0.026
Cnbp	0.92	4.33E-03	0.026
Brwd3	1.10	4.33E-03	0.026
Msto1	0.88	4.34E-03	0.026
Fam174a	0.85	4.36E-03	0.026
Rnf170	1.10	4.36E-03	0.026
Zc3hav1	1.34	4.37E-03	0.026
Slc2a10	1.35	4.38E-03	0.026
Ep400	1.03	4.38E-03	0.026
Dctn2	0.94	4.39E-03	0.026
Tpcn1	1.07	4.39E-03	0.026
Ap2a1	0.91	4.39E-03	0.026
Psme2	0.89	4.41E-03	0.026
Fbln1	1.14	4.42E-03	0.026
Gli3	1.08	4.43E-03	0.026
Jkamp	0.94	4.43E-03	0.026
Twsg1	1.09	4.44E-03	0.027
Nubp1	0.84	4.44E-03	0.027
Raph1	1.02	4.45E-03	0.027
Lonrf1	1.07	4.47E-03	0.027
Prpf19	0.87	4.48E-03	0.027
Atp2a2	1.05	4.48E-03	0.027
Dnlz	0.88	4.48E-03	0.027
Foxj2	1.05	4.49E-03	0.027
1700123O20Rik	0.92	4.50E-03	0.027

Rab11fip3	1.03	4.50E-03	0.027
Efna3	0.91	4.50E-03	0.027
Rab23	1.04	4.51E-03	0.027
Trappc1	0.97	4.51E-03	0.027
Clptm1l	0.91	4.51E-03	0.027
Gpr19	0.87	4.51E-03	0.027
Zfp511	0.95	4.51E-03	0.027
Cdc42bpb	0.99	4.53E-03	0.027
Znfx1	1.00	4.53E-03	0.027
Dennd3	1.12	4.54E-03	0.027
Idh3a	0.90	4.54E-03	0.027
Sumo1	0.98	4.55E-03	0.027
Qpctl	0.88	4.55E-03	0.027
Mocs2	0.93	4.57E-03	0.027
Slc6a17	1.01	4.58E-03	0.027
Urm1	0.87	4.58E-03	0.027
Ddx19b	0.99	4.59E-03	0.027
Coro7	1.11	4.60E-03	0.027
Eral1	0.88	4.61E-03	0.027
Rpl27	0.95	4.62E-03	0.027
Gstp2	0.92	4.64E-03	0.027
Pgam5	0.91	4.65E-03	0.027
Rpl22l1	0.87	4.65E-03	0.027
Fahd1	0.87	4.68E-03	0.027
Pdrg1	0.94	4.68E-03	0.027
Usp5	0.92	4.68E-03	0.027
Catsperg1	1.35	4.68E-03	0.028
Abca2	1.00	4.69E-03	0.028
Jag1	1.09	4.69E-03	0.028
Abca8a	1.24	4.70E-03	0.028
Zc3h15	0.95	4.70E-03	0.028
Itgb3	1.13	4.71E-03	0.028
Fbxo48	1.34	4.72E-03	0.028
Klhl28	1.14	4.72E-03	0.028
Dbndd1	0.90	4.72E-03	0.028
Utrn	0.98	4.73E-03	0.028
Ptpra	0.93	4.74E-03	0.028
Sema6a	1.10	4.74E-03	0.028
Dnm3	1.09	4.76E-03	0.028
Adh5	0.94	4.76E-03	0.028
Med13l	1.05	4.76E-03	0.028
Rpl10a	0.96	4.76E-03	0.028
Trpm7	1.08	4.77E-03	0.028
Kank3	1.10	4.78E-03	0.028
Sirt2	0.96	4.79E-03	0.028
Pign	1.01	4.80E-03	0.028
Impdh1	0.90	4.80E-03	0.028
Coq2	0.93	4.81E-03	0.028
Rlim	1.02	4.82E-03	0.028

Rcsd1	1.17	4.84E-03	0.028
Tenm4	1.06	4.85E-03	0.028
Celf1	1.05	4.86E-03	0.028
Lias	0.93	4.88E-03	0.028
Gm1673	0.94	4.89E-03	0.028
Cmklr1	1.19	4.90E-03	0.028
2310061I04Rik	0.94	4.90E-03	0.028
L1cam	1.08	4.90E-03	0.028
Capn10	0.85	4.91E-03	0.028
Ranbp3	0.90	4.93E-03	0.029
Rps6	0.95	4.93E-03	0.029
1110065P20Rik	0.88	4.94E-03	0.029
Sccpdh	0.91	4.97E-03	0.029
Psmd2	0.94	4.97E-03	0.029
Cpne3	1.06	4.97E-03	0.029
Zkscan1	1.02	4.98E-03	0.029
Nsg1	0.93	5.00E-03	0.029
Axin2	1.14	5.02E-03	0.029
Ankrd16	1.17	5.02E-03	0.029
Ireb2	1.06	5.03E-03	0.029
Gria1	1.04	5.03E-03	0.029
Mrps33	0.92	5.03E-03	0.029
Ogfod3	0.87	5.05E-03	0.029
Triqk	0.90	5.06E-03	0.029
Fnta	0.92	5.07E-03	0.029
Aars2	0.85	5.09E-03	0.029
Irf2bp2	1.09	5.09E-03	0.029
Klhl15	1.09	5.09E-03	0.029
Tas1r1	1.12	5.10E-03	0.029
Strn4	0.92	5.10E-03	0.029
Mob3b	1.17	5.11E-03	0.029
Brk1	0.97	5.11E-03	0.029
Dip2a	1.01	5.13E-03	0.029
Sf3b5	0.93	5.15E-03	0.029
Gtf3c5	0.91	5.14E-03	0.029
Mov10	1.14	5.14E-03	0.029
Uchl1	0.94	5.14E-03	0.029
Herc2	0.97	5.15E-03	0.029
Ppan	0.92	5.15E-03	0.029
Stx8	0.84	5.19E-03	0.030
Mnat1	0.87	5.20E-03	0.030
Rps6ka3	1.11	5.20E-03	0.030
Usp37	1.03	5.21E-03	0.030
Prkg1	1.05	5.22E-03	0.030
Slit2	1.04	5.23E-03	0.030
Ppp2ca	0.95	5.23E-03	0.030
Csmd1	1.00	5.24E-03	0.030
B3galt6	0.89	5.26E-03	0.030
Rtn2	0.92	5.26E-03	0.030

Pggt1b	1.14	5.26E-03	0.030
B4galt1	1.13	5.27E-03	0.030
Nrxn1	1.07	5.28E-03	0.030
Myo1c	1.05	5.28E-03	0.030
Grn	1.12	5.28E-03	0.030
Rgcc	0.85	5.31E-03	0.030
Elp4	0.88	5.31E-03	0.030
Myo9b	1.09	5.33E-03	0.030
Bcas2	0.99	5.35E-03	0.030
Zfp266	1.07	5.36E-03	0.030
Map2	1.06	5.36E-03	0.030
Calb1	0.86	5.36E-03	0.030
Ccdc116	0.81	5.39E-03	0.031
Zfp536	1.09	5.39E-03	0.031
Gcdh	0.89	5.39E-03	0.031
Bdp1	1.04	5.40E-03	0.031
Adamts20	1.07	5.40E-03	0.031
Srrm2	1.10	5.40E-03	0.031
Ctns	1.09	5.43E-03	0.031
Mmrn2	1.17	5.43E-03	0.031
Sfxn2	1.13	5.46E-03	0.031
Mrps35	0.95	5.47E-03	0.031
Kdsr	1.02	5.49E-03	0.031
Naca	0.82	5.50E-03	0.031
Mpv17	0.94	5.51E-03	0.031
Nhsl2	1.09	5.51E-03	0.031
Mycbp	1.20	5.52E-03	0.031
Astn1	1.05	5.53E-03	0.031
Trap1	0.92	5.53E-03	0.031
Zfp518b	1.06	5.56E-03	0.031
Eif4a1	0.90	5.57E-03	0.031
Larp7	0.91	5.58E-03	0.031
Cep170	1.08	5.59E-03	0.031
Sars2	0.84	5.59E-03	0.031
Arhgef9	1.08	5.59E-03	0.031
Rnaseh2c	0.89	5.61E-03	0.031
Dixdc1	1.06	5.62E-03	0.032
Slc16a6	1.13	5.63E-03	0.032
Abhd4	0.90	5.63E-03	0.032
B230118H07Rik	0.88	5.63E-03	0.032
Ube2e1	0.95	5.64E-03	0.032
Tmem33	1.01	5.64E-03	0.032
Nhsl1	1.05	5.65E-03	0.032
Adamts3	1.12	5.65E-03	0.032
Snrpa1	0.93	5.65E-03	0.032
Ppig	1.05	5.66E-03	0.032
Tnks1bp1	1.05	5.66E-03	0.032
Rabggtb	0.92	5.67E-03	0.032
Timm8a1	0.92	5.68E-03	0.032

Tbc1d23	1.10	5.69E-03	0.032
Dag1	1.05	5.69E-03	0.032
Abca8b	1.16	5.70E-03	0.032
Mbd5	1.03	5.71E-03	0.032
Ahdc1	1.03	5.71E-03	0.032
Nat9	0.87	5.72E-03	0.032
Psmd3	0.95	5.73E-03	0.032
Ctla2a	0.74	5.75E-03	0.032
Pebp1	0.91	5.75E-03	0.032
Pfkl	0.86	5.75E-03	0.032
St8sia5	0.78	5.77E-03	0.032
Cxx1a	0.94	5.77E-03	0.032
Malat1	1.28	5.77E-03	0.032
Gdpd3	1.47	5.79E-03	0.032
Ddx6	1.03	5.80E-03	0.032
Pfkp	1.21	5.80E-03	0.032
Dohh	0.91	5.81E-03	0.032
Znhit6	0.89	5.81E-03	0.032
Nosip	0.92	5.81E-03	0.032
Mrps10	0.92	5.82E-03	0.032
Uqcr11	0.89	5.83E-03	0.032
Ccdc88a	1.07	5.83E-03	0.032
Setd3	0.93	5.83E-03	0.032
Rasl10b	0.87	5.83E-03	0.032
F8a	0.87	5.84E-03	0.032
Pgk1	0.87	5.86E-03	0.032
Atf6b	0.90	5.87E-03	0.032
Numb	1.08	5.88E-03	0.032
Cxx1b	0.94	5.89E-03	0.032
Nek9	1.04	5.89E-03	0.032
Foxo3	1.12	5.91E-03	0.033
Nob1	0.92	5.91E-03	0.033
Cnih2	0.94	5.93E-03	0.033
Tjp2	1.05	5.93E-03	0.033
Rpp30	0.92	5.93E-03	0.033
Pik3ca	1.00	5.93E-03	0.033
Mtf1	0.98	5.93E-03	0.033
Heatr3	0.90	5.92E-03	0.033
Smad6	1.23	5.95E-03	0.033
Zfp462	1.09	5.96E-03	0.033
Plekhm3	1.00	5.98E-03	0.033
Rcbtb1	1.13	5.98E-03	0.033
Zfand2b	0.90	6.01E-03	0.033
Cnpy3	0.91	6.01E-03	0.033
Ctif	1.02	6.01E-03	0.033
Rhov	0.88	6.01E-03	0.033
Tmem234	0.99	6.02E-03	0.033
Zmynd8	1.04	6.02E-03	0.033
Cpox	1.10	6.04E-03	0.033

Rac3	0.89	6.04E-03	0.033
Lmtk3	1.05	6.06E-03	0.033
1500011K16Rik	0.85	6.06E-03	0.033
Itfg2	0.91	6.08E-03	0.033
Psmb4	0.91	6.09E-03	0.033
Slc35e2	1.00	6.10E-03	0.033
Adcy9	1.06	6.11E-03	0.033
Itga4	1.01	6.11E-03	0.033
Ddx49	0.89	6.12E-03	0.033
H2-M5	1.39	6.14E-03	0.033
Sycp3	1.41	6.15E-03	0.033
Smok4a	1.51	6.15E-03	0.033
Swi5	0.98	6.15E-03	0.033
Creb5	1.18	6.14E-03	0.033
Dlg5	0.99	6.16E-03	0.033
Rtca	0.93	6.16E-03	0.033
Mrpl53	0.94	6.16E-03	0.033
Slc12a2	1.08	6.16E-03	0.033
Rpl23a	0.99	6.19E-03	0.034
Cln8	0.86	6.19E-03	0.034
Pcdhgb7	1.08	6.22E-03	0.034
Acaa2	0.93	6.22E-03	0.034
Ammecr1	1.12	6.23E-03	0.034
Akr7a5	0.86	6.23E-03	0.034
Rab28	0.93	6.24E-03	0.034
Eef2k	1.00	6.26E-03	0.034
Stard8	1.06	6.26E-03	0.034
Ctdspl	1.05	6.27E-03	0.034
Tnpo1	1.10	6.28E-03	0.034
Rab9	0.95	6.29E-03	0.034
Pias1	1.09	6.30E-03	0.034
Zbtb20	1.22	6.31E-03	0.034
Ndrg3	0.95	6.33E-03	0.034
Zfp217	1.07	6.35E-03	0.034
Mrps26	0.94	6.37E-03	0.034
Vsig10	1.25	6.38E-03	0.034
Nicn1	0.94	6.41E-03	0.034
Hoxb3	0.26	6.41E-03	0.034
Adprhl2	0.91	6.42E-03	0.035
Ppp1r7	0.92	6.45E-03	0.035
Pde6d	0.97	6.46E-03	0.035
Uggt2	1.11	6.47E-03	0.035
Atg5	0.92	6.47E-03	0.035
Atrx	1.08	6.48E-03	0.035
Ecd	0.92	6.49E-03	0.035
Fez2	0.88	6.49E-03	0.035
Cplx2	1.05	6.52E-03	0.035
Rrp12	0.83	6.54E-03	0.035
Vps41	0.94	6.58E-03	0.035

Eln	1.15	6.57E-03	0.035
Ube2h	1.05	6.57E-03	0.035
Dennd2a	1.05	6.58E-03	0.035
C1galt1c1	0.91	6.58E-03	0.035
Arpc4	0.91	6.59E-03	0.035
Rarg	1.10	6.61E-03	0.035
Zbtb6	1.06	6.61E-03	0.035
Iqgap1	1.03	6.61E-03	0.035
Tnfrsf23	1.33	6.61E-03	0.035
Cul2	0.91	6.62E-03	0.035
Ap1g2	1.18	6.65E-03	0.035
Mpi	0.93	6.65E-03	0.035
Dmxl1	1.05	6.67E-03	0.036
Pcdhb22	1.13	6.69E-03	0.036
Ick	1.01	6.70E-03	0.036
Ythdf3	1.04	6.74E-03	0.036
Bod1l	1.02	6.74E-03	0.036
Sez6l	1.11	6.74E-03	0.036
Tpp1	1.10	6.75E-03	0.036
Ece2	1.08	6.76E-03	0.036
Nsmf	0.92	6.76E-03	0.036
Magt1	1.04	6.76E-03	0.036
B130024G19Rik	1.36	6.78E-03	0.036
Mettl9	0.94	6.78E-03	0.036
Uqcr10	0.90	6.78E-03	0.036
Arhgef4	0.88	6.79E-03	0.036
P4htm	0.84	6.79E-03	0.036
Doc2g	1.34	6.82E-03	0.036
Ppp1r37	0.92	6.82E-03	0.036
Paip2	0.99	6.83E-03	0.036
Fgfrl1	0.86	6.84E-03	0.036
Ccdc115	0.93	6.86E-03	0.036
Sf3a2	0.96	6.86E-03	0.036
Dlg2	1.02	6.87E-03	0.036
Supt3	0.90	6.87E-03	0.036
Smyd2	0.95	6.88E-03	0.036
Gp1ba	1.23	6.89E-03	0.036
Fam120a	1.06	6.88E-03	0.036
Jmy	1.04	6.88E-03	0.036
C1d	0.90	6.90E-03	0.036
2410089E03Rik	1.02	6.90E-03	0.036
Rnd1	0.87	6.90E-03	0.036
Ablim1	1.02	6.90E-03	0.036
Penk	0.79	6.90E-03	0.036
Atg2b	0.97	6.93E-03	0.036
Gstp1	0.95	6.93E-03	0.036
Rbpms	1.17	6.93E-03	0.036
Cbfa2t3	1.01	6.92E-03	0.036
Itga6	1.07	6.94E-03	0.036

Mdm1	1.15	6.94E-03	0.036
Dis3l	0.89	6.95E-03	0.036
Cox11	0.90	6.95E-03	0.036
Trip11	1.07	6.96E-03	0.036
Cpt1a	1.08	6.97E-03	0.037
Cwf19l2	1.08	6.98E-03	0.037
Ankrd46	0.94	6.99E-03	0.037
Mrpl9	0.93	7.00E-03	0.037
Cramp1l	1.03	7.00E-03	0.037
Lrpap1	0.91	7.03E-03	0.037
Dusp11	1.07	7.03E-03	0.037
Rsbn1	1.03	7.04E-03	0.037
Cep19	0.92	7.05E-03	0.037
Cib2	0.94	7.06E-03	0.037
Syne2	1.08	7.07E-03	0.037
Zfc3h1	1.11	7.08E-03	0.037
Vdac2	0.95	7.11E-03	0.037
Alg14	0.94	7.11E-03	0.037
Brd1	1.07	7.13E-03	0.037
Clns1a	0.90	7.19E-03	0.037
Rpa1	0.91	7.22E-03	0.038
Zc3h11a	1.11	7.24E-03	0.038
Mrpl28	0.93	7.23E-03	0.038
Dclre1a	1.08	7.24E-03	0.038
Psma4	0.98	7.24E-03	0.038
Bckdk	0.89	7.25E-03	0.038
Vdac1	0.95	7.25E-03	0.038
Paics	0.93	7.26E-03	0.038
Pofut1	1.06	7.28E-03	0.038
Rabggta	0.87	7.29E-03	0.038
Mef2a	1.10	7.31E-03	0.038
Trerf1	1.09	7.31E-03	0.038
Prkcd	1.18	7.33E-03	0.038
Arid4b	1.09	7.35E-03	0.038
Mfsd5	0.92	7.36E-03	0.038
Jmjd6	0.95	7.38E-03	0.038
Ruvbl2	0.92	7.38E-03	0.038
Nudt3	0.96	7.38E-03	0.038
Pdhx	0.91	7.39E-03	0.038
Rpl23	0.97	7.40E-03	0.038
Scaf4	1.11	7.42E-03	0.038
Npas4	1.26	7.42E-03	0.038
Ddost	0.93	7.43E-03	0.038
Gnb5	0.88	7.44E-03	0.038
Dcaf4	0.91	7.45E-03	0.038
Map7	1.13	7.46E-03	0.038
Arsk	1.09	7.49E-03	0.039
Rasa2	1.03	7.50E-03	0.039
Gnb2	0.92	7.54E-03	0.039

Armc8	0.87	7.55E-03	0.039
Trpm4	1.10	7.56E-03	0.039
1700025G04Rik	0.97	7.57E-03	0.039
Phactr2	1.04	7.57E-03	0.039
Pklr	1.35	7.58E-03	0.039
Hnrnpul2	1.01	7.61E-03	0.039
Atp11c	1.13	7.61E-03	0.039
Ppif	0.93	7.62E-03	0.039
Gpr182	1.26	7.63E-03	0.039
Clmn	1.03	7.64E-03	0.039
Ankrd10	1.11	7.64E-03	0.039
Ift57	0.90	7.68E-03	0.039
Esco1	1.07	7.68E-03	0.039
Cachd1	1.14	7.69E-03	0.039
Smad7	1.05	7.71E-03	0.039
Pvr	1.09	7.72E-03	0.040
Ptprj	1.01	7.73E-03	0.040
Utp18	0.93	7.75E-03	0.040
Armc6	0.89	7.75E-03	0.040
Dyrk2	1.10	7.76E-03	0.040
Kdm6b	1.07	7.77E-03	0.040
Parp4	1.14	7.77E-03	0.040
Hmox2	0.96	7.77E-03	0.040
Wls	1.16	7.77E-03	0.040
Ddrgk1	0.95	7.79E-03	0.040
C130074G19Rik	1.21	7.81E-03	0.040
Tmem9b	0.91	7.80E-03	0.040
Sos2	1.02	7.82E-03	0.040
Plekha1	1.15	7.82E-03	0.040
Med16	0.86	7.84E-03	0.040
Col14a1	1.15	7.84E-03	0.040
Podxl2	0.93	7.84E-03	0.040
Hmgcr	0.88	7.86E-03	0.040
Ppara	1.15	7.86E-03	0.040
Tagap1	0.91	7.88E-03	0.040
Eif2b2	0.96	7.89E-03	0.040
Sh3kbp1	1.03	7.90E-03	0.040
Usp24	0.98	7.91E-03	0.040
Rnf130	1.14	7.93E-03	0.040
Itga5	1.12	7.94E-03	0.040
Tmem65	1.05	7.95E-03	0.040
Elmsan1	1.02	7.96E-03	0.040
Serac1	1.06	7.96E-03	0.040
Tctn2	0.89	7.98E-03	0.040
Mysm1	1.08	7.99E-03	0.040
Slmap	1.08	8.00E-03	0.040
Ddx24	0.92	8.01E-03	0.040
Lama5	1.04	8.01E-03	0.040
Zfp280c	1.15	8.01E-03	0.040

Eif4a2	0.95	8.02E-03	0.040
Sptssa	0.97	8.04E-03	0.041
Rps28	0.89	8.03E-03	0.041
Sdhaf2	0.90	8.03E-03	0.041
Dolpp1	0.91	8.04E-03	0.041
Rpusd2	1.04	8.04E-03	0.041
Adipor1	0.93	8.07E-03	0.041
Pank3	1.02	8.06E-03	0.041
Naga	1.13	8.06E-03	0.041
Pan3	1.09	8.06E-03	0.041
Lats2	1.08	8.08E-03	0.041
Morn2	0.89	8.08E-03	0.041
Akt3	1.05	8.08E-03	0.041
Pfkm	0.93	8.11E-03	0.041
Zfp758	1.12	8.12E-03	0.041
Ciapin1	0.88	8.12E-03	0.041
Rps13	0.92	8.13E-03	0.041
Mamdc2	1.25	8.15E-03	0.041
Nsmce4a	0.94	8.15E-03	0.041
Dctd	0.89	8.16E-03	0.041
Zfp180	0.93	8.16E-03	0.041
Pcmt1	0.93	8.17E-03	0.041
Btbd10	0.94	8.20E-03	0.041
Manbal	0.94	8.22E-03	0.041
Gpr26	1.15	8.22E-03	0.041
Dock4	1.04	8.23E-03	0.041
Pmpcb	0.94	8.25E-03	0.041
Fbxo9	0.89	8.27E-03	0.041
Rock2	1.03	8.29E-03	0.041
Fbxo11	1.09	8.29E-03	0.041
Ghitm	0.92	8.30E-03	0.041
Ckb	0.94	8.31E-03	0.041
Ogn	1.34	8.31E-03	0.041
Dfna5	0.91	8.31E-03	0.041
Rnf14	0.86	8.33E-03	0.042
Sdha	0.94	8.33E-03	0.042
Plce1	0.99	8.34E-03	0.042
Tfrc	1.16	8.35E-03	0.042
Prtg	1.13	8.35E-03	0.042
Sys1	0.90	8.37E-03	0.042
Sntb2	1.08	8.37E-03	0.042
Napepld	1.08	8.38E-03	0.042
4930570G19Rik	1.17	8.42E-03	0.042
Mtmr14	0.89	8.44E-03	0.042
Tbrg1	0.95	8.44E-03	0.042
Phtf2	1.03	8.45E-03	0.042
Rrp36	0.93	8.47E-03	0.042
Pfn2	0.95	8.47E-03	0.042
Aamdc	0.92	8.47E-03	0.042

Ramp3	0.84	8.49E-03	0.042
Ccdc32	0.93	8.50E-03	0.042
Slc16a14	1.17	8.50E-03	0.042
Zfp334	1.03	8.50E-03	0.042
H6pd	1.08	8.51E-03	0.042
Phip	1.09	8.51E-03	0.042
Acad9	0.89	8.54E-03	0.042
Tmem5	0.94	8.54E-03	0.042
Gm20751	1.50	8.57E-03	0.042
Nme3	0.91	8.57E-03	0.042
Phkg2	0.92	8.57E-03	0.042
Eya4	1.21	8.63E-03	0.042
Tspan13	0.94	8.62E-03	0.042
Tbc1d7	0.96	8.62E-03	0.042
Rabl3	0.86	8.62E-03	0.042
Tbl1xr1	1.01	8.63E-03	0.042
Vps13d	0.96	8.63E-03	0.042
Steap2	0.98	8.61E-03	0.042
Vamp5	1.43	8.61E-03	0.042
Rpn1	0.92	8.62E-03	0.042
Cmip	1.06	8.60E-03	0.042
Maml2	1.03	8.60E-03	0.042
Ndst3	1.06	8.66E-03	0.043
Ppic	1.24	8.67E-03	0.043
Zic2	1.15	8.68E-03	0.043
Grik2	1.09	8.69E-03	0.043
Kbtbd7	1.04	8.69E-03	0.043
Snn	0.92	8.73E-03	0.043
Smc5	1.08	8.73E-03	0.043
Rabepk	0.89	8.75E-03	0.043
Cdc14a	1.14	8.79E-03	0.043
AY358078	0.91	8.83E-03	0.043
Serp2	0.94	8.83E-03	0.043
Emc1	1.02	8.83E-03	0.043
Rnaset2b	0.90	8.86E-03	0.043
Fkbp9	1.10	8.86E-03	0.043
Prdm2	1.02	8.87E-03	0.043
Commd8	0.91	8.87E-03	0.043
Atp5g3	0.94	8.93E-03	0.044
Sik2	1.10	8.94E-03	0.044
Rpl12	0.94	8.95E-03	0.044
4932438A13Rik	1.00	8.97E-03	0.044
Mrpl50	0.95	8.98E-03	0.044
Commd7	0.90	9.00E-03	0.044
Akap2	1.07	9.00E-03	0.044
Stmn2	0.93	9.01E-03	0.044
Sncg	0.54	9.02E-03	0.044
Ftx	1.10	9.03E-03	0.044
Tfpi	1.13	9.03E-03	0.044

Prps1l3	0.92	9.06E-03	0.044
Lamp5	0.44	9.08E-03	0.044
Pcx	0.84	9.10E-03	0.044
Emx2	1.14	9.10E-03	0.044
Serf2	0.89	9.10E-03	0.044
Rgs5	1.11	9.12E-03	0.044
Ncoa6	1.07	9.12E-03	0.044
Fastk	0.92	9.12E-03	0.044
Rexo2	0.95	9.12E-03	0.044
Adamtsl3	1.17	9.13E-03	0.044
Chd8	1.01	9.14E-03	0.044
Cmpk1	0.93	9.14E-03	0.044
Prkag1	0.92	9.18E-03	0.044
Dbx2	0.63	9.19E-03	0.044
Enpp1	1.40	9.22E-03	0.045
Tek	1.07	9.22E-03	0.045
Rnasel	1.11	9.25E-03	0.045
Aldoart1	0.83	9.28E-03	0.045
Tusc3	0.94	9.27E-03	0.045
Map4k2	1.17	9.28E-03	0.045
Stk16	0.93	9.30E-03	0.045
Pot1b	1.15	9.30E-03	0.045
Magi1	1.02	9.30E-03	0.045
Taf12	0.96	9.31E-03	0.045
Zfp148	1.02	9.32E-03	0.045
Mast4	1.11	9.34E-03	0.045
Srek1	1.13	9.35E-03	0.045
Ppp1r1a	0.92	9.35E-03	0.045
Efhd2	0.90	9.35E-03	0.045
Kdm5a	1.03	9.35E-03	0.045
Gbp7	1.18	9.39E-03	0.045
Limk1	0.92	9.40E-03	0.045
Poll	0.85	9.42E-03	0.045
Zfp809	1.17	9.43E-03	0.045
Dab1	1.11	9.44E-03	0.045
Ddx39	0.87	9.44E-03	0.045
Rnf32	0.91	9.46E-03	0.045
Mmp14	1.13	9.47E-03	0.045
Ccdc171	1.17	9.47E-03	0.045
Card6	1.18	9.48E-03	0.045
Kif21a	1.03	9.49E-03	0.046
Atp8a1	1.04	9.52E-03	0.046
Abhd11	0.94	9.55E-03	0.046
Tub	1.01	9.56E-03	0.046
Cadm1	1.08	9.56E-03	0.046
Elp6	0.86	9.56E-03	0.046
Bod1	0.94	9.57E-03	0.046
Ergic1	0.95	9.57E-03	0.046
Angptl6	0.85	9.59E-03	0.046

Lin52	0.90	9.62E-03	0.046
Pdhb	0.97	9.62E-03	0.046
Abca5	1.04	9.63E-03	0.046
Xrcc5	0.87	9.66E-03	0.046
Bad	0.92	9.66E-03	0.046
Fzd5	1.11	9.68E-03	0.046
Ppp1r3f	0.85	9.68E-03	0.046
Fzd8	1.10	9.73E-03	0.046
Tmem2	1.07	9.73E-03	0.046
L3mbtl2	0.89	9.74E-03	0.046
Tomt	0.91	9.76E-03	0.046
Tspan3	0.93	9.77E-03	0.046
Fam227a	1.06	9.77E-03	0.047
Bax	0.95	9.79E-03	0.047
Adarb2	1.04	9.80E-03	0.047
Dgka	1.14	9.82E-03	0.047
Thra	0.95	9.82E-03	0.047
Sap30bp	0.93	9.82E-03	0.047
Thbs3	1.19	9.82E-03	0.047
Hist1h2bc	0.88	9.85E-03	0.047
1110008P14Rik	0.90	9.85E-03	0.047
Rab2a	0.97	9.85E-03	0.047
Ociad1	0.92	9.84E-03	0.047
Sirt1	1.12	9.86E-03	0.047
Gnpda1	1.11	9.86E-03	0.047
1700028K03Rik	1.25	9.87E-03	0.047
Eml4	1.05	9.87E-03	0.047
Ttc28	1.13	9.87E-03	0.047
0610009O20Rik	0.90	9.89E-03	0.047
Glud1	0.90	9.90E-03	0.047
Fuca1	0.90	9.91E-03	0.047
Znrf3	1.02	9.92E-03	0.047
Pten	0.99	9.95E-03	0.047
Slc39a14	1.03	9.97E-03	0.047
Cdc37l1	1.08	9.98E-03	0.047
Slc25a27	1.08	1.00E-02	0.047
Ccdc14	1.07	1.00E-02	0.047
Nrbp2	1.16	1.00E-02	0.047
Impad1	1.04	1.01E-02	0.047
Slc38a4	1.22	1.01E-02	0.047
Hoxa3	0.24	1.01E-02	0.047
Btaf1	1.10	1.01E-02	0.048
Katnb1	0.90	1.01E-02	0.048
Ubtd1	0.86	1.01E-02	0.048
Il6ra	1.26	1.01E-02	0.048
Eogt	1.12	1.01E-02	0.048
Mrpl48	0.88	1.01E-02	0.048
4930447C04Rik	1.32	1.01E-02	0.048
Nmt1	0.95	1.02E-02	0.048

Cycs	0.87	1.02E-02	0.048
Kat6a	1.03	1.02E-02	0.048
Bphl	0.92	1.02E-02	0.048
Vmp1	0.90	1.02E-02	0.048
Enpep	1.36	1.02E-02	0.048
Ndp	0.83	1.02E-02	0.048
Polr1d	0.93	1.03E-02	0.048
Plxnc1	1.04	1.03E-02	0.048
Shisa6	1.03	1.03E-02	0.048
Dbn1	0.94	1.03E-02	0.048
Rmnd5a	1.00	1.03E-02	0.048
Atn1	1.06	1.03E-02	0.048
Nsmce1	0.91	1.03E-02	0.048
Rpl18a	0.96	1.03E-02	0.048
Wisp1	1.37	1.03E-02	0.048
Phpt1	0.95	1.04E-02	0.048
Ncaph2	0.91	1.04E-02	0.048
Ggta1	1.09	1.04E-02	0.048
Pi4k2b	1.12	1.04E-02	0.048
Cend1	0.83	1.04E-02	0.048
Ccdc15	1.13	1.04E-02	0.048
Nsfl1c	0.97	1.04E-02	0.048
Ptpdc1	1.05	1.04E-02	0.049
Trim12c	1.13	1.04E-02	0.049
Rpe	0.88	1.05E-02	0.049
Pole4	0.93	1.05E-02	0.049
Scn9a	1.10	1.05E-02	0.049
Kcnh5	1.14	1.05E-02	0.049
Cadm2	1.05	1.05E-02	0.049
Kidins220	1.02	1.05E-02	0.049
Adamts1	1.07	1.05E-02	0.049
H2-T23	1.35	1.05E-02	0.049
Ccdc24	1.19	1.05E-02	0.049
Zscan29	1.08	1.05E-02	0.049
Samd4	1.07	1.05E-02	0.049
Efna5	1.07	1.05E-02	0.049
Tmbim4	0.95	1.05E-02	0.049
Avl9	1.02	1.06E-02	0.049
Helz2	1.10	1.06E-02	0.049
Kcnh4	1.09	1.06E-02	0.049
Abca4	1.20	1.06E-02	0.049
Ift20	0.99	1.06E-02	0.049
Mfap3	1.01	1.06E-02	0.049
Tgfb3	1.12	1.06E-02	0.049
Angpt2	1.21	1.06E-02	0.049
Fanca	1.18	1.06E-02	0.049
Hs3st3a1	1.25	1.07E-02	0.049
Hgs	0.96	1.07E-02	0.049
Mob2	0.92	1.07E-02	0.049

Tfap2d	1.33	1.07E-02	0.050
Polr3d	0.89	1.07E-02	0.050
Bicd1	1.07	1.07E-02	0.050
Tnk2	1.04	1.08E-02	0.050
Bche	1.17	1.08E-02	0.050
Ryr1	0.99	1.08E-02	0.050
Aig1	0.93	1.08E-02	0.050
Fkbp2	0.90	1.08E-02	0.050
Mrs2	1.13	1.09E-02	0.050

Table S2 - All significantly up-regulated KEGG pathways in Fetal Brain & their associated genes

KEGG pathway	Net Enrichment Score	# significant/total genes	Padj	Gene Name
Complement and coagulation cascades	2.73	13/17	0.0026	*A2m, C3, Cd46, Cfb, Cfh, F13a1, Fga, Fgb, Fgg, Serpind1, Tfpi, Thbd, Vwf*
Protein digestion and absorption	2.65	26/31	0.0026	*Atp1a2, Col11a1, Col12a1, Col14a1, Col15a1, Col16a1, Col18a1, Col1a1, Col1a2, Col23a1, Col25a, Col27a1, Col2a1, Col3a1, Col4a1, Col4a2, Col4a5, Col4a6, Col5a1, Col5a2, Col6a1, Col6a2, Col6a3, Dpp4, Eln, Slc7a8*
Platelet activation	2.55	16/21	0.0026	*Adcy6, Col1a1, Col1a2, Col3a1, Fga, Fgb, Ggg, Gp1ba, Gucy1a2, Itga2, Itgb3, Itpr3, Mylk, Rock1, Snap23, Vwf*
ECM-receptor interaction	2.51	34/43	0.0026	*Agrn, Col1a1, Col1a2, Col2a1, Col4a1, Col4a2, Col4a5, Col4a6, Col6a1, Col6a2, Col6a3, Fn1, Frem1, Frem2, Gp1ba, Hspg2, Itga1, Itga11, Itga2, Itga5, Itga6, Itgb3, Lama1, Lama2, Lama4, Lamb1, Lamb2, Lamc1, Lamc3, Reln, Thbs2, Thbs3, Tnr, Vwf*
Focal adhesion	2.35	37/48	0.0026	*Col1a1, Col1a2, Col2a1, Col4a1, Col4a2, Col4a5, Col4a6, Col6a1, Col6a2, Col6a3, Flnb, Flnc, Fn1, Igf1r, Itga1, Itga11, Itga2, Itga5, Itgb3, Kdr, Lama1, Lama2, Lamb1, Lamb2, Lamc1, Lamc3, Mylk, Pdgfrb, Pxn, Reln, Rock1, Thbs2, Thbs3, Tnr, Vav3, Vegfa, Vwf*
Hippo signaling pathway	2.34	28/53	0.0026	*Afp, Amot, Apc, Axin2, Bmp5, Fmp6, Bmp7, Bmpr2, Cdh1, Dlg1, Dvl3, Fzd1, Fzd3, Fzd5, Fzd7, Fzd8, Gsk3b, Lats2, Mob1b, Smad3, Tcf7l1, Tead1, Tgfb3, Tgfbr2, Trp73, Wnt2b, Wwtr1, Yap1*
AGE-RAGE signaling pathway in diabetic complications	2.30	15/19	0.0026	*Col1a1, Col1a2, Col3a1, Col4a1, Col4a2, Col4a5, Col4a6, Egr1, Fn1, Mmp2, Prkcd, Smad3, Tgfb3, Tgfbr2, Thbd*
Cytokine-cytokine receptor interaction	2.26	15/35	0.0026	*Acvr1c, Bmp5, Bmp6, Bmp7, Bmpr2, Csf1, Csf1r, Cxcl12, Gdf11, Il17ra, Il6ra, Inha, Lepr, Tgfb3, Tgfbr2*
Staphylococcus aureus infection	2.25	4/6	0.0071	*C3, Cfb, Cfh, Fgg*
Proteoglycans in cancer	2.25	31/44	0.0026	*Ank2, Ank3, Arhgef1, Cbl, Col1a1, Col1a2, Dcn, Erbb4, Flnb, Flnc, Fn1, Fzd1, Fzd3, Fzd5, Fzd7, Fzd8, Gpc3, Hspg2, Igf1r, Ifg2, Itga2, Itga5, Itgb3, Itpr3, Kdr, Mmp2, Pxn, Rock1, Vav3, Vegfa, Wnt2b*
TGF-beta signaling pathway	2.25	23/42	0.0026	*Acvr1b, Acvr1c, Acvr2a, Bmp5, Bmp6, Bmp7, Bmpr2, Chrd, Crebbp, Dcn, Ep300, Fbn1, Fmod, Neo1, Rock1, Smad3, Smad6, Smad7, Smurf2, Sp1, Tgfb3, Tgfbr2, Thsd4*
Amoebiasis	2.23	15/17	0.0026	*Col1a1, Col1a2, Col3a1, Col4a1, Col4a2, Col4a5, Col4a6, Fn1, Lama1, Lama2, Lamb1, Lamb2, Lamc1, Lamc3, Tgfb3*
Neutrophil extracellular trap formation	2.05	6/7	0.0026	*C3, Fga, Fgb, Fgg, Gp1ba, Vwf*
Human papillomavirus infection	1.99	46/68	0.0026	*Axin2, Col1a1, Col1a2, Col2a1, Col4a1, Col4a2, Col4a5, Col4a6, Col6a1, Col6a2, Col6a3, Creb5, Crebbp, Dvl3, Eif2ak2, Fn1, Fzd1, Fzd3, Fzd5, Fzd7, Fzd8, H2-M5, H2-T23, Itga1, Itga11, Itga2, Itga5, Itgb3, Jag1, Lama1, Lama2, Lamb1, Lamb2, Lamc1, Lamc3, Notch2, Pdgfrb, Pxn, Reln, Tcf7l1, Thbs2, Thbs3, Tnr, Vtn, Vwf, Wnt2b*
Adherens junction	1.98	12/22	0.016	*Cdh1, Crebbp, Ctnnd1, Ep300, Igf1r, Insr, Ptprb, Smad3, Sorbs1, Tcf7l1, Tgfbr1, Tjp1*
Basal cell carcinoma	1.97	12/26	0.016	*Apc, Axin2, Dvl3, Fzd1, Fzd3, Fzd5, Fzd7, Fzd8, Gli3, Gsk3b, Tcf7l1, Wnt2b*

Wnt signaling pathway	1.95	24/53	0.0026	*Apc, Axin2, Crebbp, Daam1, Dkk2, Dvl3, Ep300, Fzd1, Fzd3, Fzd5, Fzd7, Fzd8, Gsk3b, Lrp5, Lrp6, Nfatc2, Nfatc4, Ror2, Serpinf1, Smad3, Tcf7l1, Vangl2, Wisp1, Wnt2b*
Hematopoietic cell lineage	1.91	11/21	0.033	*Anpep, Csf1, Csf1r, Gp1ba, Il6ra, Itga1, Itga2, Itga5, Itga6, Itgb3, Tfrc*
PI3K-Akt signaling pathway	1.90	47/69	0.0026	*Angpt2, Col1a1, Col1a2, Col2a1, Col4a1, Col4a2, Col4a5, Col4a6, Col6a1, Col6a2, Col6a3, Creb5, Csf1, Csf1r, Efna5, Erbb4, Fgfr2, Fn1, Foxo3, Igf1r, Igf2, Il6ra, Insr, Itga1, Itga11, Itga2, Itga5, Itga6, Itgb3, Kdr, Lama1, Lama2, Lama4, Lamb1, Lamb2, Lamc1, Lamc3, Lpar1, Mtcp1, Pdgfrb, Reln, Tek, Thbs2, Thbs3, Tnr, Vtn, Vwf*
Arrhythmogenic right ventricular cardiomyopathy	1.87	15/28	0.024	*Cacna1c, Cacna1d, Cacna2d1, Cacnb4, Dsp, Gja1, Itga1, Itga11, Itga2, Itga5, Itga6, Itgb3, Lama1, Lama2, Tcf7l1*
Coronavirus disease - COVID-19	1.86	9/15	0.0026	*C3, Cfb, Eif2ak2, F13a1, Fga, Fgb, Fgg, Il6ra, Vwf*
Signaling pathways regulating pluripotency of stem cells	1.85	22/43	0.015	*Acvr1b, Acvr1c, Apc, Axin2, Bmpr2, Dvl3, Fgf2, Fzd1, Fzd3, Fzd5, Fzd7, Fzd8, Gsk3b, Igf1r, Jarid2, Pcgf6, Pik3r1, Pik3r3, Rest, Smad3, Wnt2b, Zfhx3*
Cell adhesion molecules	1.81	19/37	0.024	*Cadm1, Cdh1, Cdh4, Cdh5, Cldn1, Cldn11, Cldn19, Glg1, H2-M5, H2-T23, Itga6, Itgav, L1cam, Ncam1, Neo1, Nfasc, Nrcam, Nrxn1, Pvr*
MicroRNAs in cancer	1.70	29/63	0.024	*Abcb1a, Apc, Apc2, Bcl2, Bmpr2, Crebbp, Cyp1b1, Dicer1, Dnmt3b, Efna5, Ep300, Fzd3, Gls, Itga5, Itgb3, Mdm4, Mmp16, Notch2, Notch3, Pdgfrb, Pik3r1, Pik3r3, Reck, Rock1, Sirt1, Sos1, Tnr, Vegfa, Zeb1*
Relaxin signaling pathway	1.67	10/14	0.048	*Col1a1, Col1a2, Col3a1, Col4a1, Col4a2, Col4a5, Col4a6, Creb5, Mmp2, Tgfbr2*
Gastric cancer	1.67	23/45	0.039	*Abcb1a, Apc, Axin2, Braf, Cdh1, Dvl3, Fgfr2, Fzd1, Fzd3, Fzd5, Fzd7, Fzd8, Gab1, Gsk3b, Lrp5, Lrp6, Pik3r1, Pik3r3, Smad3, Tcf7l1, Tgfb3, Tgfbr2, Wnt2b*
Pathways in cancer	1.44	63/120	0.024	*Adcy6, Adcy9, Apc, Arhgef1, Axin2, Braf, Cbl, Cdh1, Col4a1, Col4a2, Col4a5, Col4a6, Crebbp, Csf1r, Cxcl12, Dcc, Dvl3, Ep300, Epas1, Fgfr2, Fn1, Fzd1, Fzd3, Fzd5, Fzd7, Fzd8, Gli3, Gnaq, Gsk3b, Igf1r, Igf2, Il6ra, Itga2, Itga6, Jag1, Kit, Lama1, Lama2, Lama4, Lamb1, Lamb2, Lamc1, Lamc3, Lpar1, Lrp5, Lrp6, Mmp2, Notch2, Notch3, Pdgfrb, Pik3r1, Pik3r3, Pml, Rock1, Runx1t1, Slc2a1, Smad3, Sp1, Tcf7l1, Tgfb3, Tgfbr2, Wnt2b, Zbtb16*

Table S3- All significantly down-regulated KEGG pathways in Fetal Brain and associated genes.

KEGG pathway	Net Enrichment Score	# significant/total genes	Padj	Gene Name
Oxidative phosphorylation	-2.61	63/69	0.0026	*Atp5a1, Atp5b, Atp5c1, Atp5f1, Atp5g1, Atp5g3, Atp5j2, Atp5k, Atp5o, Atp6v0d1, Atp6v0e2, Atp6v1b2, Atp6v1f, Atp6v1h, Cox10, Cox11, Cox17, Cox5a, Cox5b, Cox6a1, Cox6b1, Cox7a2, Cox7a2l, Cox7b, Cox8a, Cyc1, Ndufa10, Ndufa13, Ndufa2, Ndufa3, Ndufa6, Ndufa7, Ndufa8, Ndufa9, Ndufb10, Ndufb11, Ndufb2, Ndufb4, Ndufb5, Ndufb6, Ndufb7, Ndufb8, Ndufb9, Ndufc1, Ndufc2, Ndufs4, Ndufs6, Ndufs7, Ndufs8, Ndufv1, Ndufv2, Ndufv3, Ppa1, Ppa2, Sdhb, Sdhc, Sdhd, Uqcr10, Uqcr11, Uqcrc1, Uqcrc2, Uqcrfs1, Uqcrq*
Ribosome	-2.46	74/94	0.0026	*Mrpl10, Mrpl11, Mrpl12, Mrpl13, Mrpl14, Mrpl15, Mrpl18, Mrpl19, Mrpl2, Mrpl20, Mrpl22, Mrpl27, Mrpl28, Mrpl3, Mrpl33, Mrpl34, Mrpl9, Mrps10, Mrps11, Mrps12, Mrps17, Mrps18a, Mrps21, Mrps6, Mrps7, Rpl11, Rpl12, Rpl13, Rpl13a, Rpl14, Rpl15, Rpl18, Rpl22l1, Rpl27, Rpl27a, Rpl28, Rpl29, Rpl3, Rpl30, Rpl31, Rpl35, Rpl36, Rpl36a, Rpl37, Rpl38, Rpl39, Rpl4, Rpl41, Rpl6, Rpl8, Rplp0, Rplp1, Rps10, Rps13, Rps15, Rps16, Rps17, Rps18, Rps2, Rps20, Rps21, Rps23, Rps24, Rps27a, Rps28, Rps29, Rps3, Rps5, Rps6, Rps7, Rps8, Rps9, Rpsa*
Parkinson disease	-2.30	100/133	0.0026	*Aft4, Atp5a1, Atp5b, Atp5c1,Atp5d, Atp5g1, Atp5f1, Atp5g3, Atp5h, Atp5o, Bax, Cox4i1, Cox5a, Cox5b, Cox6a1, Cox6b1, Cox7a2, Cox7a2l, Cox7b, Cox8a, Cyc1, Cycs, Daxx, Ndufa10, Ndufa13, Ndufa2, Ndufa3, Ndufa6, Ndufa7, Ndufa8, Ndufa9, Ndufb10, Ndufb11, Ndufb2, Ndufb5, Ndufb6, Ndufb7, Ndufb8, Ndufb9, Ndufc1, Ndufc2, Ndufs2, Ndufs4, Ndufs6, Ndufs7, Ndufs8, Ndufv1, Ndufv2, Ndufv3, Park7, Ppif, Psma6, Psma7, Psmb1, Psmb4, Psmb5, Psmb6, Psmb7, Psmc1, Psmc2, Psmc3, Psmc4, Psmc5, Psmd1, Psmd12, Psmd13, Psmd2, Psmd3, Psmd6, Psmd7, Psmd8, Psmd9, Rps27a, Sdha, Sdhb, Sdhc, Sdhd, Sept5, Trap1, Tuba1a, Tuba1b, Tuba1c, Tubb3, Tubb4b, Tubb5, Txn1, Txn2, Uba52, Ubb, Uchl1, Uqcr10, Uqcr11, Uqcrc1, Uqcrc2, Uqcrfs1, Uqcrq, Vdac1, Vdac2, Vdac3, Xbp1*
Carbon metabolism	-2.12	36/52	0.0026	*Acat2, Acat3, Aco2, Aldoart1, Aldoc, Echs1, Fh1, Gcsh, Glud1, Got1, Hadha, Hibch, Idh3a, Idh3b, Mcee, Mdh1, Mdh2, Pccb, Pcx, Pdha1, Pfkl, Pgam1, Pgk1, Pgls, Prps1, Prps1l3, Psph, Rpe, Sdhb, Sdhc, Sdhd, Sucla2, Suclg1, Taldo1, Tkt, Tpi1*
Thermogenesis	-2.10	61/81	0.0026	*Actg1, Akt1s1, Atp5a1, Atp5b, Atp5c1, Atp5f1, Atp5g1, Atp5h, Atp5j2, Atp5k, Coa3, Cox10, Cox11, Cox16, Cox4i1, Cox5a, Cox5b, Cox6a1, Cox7a2, Cox7a2l, Cyc1, Ndufa10, Ndufa13, Ndufa2, Ndufa3, Ndufa6, Ndufa7, Ndufa9, Ndufaf2, Ndufaf3, Ndufaf5, Ndufb10, Ndufb11, Ndufb2, Ndufb5, Ndufb6, Ndufb7, Ndufb8, Ndufb9, Ndufc1, Ndufc2, Ndufs2, Ndufs4, Ndufs6, Ndufs7, Ndufs8, Ndufv1, Ndufv2, Ndufv3, Prkag1, Sdhb, Sdhc, Sdhd, Smarcd3, Uqcr10, Uqcr11, Uqcrc1, Uqcrc2, Uqcrfs1, Uqcrq*
Propanoate metabolism	-2.05	11/26	0.018	*Acat2, Acat3, Bckdhb, Echs1, Hadha, Hibch, Ldhb, Mcee, Pccb, Sucla2, Suclg1*
TCA cycle	-1.99	16/24	0.024	*Aco2, Dlst, Fh1, Idh1, Idh3a, Idh3b, Mdh1, Mdh2, Pcx, Pdha1, Sdha, Sdhb, Sdhc, Sdhd, Sucla2, Suclg1*
Amyotrophic lateral sclerosis	-1.97	80/104	0.0026	*Actg1, Actr10, Actr1a, Atf4, Atp5a1, Atp5b, Atp5c1, Atp5f1, Atp5g1, Atp5h, Bad, Cox4i1, Cox5a, Cox5b, Cox6a1, Cox6b1, Cox7a2, Cox7a2l, Cyc1, Cycs, Daxx, Dctn3, Gabarap, Gpx1, Ndufa10, Ndufa13, Ndufa2, Ndufa3, Ndufa6, Ndufa7, Ndufa9, Ndufb10, Ndufb11, Ndufb2, Ndufb5, Ndufb6, Ndufb7, Ndufb8, Ndufb9, Ndufc1, Ndufc2, Ndufs2, Ndufs4, Ndufs6, Ndufs7, Ndufs8, Ndufv1, Ndufv2, Ndufv3, Psma6, Psma7, Psmb1, Psmb4, Psmb5, Psmb6, Psmb7, Psmc2, Psmc3, Psmc4, Psmc5, Psmd6, Psmd8, Psmd9, Sdhb, Sdhc, Sdhd, Sec13, Sigmar1, Sqstm1, Tomm40, Tomm40l, Tuba1c, Tubb4b, Uqcr10, Uqcr11, Uqcrc1, Uqcrc2, Uqcrfs1, Uqcrq, Xbp1*
Biosynthesis of cofactors	-1.94	21/51	0.0044	*Adsl, Ak1, Akr1a1, Cmpk1, Coq2, Coq6, Coq7, Cox10, Ggh, Lias, Mocs2, Mpi, Mthfd2, Nme1, Nme3, Pank1, Phospho2, Pmm1, Psat1, Umps, Uros*

Huntington disease	-1.92	105/139	0.0026	*Actr10, Actr1a, Ap2a1, Ap2m1, Ap2s1, Atp5a1, Atp5b, Atp5c1, Atp5d, Atp5f1, Atp5g1, Atp5g3, Atp5h, Atp5o, Bax, Clta, Cox4i1, Cox5a, Cox5b, Cox6a1, Cox6b1, Cox7a2, Cox7a2l, Cox7b, Cox8a, Cyc1, Cycs, Dctn2, Dctn3, Dctn5, Gpx1, Ift57, Ndufa10, Ndufa13, Ndufa2, Ndufa3, Ndufa6, Ndufa7, Ndufa8, Ndufa9, Ndufb10, Ndufb11, Ndufb2, Ndufb5, Ndufb6, Ndufb7, Ndufb8, Ndufb9, Ndufc1, Ndufc2, Ndufs2, Ndufs4, Ndufs6, Ndufs7, Ndufs8, Ndufv1, Ndufv2, Ndufv3, Polr2d, Polr2e, Polr2f, Polr2g, Polr2h, Polr2i, Ppif, Psma6, Psma7, Psmb1, Psmb4, Psmb5, Psmb6, Psmb7, Psmc1,Psmc2, Psmc3, Psmc4, Psmc5, Psmd1, Psmd12, Psmd13, Psmd2, Psmd3, Psmd6, Psmd7, Psmd8, Psmd9, Sdha, Sdhb, Sdhc, Sdhd, Tuba1a, Tuba1b, Tuba1c, Tubb3, Tubb4b, Tubb5, Uqcr10, Uqcr11, Uqcrc1, Uqcrc2, Uqcrfs1, Uqcrq, Vdac1, Vdac2, Vdac3*
Biosynthesis of amino acids	-1.91	22/44	0.016	*Aco2, Aldoart1, Aldoc, Asns, Got1, Idh1, Idh3a, Idh3b, Pcx, Pfkl, Pfkm, Pgam1, Pgk1, Prps1, Prsp1l3, Psat1, Psph, Pycrl, Rpe, Taldo1, Tkt, Tpi1*
Aminoacyl-tRNA biosynthesis	-1.83	17/32	0.038	*Aars, Aars2, Cars, Dars, Farsa, Farsb, Gars, Hars, Kars, Mars, Nars, Sars, Sars2, Tars2, Wars, Yars, Yars2*
Folate biosynthesis	-1.82	3/11	0.035	*Ggh, Mocs2, Qdpr*
Prion disease	-1.81	77/94	0.0026	*Atf4, Atf6b, Atp5a1, Atp5b, Atp5c1, Atp5f1, Atp5g1, Atp5h, Atp5o, Bad, Cox4i1, Cox5a, Cox5b, Cox6a1, Cox6b1, Cox7a2, Cox7a2l, Cox7b, Cox, Cyc1, Cycs, Hspa8, Ndufa10, Ndufa13, Ndufa2, Nduf3, Ndufa6, Ndufa7, Ndufa8, Ndufa9, Ndufb10, Ndufb11, Ndufb2, Ndufb5, Ndufb6, Ndufb7, Ndufb8, Ndufb9, Ndufc1, Ndufc2, Ndufs2, Ndufs4, Ndufs6, Ndufs7, Ndufs8, Ndufv1, Ndufv2, Ndufv3, Ppif, Psma6, Psma7, Psmb1, Psmb4, Psmb5, Psmb6, Psmb7, Psmc2, Psmc3, Psmc4, Psmc5, Psmd1, Psmd6, Psmd8, Psmd9, Sdhb, Sdhc, Sdhd, Stip1, Tuba1c, Tubb4b, Uqcr10, Uqcr11, Uqcrc1, Uqcrc2, Uqcrfs1, Uqcrq, Vdac3*
Steriod biosynthesis	-1.80	7/11	0.038	*Cyp51, Dhcr24, Dhcr7, Fdt1, Hsd17b7, Nsdhl, Sqle*
Non-alcoholic fatty liver disease	-1.75	47/51	0.012	*Atf4, Cdc42, Cox4i1, Cox5a, Cox5b, Cox6a1, Cox6b1, Cox7a2, Cox7a2l, Cyc1, Cycs, Ndufa10, Ndufa13, Ndufa2, Ndufa3, Ndufa6, Ndufa7, Ndufa9, Ndufb10, Ndufb11, Ndufb2, Ndufb5, Ndufb6, Ndufb7, Ndufb8, Ndufb9, Ndufc1, Ndufc2, Ndufs2, Ndufs4, Ndufs6, Ndufs8, Ndufv1, Ndufv2, Ndufsv3, Prkag1, Sdhb, Sdhc, Sdhd, Uqcr10, Uqcr11, Uqcrc1, Uqcrc2, Uqcrfs1, Uqcrq, Xbp1*
Pathways of neurodegeneration	-1.74	93/143	0.0026	*Actr10, Actr1a, Atf4, Atp5a1, Atp5b, Atp5c1, Atp5f1, Atp5g1, Atp5h, Atp5o, Bad, Cox4i1, Cox5a, Cox5b, Cox6a1, Cox6b1, Cox7a2, Cox7a2l, Cox7b, Cox8a, Cyc1, Cycs, Daxx, Dctn3, Gabarap, Gpx1, Hsd17b10, Ift57, Ndufa10, Ndufa3, Ndufa2, Ndufa3, Ndufa6, Ndufa7, Ndufa8, Ndufa9, Ndufc1, Ndufc2, Ndufs2, Ndufs4, Ndufs6, Ndufs7, Ndufs8, Ndufv1, Ndufv2, Ndufv3, Park7, Ppif, Psma6, Psma7, Psmb1, Psmb4, Psmb5, Psmb6, Psmb7, Psmc2, Psmc3, Psmc4, Psmc5, Psmd1, Psmd6, Psmd8, Psmd9, Rab8a, Rps27a, Sdhb, Sdhc, Sdhd, Septin5, Sigmar1, Sqstm1, Tomm40, Tom40l, Trap1, Tuba1c, Tubb4b, Ubb, Uqcr10, Uqcr11, Uqcrc1, Uqcrc2, Uqcrfs1, Uqcrq, Vdac3, Xbp1*
Alcoholism	-1.69	10/48	0.024	*Atf4, Atf6b, Gnb2, Gnb5, H2aj, Hist1h2bc, Hist3h2a, Hist3h2ba, Pp1ca, Psmb5*
Peroxisome	-1.68	9/40	0.050	*Amacr, Idh1, Mpv17, Mpv17l2, Mvk, Nudt19, Pex19, Prdx5, Scp2*
Necroptosis	-1.61	12/31	0.042	*Chmp3, Chmp6, Chmp7, Faf1, Glud1, H2afj, Hist3h2a, Pgam5, Ppia, Smpd1, Sqstm1, Vdac3*
Retrograde endocannabinoid signaling	-1.60	27/40	0.040	*Gnb2, Gnb5, Ndufa10, Ndufa13, Ndufa2, Nduf3, Ndufa6, Ndufa7, Ndufa9, Ndufb10, Ndufb11, Ndufb2, Ndufb5, Ndufb6, Ndufb7, Ndufb8, Ndufb9, Ndufc1, Ndufc2, Ndufs2, Ndufs4, Ndufs6, Ndufs7, Ndufs8, Ndufv1, Ndufv2, Ndufv3*

Alzheimer disease	-1.57	78/110	0.0064	*Apbb1, Atf4, Atp5a1, Atp5b, Atp5c1, Atp5g1, Atp5h, Atp5o, Atp5pb, Bad, Cox4i1, Cox5a, Cox5b, Cox6a1, Cox6b1, Cox7a2, Cox7a2l, Cox7b, Cox8a, Cyc1, Cycs, Hsd17b10, Ndufa10, Ndufa13, Ndufa2, Ndufa3, Ndufa6, Ndufa7, Ndufa8, Ndufa9, Ndufb10, Ndufb11, Ndufb2, Ndufb5, Ndufb6, Ndufb7, Ndufb8, Ndufb9, Ndufc1, Ndufc2, Ndufs2, Ndufs4, Ndufs6, Ndufs7, Ndufs8, Ndufv1, Ndufv2, Ndufv3, Ppif, Psenen, Psma6, Psma7, Psmb1, Psmb4, Psmb5, Psmb6, Psmb7, Psmc2, Psmc3, Psmc4, Psmc5, Psmd1, Psmd6, Psmd8, Psmd9, Sdhb, Sdhc, Sdhd, Tuba1c, Tubb4b, Uqcr10, Uqcr11, Uqcrc1, Uqcrc2, Uqcrfs1, Uqcrq, Vdac3, Xbp1*

Table S4 - Genes within KEGG Pathways related to ribosome biogenesis that are dysregulated by PAE

Gene Name	Fold Change (Alc/Control)	*P* value	*P adj*	Gene
rRNA Synthesis - KEGG 03020				
Eif4a1	0.90	5.57E-03	0.031	eukaryotic translation initiation factor 4A family member
Eif4a2	0.95	8.02E-03	0.040	eukaryotic translation initiation factor 4A family member
Eif4ebp1	0.67	3.03E-03	0.020	eukaryotic translation initiation factor 4A family member
Eif4g3	1.06	2.43E-03	0.017	eukaryotic translation initiation factor 4A family member
Polr1d	0.93	1.03E-02	0.048	polymerase (RNA) 1 polypeptide D
Polr2d	0.95	2.45E-03	0.017	polymerase (RNA) 2 polypeptide D
Polr2e	0.92	1.84E-04	0.0028	polymerase (RNA) 2 polypeptide E
Polr2f	0.91	6.54E-04	0.0070	polymerase (RNA) 2 polypeptide F
Polr2g	0.94	4.26E-03	0.026	polymerase (RNA) 2 polypeptide G
Polr2h	0.91	2.19E-03	0.016	polymerase (RNA) 2 polypeptide H
Polr2i	0.94	2.35E-03	0.017	polymerase (RNA) 2 polypeptide I
Polr2m	0.90	1.09E-04	0.0019	polymerase (RNA) 2 polypeptide M
Polr3d	0.89	1.07E-02	0.050	polymerase (RNA) 3 polypeptide D
Rps6ka3	1.11	5.20E-03	0.030	RPS6 Kinase alpha3 subunit
Rps19bp1	0.90	1.42E-05	0.00043	RPS19 binding protein 1
rRNA Processing & Assembly - KEGG 03008				
Csnk1g1	1.08	1.20E-06	7.20E-05	casein kinase 2, gamma 1 polypeptide
Csnk1g2	0.93	1.15E-03	0.010	casein kinase 2, gamma 1 polypeptide
Csnk2b	0.96	2.11E-03	0.016	casein kinase 2, gamma 1 polypeptide
Ddx1	0.92	2.18E-03	0.016	DEAD (Asp-Glu-Ala-Asp) box polypeptide 1
Ddx17	1.08	3.59E-03	0.023	DEAD (Asp-Glu-Ala-Asp) box polypeptide 17
Ddx6	1.03	5.80E-03	0.032	DEAD (Asp-Glu-Ala-Asp) box polypeptide 6
Ddx19b	0.99	4.59E-03	0.027	DEAD (Asp-Glu-Ala-Asp) box polypeptide 19B
Ddx24	0.92	8.01E-03	0.040	DEAD (Asp-Glu-Ala-Asp) box polypeptide 24
Ddx25	0.89	1.32E-07	1.44E-05	DEAD (Asp-Glu-Ala-Asp) box polypeptide25
Ddx28	0.89	7.64E-04	0.0078	DEAD (Asp-Glu-Ala-Asp) box polypeptide 28
Ddx39	0.87	9.44E-03	0.045	DEAD (Asp-Glu-Ala-Asp) box polypeptide 39
Ddx39b	0.94	1.08E-03	0.0099	DEAD (Asp-Glu-Ala-Asp) box polypeptide 39B
Ddx49	0.89	6.12E-03	0.033	DEAD (Asp-Glu-Ala-Asp) box polypeptide 49
Ddx54	0.86	1.19E-04	0.0020	DEAD (Asp-Glu-Ala-Asp) box polypeptide 54
Ddx59	0.85	1.98E-04	0.0029	DEAD (Asp-Glu-Ala-Asp) box polypeptide 59
Eif1	0.97	1.77E-03	0.014	eukaryotic translation initiation factor 1
Eif2ak2	1.20	6.78E-04	0.0072	eukaryotic translation initiation factor 2 Alpha Kinase 2
Eif2b1	0.92	1.86E-03	0.014	eukaryotic translation initiation factor 2B, subunit 1
Eif2b2	0.96	7.89E-03	0.040	eukaryotic translation initiation factor 2B, subunit 2
Eif2b4	0.92	1.35E-03	0.012	eukaryotic translation initiation factor 2B, subunit 4
Eif2b5	0.88	1.18E-05	0.00038	eukaryotic translation initiation factor 2B, subunit 5
Eif2s2	0.91	1.34E-05	0.00042	eukaryotic translation initiation factor S, subunit beta
Eif3b	0.91	2.37E-05	0.00063	eukaryotic translation initiation factor 3, subunitB
Eif3d	0.89	1.07E-06	6.59E-05	eukaryotic translation initiation factor 3, subunit D
Eif3g	0.94	5.56E-08	7.92E-06	eukaryotic translation initiation factor 3, subunit G
Eif3h	0.92	8.76E-06	0.00031	eukaryotic translation initiation factor 3, subunit H
Eif3i	0.89	1.36E-04	0.0022	eukaryotic translation initiation factor 3, subunit I
Eif3k	0.93	6.19E-06	0.00024	eukaryotic translation initiation factor 3, subunit K
Eif3l	0.95	2.60E-04	0.0036	eukaryotic translation initiation factor 3, subunit L
Eif4a1	0.90	5.57E-03	0.031	eukaryotic translation initiation factor 4A1
Eif4a2	0.95	8.02E-03	0.040	eukaryotic translation initiation factor 4A2
Eif4ebp1	0.67	3.03E-03	0.020	eukaryotic translation initiation factor 4E binding protein 1
Eif4g3	1.06	2.43E-03	0.017	eukaryotic translation initiation factor 4 Gamma 3
Eif5a	0.91	1.70E-04	0.0026	eukaryotic translation initiation factor 5A
Imp3	0.94	2.39E-03	0.017	U3 small nucleolar ribonucleoprotein, IMP3-like
Larp4	1.09	2.27E-03	0.016	La ribonucleoprotein domain famly, member 4
Larp7	0.91	5.58E-03	0.031	La ribonucleoprotein domain famly, member 5
Nhp2	0.86	2.98E-06	0.00014007	NHP2 ribonucleoprotein homolog, H/ACA ribonucleoproiten complex subunit 2
Nob1	0.92	5.91E-03	0.033	NIN1/RPN12 binding protein 1 homolog
Nol12	0.85	6.07E-05	0.0012	nucleolar protein 12
Nol7	0.91	7.73E-06	0.00028	nucleolar protein 7
Nop10	0.92	1.31E-04	0.0022	NOP10 ribonucleoproiten homolog

Nsun7	1.46	1.69E-04	0.0026	NOP2/Sun domain family, member 7
Pno1	0.89	3.28E-04	0.0043	partner of NOB1 homolog
Pop5	0.90	6.62E-04	0.0071	processing of precursor 5, ribonuclease P/MRP subunit
Rpusd1	0.92	4.31E-03	0.026	RNA Pseudouridine Synthase Domain Containing 1
Rpusd2	1.04	8.04E-03	0.041	RNA Pseudouridine Synthase Domain Containing 2
Ran	0.89	6.24E-04	0.0068	RAN, member RAS oncognee family
Ranbp10	1.06	5.92E-04	0.0065	RAN binding protein 10
Ranbp3	0.90	4.93E-03	0.029	RAN binding protein 3
Ranbp3l	1.70	4.89E-06	0.00020	RAN binding protein 3-like
Rexo2	0.95	9.12E-03	0.044	REX2, RNA exonucleoase 2 homolog
Rrp1	0.92	6.02E-05	0.0012	ribosomal RNA processing 1
Rrp12	0.83	6.54E-03	0.035	ribosomal RNA processing 12
Rrp36	0.93	8.47E-03	0.042	ribosomal RNA processing 36
Rrp7a	0.88	5.28E-05	0.0011	ribosomal RNA processing 7A
Rrbp1	1.08	3.92E-03	0.024	Ribosome Binding Protein 1
Snrnp25	0.83	6.05E-05	0.0012	small nucleoar ribonucleoprotein 25 kDa
Snrnp27	0.96	7.92E-04	0.0080	small nucleoar ribonucleoprotein 27 kDa
Snrnp40	0.90	2.59E-03	0.018	small nucleoar ribonucleoprotein 40 kDa
Snrpa1	0.93	5.65E-03	0.032	small nucleoar ribonucleoprotein polypeptide A1
Snrpd1	0.94	2.13E-04	0.0031	small nucleoar ribonucleoprotein polypeptide D1
Snrpd2	0.91	4.74E-05	0.0011	small nucleoar ribonucleoprotein polypeptide D2
Snrpe	0.92	4.18E-03	0.025	small nucleoar ribonucleoprotein polypeptide E
Utp18	0.93	7.75E-03	0.040	UTP18 Small Subunit Processome Component
Wdr18	0.86	8.73E-06	0.00031	WD repeat domain 18, component of PELP1 of pre60S
Wdr26	1.05	6.02E-05	0.0012	WD repeat domain 26, component of PELP1 of pre60S
Wdr34	0.89	1.17E-03	0.010	WD repeat domain 34, component of PELP1 of pre60S
Wdr48	0.91	7.88E-04	0.0080	WD repeat domain 48, component of PELP1 of pre60S
Wdr74	0.90	3.85E-05	0.00090	WD repeat domain 74, component of PELP1 of pre60S
Wdr82	0.88	2.30E-12	7.55E-09	WD repeat domain 82, component of PELP1 of pre60S
Wdr86	1.28	3.71E-03	0.023	WD repeat domain 86, component of PELP1 of pre60S
Xpo1	1.10	5.99E-04	0.0066	exportin 1
Xrn1	1.14	2.55E-04	0.0035	5'-3' exoribonuclease 1
Mdm1	1.15	6.94E-03	0.036	Murine double minute-1
Mdm4	1.20	9.22E-04	0.0089	Murine double minute-4

tRNA Synthesis - KEGG 00970

Aars	0.84	2.49E-05	0.00065	Alanyl-TRNA Synthetase
Aars2	0.85	5.09E-03	0.029	Alanyl-TRNA Synthetase
Aarsd1	0.89	5.26E-05	0.0011	Alanyl-TRNA Synthetase Domain Containing 3
Cars	0.80	1.20E-06	7.20E-05	Cystinyl-TRNA Synthetase
Dars	0.89	1.91E-06	0.00010	Aspartyl-TRNA Synthetase
Farsa	0.91	8.25E-04	0.0082	Phenylalanyl-TRNA Synthetase, subunit alpha
Farsb	0.87	3.37E-09	9.69E-07	Phenylalanyl-tRNA synthetase, subunit beta
Gars	0.88	9.97E-07	6.34E-05	Glycyl-TRNA Synthetase
Hars	0.89	1.73E-05	0.00050	Hisdinyl-TRNA Synthetase
Kars	0.89	1.81E-06	9.74E-05	Lysyl-TRNA Synthetase
Mars	0.88	4.00E-04	0.0050	Methionyl-TRNA Synthetase
Mars2	1.14	2.75E-03	0.019	Methionyl-TRNA Synthetase 2
Nars	0.89	1.16E-03	0.010	Asparginyl-TRNA Synthetase
Sars	0.89	5.12E-08	7.55E-06	Serinyl-TRNA Synthetase
Sars2	0.84	5.59E-03	0.031	Serinyl-TRNA Synthetase 2
Tars2	0.85	1.56E-03	0.013	Threonyl-TRNA Synthetase
Wars	0.91	5.58E-05	0.0012	Tryptophenyl-TRNA Synthetase
Yars	0.83	7.97E-08	1.01E-05	Tyrosinyl-TRNA Synthetase
Yars2	0.88	3.42E-03	0.022	Tyrosinyl-TRNA Synthetase 2
Aimp1	0.95	2.81E-03	0.019	aminoacyl tRNA synthetase complex-interacting multifunctional protein-1
Aimp2	0.82	8.33E-09	1.91E-06	aminoacyl tRNA synthetase complex-interacting multifunctional protein-2
Pop5	0.90	6.62E-04	0.0071	processing of precursor 5, ribonuclease P/MRP subunit
Trmt112	0.93	1.70E-03	0.014	TRNA Methyltransferase Activator Subunit 11-2
Ptrhd1	0.89	1.73E-03	0.014	Peptidyl-TRNA Hydrolase Domain Containing 1

Ribosomal proteins - KEGG 04150

Rpl3	0.92	2.58E-07	2.27E-05	Ribosome protein L3
Rpl4	0.95	4.87E-04	0.0056	Ribosome protein L4
Rpl6	0.93	6.19E-04	0.0068	Ribosome protein L6
Rpl7	0.97	2.22E-03	0.016	Ribosome protein L7
Rpl8	0.91	3.16E-05	0.00079	Ribosome protein L8
Rpl10	0.96	5.88E-05	0.0012	Ribosome protein L10
Rpl10a	0.96	4.76E-03	0.028	Ribosome protein L10A
Rpl11	0.91	8.88E-06	0.00031	Ribosome protein L11
Rpl12	0.94	8.95E-03	0.044	Ribosome protein L12
Rpl13	0.94	1.89E-03	0.014	Ribosome protein L13
Rpl13a	0.87	3.44E-05	0.00083	Ribosome protein L13A
Rpl14	0.95	3.98E-03	0.025	Ribosome protein L14
Rpl15	0.90	2.59E-09	8.70E-07	Ribosome protein L15
Rpl18	0.86	4.53E-06	0.00019	Ribosome protein L18
Rpl18a	0.96	1.03E-02	0.048	Ribosome protein L18A
Rpl19	0.94	1.16E-03	0.010	Ribosome protein L19
Rpl22l1	0.87	4.65E-03	0.027	Ribosome protein L22-like 1
Rpl23	0.97	7.40E-03	0.038	Ribosome protein L23
Rpl23a	0.99	6.19E-03	0.034	Ribosome protein 23A
Rpl27	0.95	4.62E-03	0.027	Ribosome protein L27
Rpl27a	0.93	1.29E-04	0.0022	Ribosome protein L27A
Rpl28	0.93	3.11E-06	0.00014	Ribosome protein L28
Rpl29	0.95	1.64E-05	0.00048	Ribosome protein L29
Rpl30	0.86	3.16E-03	0.021	Ribosome protein L30
Rpl31	0.89	1.13E-03	0.010	Ribosome protein L31
Rpl35	0.95	2.16E-03	0.016	Ribosome protein L35
Rpl36	0.91	1.17E-03	0.010	Ribosome protein L56
Rpl36a	0.89	5.74E-04	0.0064	Ribosome protein L36A
Rpl36al	0.95	8.29E-05	0.0015	Ribosome protein L36A like
Rpl37	0.87	2.16E-05	0.00059	Ribosome protein L37
Rpl38	0.88	3.49E-03	0.022	Ribosome protein L38
Rpl39	0.88	1.54E-03	0.013	Ribosome protein L39
Rpl41	0.87	1.56E-07	1.65E-05	Ribosome protein L41
Rplp0	0.91	2.45E-04	0.0034	Ribsomal protein lateral stalk subunity P0
Rplp1	0.91	1.89E-05	0.00054	Ribsomal protein lateral stalk subunity P1
Rpp30	0.92	5.93E-03	0.033	Ribonuclease P/MRP Subunit P30
Rpp40	0.70	3.73E-04	0.0047	Ribonuclease P/MRP Subunit P40
Rps2	0.84	6.95E-05	0.0014	Ribosome protein S2
Rps3	0.85	1.32E-04	0.0022	Ribosome protein S3
Rps5	0.88	1.03E-04	0.0018	Ribosome protein S5
Rps6	0.95	4.93E-03	0.029	Ribosome protein S6
Rps7	0.89	5.74E-05	0.0012	Ribosome protein S7
Rps8	0.94	1.41E-03	0.012	Ribosome protein S8
Rps9	0.90	3.04E-03	0.020	Ribosome protein S9
Rps10	0.94	1.86E-04	0.0028	Ribosome protein S10
Rps11	0.97	1.55E-03	0.013	Ribosome protein S11
Rps13	0.92	8.13E-03	0.041	Ribosome protein S14
Rps15	0.91	1.53E-05	0.00046	Ribosome protein S15
Rps16	0.90	8.81E-04	0.0086	Ribosome protein S16
Rps17	0.92	9.63E-04	0.0092	Ribosome protein S17
Rps18	0.92	1.93E-03	0.015	Ribosome protein S18
Rps20	0.94	8.93E-04	0.0086	Ribosome protein S20
Rps21	0.87	8.19E-05	0.0015	Ribosome protein S21
Rps23	0.92	1.88E-04	0.0028	Ribosome protein S23
Rps24	0.95	5.49E-05	0.0012	Ribosome protein S24
Rps27a	0.91	3.07E-03	0.020	Ribosome protein 27A
Rps28	0.89	8.03E-03	0.041	Ribosome protein S28
Rps29	0.88	2.38E-04	0.0034	Ribosome protein S29
Rpsa	0.86	1.09E-05	0.00036	Ribosome protein SA
Mrpl2	0.94	2.96E-03	0.020	Mitochondrial ribosome protein L2
Mrpl3	0.95	1.19E-03	0.011	Mitochondrial ribosome protein L3
Mrpl9	0.93	7.00E-03	0.037	Mitochondrial ribosome protein L9
Mrpl10	0.91	1.10E-03	0.010	Mitochondrial ribosome protein L10

Mrpl11	0.83	1.06E-08	2.25E-06	Mitochondrial ribosome protein L11
Mrpl12	0.88	4.28E-06	0.00018	Mitochondrial ribosome protein L12
Mrpl13	0.95	3.42E-03	0.022	Mitochondrial ribosome protein L13
Mrpl14	0.95	4.14E-03	0.025	Mitochondrial ribosome protein L14
Mrpl15	0.80	6.64E-08	9.07E-06	Mitochondrial ribosome protein L15
Mrpl18	0.94	1.61E-03	0.013	Mitochondrial ribosome protein L18
Mrpl19	0.83	8.13E-04	0.0081	Mitochondrial ribosome protein L19
Mrpl20	0.91	6.48E-05	0.0013	Mitochondrial ribosome protein L20
Mrpl22	0.90	2.35E-03	0.017	Mitochondrial ribosome protein L22
Mrpl27	0.88	4.33E-05	0.000976504	Mitochondrial ribosome protein L27
Mrpl28	0.93	7.23E-03	0.038	Mitochondrial ribosome protein L28
Mrpl33	0.89	6.63E-06	0.00025	Mitochondrial ribosome protein L33
Mrpl34	0.90	2.49E-07	2.24E-05	Mitochondrial ribosome protein L34
Mrpl37	0.91	9.92E-04	0.0094	Mitochondrial ribosome protein L37
Mrpl38	0.92	3.35E-05	0.00082	Mitochondrial ribosome protein L38
Mrpl40	0.91	7.69E-05	0.0015	Mitochondrial ribosome protein L40
Mrpl41	0.86	1.95E-03	0.015	Mitochondrial ribosome protein L41
Mrpl43	0.91	3.20E-06	0.00014	Mitochondrial ribosome protein L43
Mrpl45	0.86	1.24E-04	0.0021	Mitochondrial ribosome protein L45
Mrpl46	0.91	1.54E-04	0.0024	Mitochondrial ribosome protein L46
Mrpl48	0.88	1.01E-02	0.048	Mitochondrial ribosome protein L48
Mrpl50	0.95	8.98E-03	0.044	Mitochondrial ribosome protein L50
Mrpl52	0.89	6.83E-05	0.0013	Mitochondrial ribosome protein L52
Mrpl53	0.94	6.16E-03	0.033	Mitochondrial ribosome protein L53
Mrpl54	0.80	7.46E-07	5.09E-05	Mitochondrial ribosome protein L54
Mrps6	0.88	6.11E-08	8.51E-06	Mitochondrial ribosome protein S6
Mrps7	0.86	3.64E-06	0.000159927	Mitochondrial ribosome protein S7
Mrps10	0.92	5.82E-03	0.032	Mitochondrial ribosome protein S10
Mrps11	0.92	7.71E-04	0.0079	Mitochondrial ribosome protein S11
Mrps12	0.88	6.60E-07	4.67E-05	Mitochondrial ribosome protein S12
Mrps17	0.95	5.69E-04	0.0063	Mitochondrial ribosome protein S17
Mrps18a	0.88	3.22E-05	0.00080	Mitochondrial ribosome protein S18A
Mrps18b	0.89	6.50E-04	0.0070	Mitochondrial ribosome protein S18B
Mrps21	0.93	2.15E-03	0.016	Mitochondrial ribosome protein S21
Mrps22	0.93	1.93E-03	0.015	Mitochondrial ribosome protein S22
Mrps23	0.95	1.48E-03	0.012	Mitochondrial ribosome protein S23
Mrps24	0.84	7.91E-05	0.0015	Mitochondrial ribosome protein S24
Mrps25	0.78	1.87E-04	0.0028	Mitochondrial ribosome protein S25
Mrps26	0.94	6.37E-03	0.034	Mitochondrial ribosome protein S26
Mrps27	0.84	2.53E-04	0.0035	Mitochondrial ribosome protein S27
Mrps28	0.81	1.59E-05	0.00047	Mitochondrial ribosome protein S28
Mrps30	0.92	2.91E-04	0.0039	Mitochondrial ribosome protein S30
Mrps31	0.86	4.36E-05	0.000980284	Mitochondrial ribosome protein S31
Mrps33	0.92	5.03E-03	0.029	Mitochondrial ribosome protein S33
Mrps34	0.96	1.04E-03	0.0097	Mitochondrial ribosome protein S34
Mrps35	0.95	5.47E-03	0.031	Mitochondrial ribosome protein S35

Table S5 - List of P53-related genes dysregulated by alcohol in E17.5 fetal brain

Gene	Fold-Change, ALC/CON	P-value	P-adj value
Aen	0.83	7.89E-06	0.00029
Afp	6.51	1.84E-10	1.34E-07
Aifm3	1.33	2.88E-05	0.00074
Apaf1	1.03	6.12E-04	0.0067
Apc	1.07	3.50E-05	0.00085
Bax	0.95	9.79E-03	0.047
Bcl2	1.03	6.34E-06	0.00024
Cbl	1.16	9.30E-09	2.07E-06
Cers6	1.12	1.18E-04	0.0020
Col18a1	1.36	3.08E-06	0.00014
Csf1	1.14	2.80E-03	0.019
Cst3	0.84	3.62E-05	0.00086002
Cycs	0.87	1.02E-02	0.048
Dsp	1.26	8.75E-04	0.0085
Dut	0.84	7.74E-05	0.0015
Ercc1	0.90	3.20E-03	0.021
Fdft1	0.82	1.29E-06	7.58E-05
Fdps	0.86	8.61E-05	0.0016
Fuca1	0.90	9.91E-03	0.047
Gadd45gip1	0.87	2.94E-05	0.00075
Gamt	0.83	2.22E-03	0.016
Hic1	1.23	8.19E-04	0.0081
Hmgcr	0.88	7.86E-03	0.040
Hspa8	0.90	4.23E-04	0.0051
Htt	1.00	3.57E-05	0.00086
Irf2bp2	1.09	5.09E-03	0.029
Lonp1	0.90	3.63E-04	0.0046
Mdm1	1.15	6.94E-03	0.036
Mdm4	1.20	9.22E-04	0.0089
Mmp2	1.27	1.96E-06	0.00010274
Mpzl2	1.66	8.86E-04	0.0086
Mvk	0.79	3.33E-05	0.00082
Ndrg1	1.14	1.22E-03	0.011
Notch1	1.03	2.96E-03	0.020
Nov	1.24	1.12E-04	0.0019
Nsg1	0.93	5.00E-03	0.029
Ogn	1.34	8.31E-03	0.041
Pank1	0.83	3.85E-04	0.0048
Pebp1	0.91	5.75E-03	0.032
Pik3ca	1.00	5.93E-03	0.033
Pkd1	0.98	3.90E-04	0.0049
Plagl1	1.20	4.59E-04	0.0054
Pml	1.07	1.92E-03	0.015

Pten	0.99	9.95E-03	0.047
Rabggta	0.87	7.29E-03	0.038
Rad23a	0.92	1.23E-03	0.011
Rgcc	0.85	5.31E-03	0.030
Sema3f	1.14	1.32E-03	0.011
Steap3	1.22	2.98E-03	0.020
Tgm2	1.37	9.72E-06	0.00033
Ubtd1	0.86	1.01E-02	0.048
Xpo1	1.10	5.99E-04	0.0066
Zmat3	1.04	2.02E-05	0.00057
